# How Host Phylogeny and Diet Shape the Specificity and Specificity Diversity of Animal Gut Microbiomes

**DOI:** 10.1111/1758-2229.70253

**Published:** 2026-01-29

**Authors:** Zhanshan (Sam) Ma

**Affiliations:** ^1^ Faculty of Arts and Sciences, Harvard Forest Harvard University Cambridge Massachusetts USA; ^2^ Computational Biology and Medical Ecology Lab, Biostatistics and Image Genetics Lab Kunming Institute of Zoology, Chinese Academy of Sciences Kunming China; ^3^ Microbiome Medicine and Advanced AI Technology Kunming China

**Keywords:** animal gastrointestinal tract microbiomes (AGM), host specific (unique or enriched) microbial species, phylogenetic timeline (PT), species specificity (SS), species specificity and specificity diversity (SSD) framework, specificity diversity (SD)

## Abstract

The forces shaping host specificity in the animal gastrointestinal microbiome (AGM) are often studied through separate lenses: community‐level patterns (phylosymbiosis) or lineage‐level histories (cophylogeny). Furthermore, traditional diversity metrics fail to capture compositional heterogeneity from host‐specific distributions. We bridge these gaps using our SSD (Species Specificity and Specificity Diversity) framework, a recent conceptual and computational advance that quantifies host specificity across scales via: (i) Species Specificity (SS), locating species on the specialist‐generalist continuum; (ii) Specificity Diversity (SD), quantifying community compositional heterogeneity; and (iii) statistical tests for identifying unique/enriched species. Applying SSD to 4903 AGM samples from 318 species, we identified unique and enriched microbial species in specific host taxa and diets, demonstrating that host phylogeny and diet jointly shape these patterns. A PTSD (Phylogenetic Timeline–Specificity Diversity) power‐law model reveals the evolution of more complex microbiome structures in modern species. One surprising finding is the high similarity amongst animal AGMs, with only 252 microbial species being exclusively unique at the animal class level—somewhat analogous to the high genomic similarity between humans and primates. Our findings demonstrate a unified quantitative approach to dissecting the eco‐evolutionary forces that shape microbial specificity and specificity heterogeneity, with potential synthesis with established phylosymbiosis and cophylogeny frameworks.

## Graphical abstract

By identifying unique/enriched microbial species across taxa and diet types in 318 animal species with our SSD (specificity and specificity diversity) framework, we demonstrated that host phylogeny and diet are joint drivers of microbial specificity compositional heterogeneity, potentially linking the macroecological pattern of phylosymbiosis with the microevolutionary processes of cophylogeny.

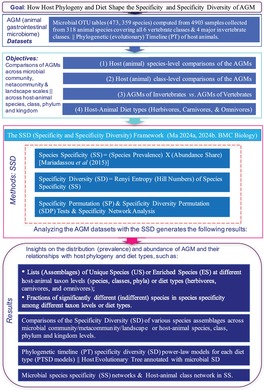

## 1 Introduction

The animal gastrointestinal microbiome (AGM) is a complex community critical to host health, evolution, and function. The holobiont concept, which views a host and its symbiotic microbes as a single ecological and evolutionary unit, provides a framework for understanding these relationships (Rosenberg et al. 2009; Brucker and Bordenstein 2013; Theis et al. 2016; Rosenberg and Zilber‐Rosenberg 2018; Carthey et al. 2019). A key question in holobiont biology is the degree of host‐specificity—how and why microbial communities are associated with particular hosts. Two major factors shaping AGM composition and host‐specificity are host evolutionary history (phylogeny) and diet, which are deeply intertwined (Ley et al. 2008; Muegge et al. 2011; Carmody et al. 2015; Youngblut et al. 2019; Ma 2022).

Research into host‐specificity has often proceeded along two parallel paths. One path, exemplified by the concept of phylosymbiosis, focuses on community‐level patterns, asking whether the evolutionary relationships between hosts are reflected in the overall similarity of their microbial communities (Brooks et al. 2016). The other path, cophylogeny, investigates lineage‐level history, testing whether individual microbial taxa have diversified in parallel with their hosts over evolutionary time (Moeller et al. 2016). Whilst these frameworks have been highly productive, a gap remains in quantitatively bridging the specific contributions of individual microbial taxa with the emergent patterns observed at the whole‐community level.

Extensive research has established links between AGM diversity, host phylogeny, and diet (e.g., Delsuc et al. 2014; Groussin et al. 2017; Mazel et al. 2018; Amato et al. 2019; Song et al. 2020). For instance, phylogeny can dictate the heritability of fine‐scale microbial taxa, whilst diet acts as an environmental filter on functional guilds (Youngblut et al. 2019). Furthermore, quantitative models have been established between AGM diversity and the host Phylogenetic Timeline (PT), a measure of a taxon's evolutionary age, revealing patterns across the animal kingdom (Ma 2022).

However, traditional diversity metrics, which summarise species abundance distributions, may not fully capture the complex nature of host‐microbe associations. As an analogy, economic indices like average income or the Gini coefficient are insufficient to characterise a full economy. Similarly, diversity metrics often overlook species interactions and the distribution of species across different hosts, which are key to understanding community heterogeneity (Ma and Ellison 2024; Ma 2025). A zoo is diverse, with animals in cages having little interaction, whilst an ecosystem is heterogeneous, a state defined by the interactions amongst its constituents (Shavit and Ellison 2021; Ma 2025). This constitutes a critical gap: the need for metrics that can quantify the heterogeneity of microbial communities in a way that reflects the outcomes of species interactions and host‐specific selection.

**FIGURE 1:**
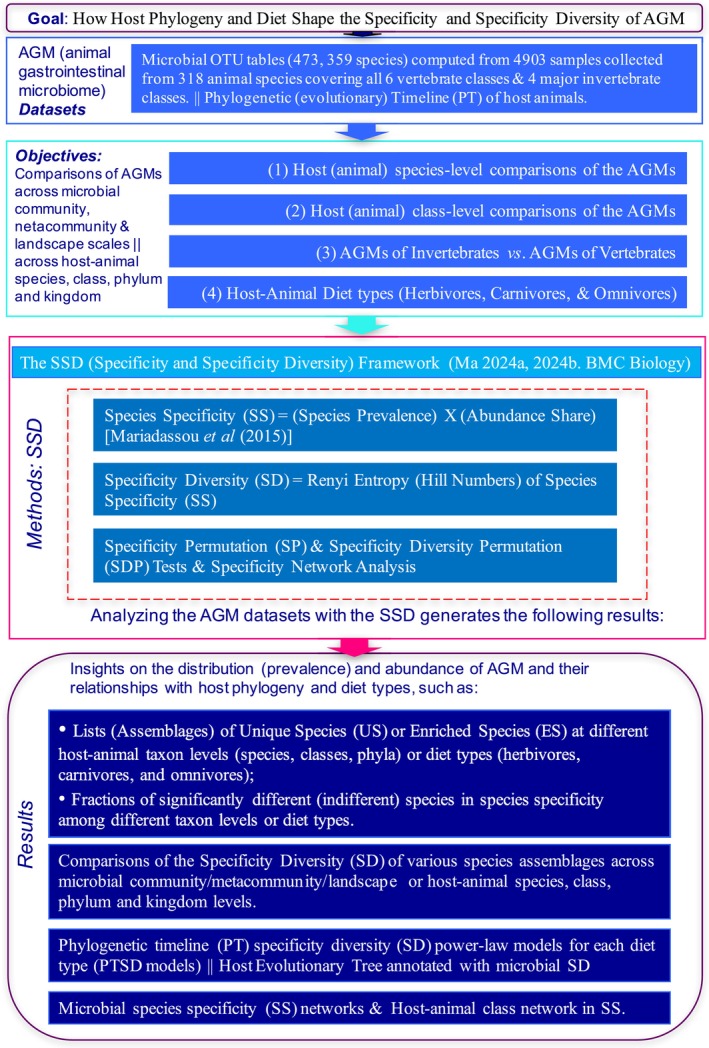
Study design for the AGM (animal gastrointestinal microbiome) species specificity and specificity diversity analyses with 473,359 microbial species, from 4903 AGM samples of 318 host animal species, covering all 6 vertebrate classes and 4 major invertebrate classes and three diet types: Five blocks from the top to bottom: The goal, datasets, objectives, methods, and results.

To address this, we employ the Species Specificity and Specificity Diversity (SSD) Framework (Ma 2024a, 2024b). This framework moves beyond local diversity to quantify how microbial distributions are shaped across a landscape of different host ‘habitats,’ thereby bridging the gap between community‐level patterns and taxon‐level distributions. The SSD is built on two core metrics that quantify host‐specificity at different scales: (i) Species Specificity (SS), which measures the degree to which a single microbial taxon is restricted to a particular host or host group. (ii) Specificity Diversity (SD), which integrates the SS values of all taxa within a community to measure its overall compositional heterogeneity relative to other communities. Furthermore, the framework includes rigorous statistical tests that are applied to these metrics to filter out noise and ensure the identified specificity patterns are statistically significant. A brief introduction to the core concepts, metrics, and algorithms of the SSD Framework is provided in Box 1, Box 2, and Figure 1.

In this study, we apply the SSD framework to a large‐scale dataset of over 4900 animal gastrointestinal microbiome (AGM) samples spanning 318 animal species (Ma 2022). Our objectives are threefold: (i) to quantify microbial specificity and specificity diversity across a hierarchy of host taxa—from species to class levels—and major diet types, thereby mapping patterns of host specificity; (ii) to statistically identify unique (US) and enriched (ES) microbial species characteristic of these distinct host habitats; and (iii) to model the relationship between host evolutionary history, using the Phylogenetic Timeline (PT), and community‐level heterogeneity, measured by Specificity Diversity (SD). This final aim directly tests the interplay of phylogeny and diet in structuring host‐specific microbial communities, providing a quantitative assessment of their relative roles in shaping the AGM landscape.

BOX 1 **Key Concepts and Metrics of the Species Specificity and Specificity‐Diversity (SSD) Framework.**

**Table:** 

No.	Item	Concept	Metric/Note
1	Species Specificity and Specificity‐Diversity (SSD) Framework	The SSD provides a unified methodology that harnesses heterogeneity in species abundance and distribution across both species and community scales. This framework is designed to detect habitat‐specific species (or taxa), including unique or enriched species (US/ES), and to holistically compare the compositional heterogeneity of species assemblages (communities).	SSD consisting of Items (#2–#4) below (Ma 2024a, 2024b)
2	Species Specificity (SS)	Species Specificity (SS) metric, proposed by Mariadassou et al. (2015), quantifies the specificity of a species or taxon. It is a modern reincarnation of the generalist‐specialist continuum (Dufrene and Legendre 1997) that differentiates itself from traditional metrics by incorporating species abundance and distribution across alternative habitats. Its values range from 0 to 1, where 0 indicates a species' absence from the local habitat and 1 signifies its exclusive presence, representing an extreme specialist or a perfect indicator species.	*See* Equation (1) in the Methods section and Computational Algorithm and Procedure in Box 2.
3	Specificity Diversity (SD)	Specificity Diversity (SD): Proposed by Ma (2024a, 2024b), SD aggregates the SS values of all species within an assemblage, local community or metacommunity. Using Renyi's entropy in the form of Hill numbers (Renyi 1961; Hill 1973; Chao et al. 2014), it summarises the species specificity distribution, providing a comprehensive measure of the difference between assemblages from a specificity perspective. The SD provides a metric to comprehensively measure the compositional heterogeneity (differences) between two species assemblages from a specificity perspective.	*See* Equation (2) and Box 2.
4	Specificity Permutation (SP) Test	By leveraging the power of permutation tests, the Specificity Permutation (SP) test extends the SS metric into a framework of theoretical rules for identifying treatment‐specific taxa (e.g., unique or enriched species). This method filters out stochastic noise to robustly recover the true heterogeneity attributable to treatments.	*See* Box 2 *for details*.
*5*	Specificity Diversity Permutation (SDP)	The Specificity Diversity Permutation (SDP) test uses permutation tests to compare compositional heterogeneity—calculated as the entropy of species specificities—across different assemblages (e.g., communities, metacommunities, and landscapes). It provides an alternative to traditional diversity comparisons by directly incorporating heterogeneity of specificity, making it more effective than species diversity metrics at discerning treatment effects holistically.	*See* Box 2 *for details*.
*6*	Phylogenetic Timeline (PT)	Phylogenetic Time (PT), or the evolutionary timeline, is distinct from more familiar Phylogenetic Distance (PD) and is more suitable for modelling relationships between animal gut microbiome (AGM) diversity and host phylogeny. This is because PT represents the absolute ‘age’ of a taxon, where ancient species possess larger values (Kumar et al. 2017; Ma 2022). In contrast, PD signifies the relative divergence time between a pair of species, which provides a less convenient framework for this specific analysis.	*See* Equations (3) and (4) for the PT‐SD power‐ law scaling relationship.
*7*	Diversity vs. Heterogeneity	Diversity and heterogeneity are often conflated. Diversity quantifies the number of species, offering a simple, convenient measure by ignoring species interactions. Heterogeneity, in contrast, emphasises that species interactions are critical to community structure and dynamics and cannot be ignored. This makes heterogeneity far more difficult to measure, and no standard metric for it yet exists. The term itself remains ambiguous and is often used loosely to mean group ‘differences’ (Shavit and Ellison 2021, Ma and Ellison 2024, Ma 2025). Defining an unambiguous concept and/or ideal metric for heterogeneity is beyond the scope of this article. Instead, we consider the aggregation of species specificity with an entropy function as essentially a proxy of compositional heterogeneity from the specificity perspective. The justification for accepting this proxy measure of heterogeneity rests on a first principle: the information synthesised by the entropy function (Equation 2), particularly regarding species distribution, is itself a consequence of species interactions.	*See* Equation (2) and Box 2.

## 2 Material and Methods

### 2.1 Animal Gastrointestinal Tract Microbiome (AGM) Datasets and Phylogenetic Timeline (PT)

We gathered datasets of 16S‐rRNA sequencing reads from over 6900 samples of the animal gastrointestinal microbiome (AGM) from 108 published studies, encompassing 5 phyla and 19 classes. After conducting quality control and excluding samples from non‐gastrointestinal tracts, a total of 4903 samples remained. These samples cover 3 primary phyla (*Nematoda*, *Arthropoda*, and *Chordates*), 10 classes (*Chromadorea, Arachnida, Malacostraca, Insecta, Chondrichthyes, Actinopteri, Amphibia, Sauropsida*, *Aves*, and *Mammalia*), and 318 animal species, and were used for this study. Consequently, the chosen samples encompass all six classes of vertebrates and the two most significant phyla of invertebrates (*Nematoda* and *Arthropoda*), ensuring that the datasets adequately represent the animal kingdom.

A summary table for the 4903 samples, detailing their taxonomic distribution and diet‐type classifications, was previously provided in Table S7 of Ma (2022). That table has been further supplemented with additional information in the present article and is included as Table S8. Specifically, in Table S8, we provide the mean numbers of reads and OTUs per sample, as well as the total numbers of OTUs and samples for each major taxon or diet type.

To summarise some basic information, the 4903 AGM samples were spread across 10 animal classes as previously mentioned, 59 orders, 142 families, 261 genera, and 318 species. Regarding species distribution, Mammalia and Insecta had the most significant representation with 123 species (1499 samples) and 76 species (979 samples), respectively, accounting for 62% of the total species covered by all 4903 samples. In terms of diet types, out of the 4903 AGM samples, 1421, 1229, and 1473 samples belong to carnivore, herbivore, and omnivore groups, respectively. The remaining unclassified samples (320) were grouped into the ‘Other’ category.

To address the potential heterogeneity of the data source, we used QIIME‐2 (Version 2018.6.0, Bolyen et al. 2018) to recalculate the OTU tables (at the 97% or species similarity level) from the 16S‐rRNA sequencing reads, originally published in 108 publications. This recalculation with the QIIME‐2 bioinformatics pipeline yielded a total of 473,359 microbial species. We obtained the phylogenetic time (PT), also known as the evolutionary time (ET), information of host animals from http://timetree.org (Kumar et al. 2017). This website provides two types of phylogenetic information: the divergence time for a pair of taxa, known as phylogenetic distance (PD), and the ‘evolutionary time (ET) or phylogenetic timeline (PT) of a taxon,’ which we utilised for this study. The PT can be viewed as a proxy for the phylogenetic history of a taxon, in our case, an animal species. It can also be interpreted as the ‘age’ of a taxon, with ancient taxa having larger PT values and modern taxa having smaller PT values. We deemed PT more suitable for this study, as it is associated with each taxon, allowing us to compute specificity diversity or other statistics of species specificity. The software packages we used to analyze and visualise the PT information include the APE (Paradis et al. 2004) and GGTREE (Yu et al. 2017) R‐packages.

### 2.2 Species Specificity and Specificity Diversity (SSD) Framework

Note that in this section, for the purpose of explaining the SSD (Figure 1), the term ‘species’ is used to denote a pure taxonomic unit. This can refer to microbial species, animal species, or even an Operational Taxonomic Unit (OTU) of microbes, depending on the taxon level or type used for SSD analysis. For instance, if the SSD is applied to detect unique microbial phyla, then ‘species’ would actually refer to unique phyla. In this article, the SSD is applied only to microbial taxa, not to the host animals that act as hosts or habitats of microbes.

The Species Specificity (SS) of Mariadassou et al. (2015) can be decomposed as the product of species prevalence and species abundance in a cross‐habitats setting. Assuming $M=\left[a_{\mathrm{ij}}\right]$ be the OTU table representing the compositions of microbiota, where $a_{\mathrm{ij}}$ is the relative abundance of species *i* in sample *j*. Let *H* be the number of different habitats (e.g., different host species of AGM), *S* ^*h*^ be the number of samples obtained from habitat *h*, *S*_*i*_^*h*^ be the number of samples from habitat *h* where species *i* is detected. The local species specificity is defined as the following product of species prevalence ($A_{i}^{h}$) and abundance $\left(B_{i}^{h}\right)$:

(1) $$\Delta_{i}^{h}=A_{i}^{h}\times B_{i}^{h}$$

in which $A_{i}^{h}=\frac{S_{i}^{h}}{S^{h}}$, $B_{i}^{h}=\frac{<a_{i}{>}^{h}}{\sum_{h=1}^{H}<a_{i}{>}^{h}}$, and $<a_{i}{>}^{h}=\frac{\sum_{j=1}^{S^{h}}a_{\mathrm{ij}}}{S^{h}}.$

Obviously, $A_{i}^{h}$ represents the *prevalence* or *distribution* of species *i* in habitat *h*, namely, the proportion (fraction) of samples from habitat *h* where species *i* was detected. $<a_{i}{>}^{h}$ denotes the average local abundances of species *i* in habitat *h*, and $B_{i}^{h}$ denotes the relative abundance share of habitat *h* in the total population of species *i* across all habitats (*H*).

It is noted that $\Delta_{i}^{h}\in \left[0,1\right]$ with zero indicating that the species is absent in local habitat *h*, with one indicating that the species always exists and only exists in that habitat, i.e., a perfect indicator of that (local) habitat or an extreme specialist to that habitat. Whilst an extreme specialist has a specificity value of $\Delta_{i}^{h}$ = 1, an extreme generalist does not have a value of $\Delta_{i}^{h}$ = 0, instead, $\Delta_{i}^{h}=1/H$, which means that prevalence = 1, with equal abundance in all habitats.

In the context of this study, we can consider each animal species as a different habitat type (*h*) that supports host‐species‐specific microbiomes, and 318 animal species in our datasets then represent *H* = 318 different habitats for the AGMs. Each habitat (animal species) was sampled *S* ^*h*^ times (*S* ^*h*^ samples), which are usually taken from multiple individuals of the animal species.

With this ‘natural’ setting, all community samples from the same animal species (habitat *h*) can be considered as a metacommunity, and the *H* metacommunities constitute the *landscape* of AGMs, from a microbial perspective, which correspond to the animal kingdom from the host perspective.

Alternatively, we can define the habitat scale differently from the previous natural scale of host animal species. In the study (Figure 1), we also treat animal class as a habitat type, and a total of 10 classes (*H* = 10 habitats) are compared with each other regarding their AGM compositions. We further treat invertebrates and vertebrates as two different habitat types (*H* = 2) to compare the difference between them in microbiome compositions. Finally, we compare three different diet types (carnivores, herbivores, and omnivores) in their AGM compositions by treating each diet type as a separate habitat type (*see* Figure 1 for the detailed designs of these alternatives).

Note that the definition of Species Specificity (SS) is both species‐ and habitat‐specific, and its computation also includes inputs from alternative habitats. In the context of this study, each microbial species can potentially have a different specificity value in each habitat (animal host). This differs from species abundance, which is a local property and does not take into account input from alternative habitats.

The Specificity Diversity (SD) proposed by Ma (2024a, 2024b) is designed to summarise the Species Specificity (SS) of a species assemblage, which could be a local community, a metacommunity of multiple local communities, or simply a group of species. The mathematical structure used to define the SD is Renyi's (1961) entropy, which was previously used to measure species diversity in ecology by Hill (1973) but received little attention until it was reintroduced to ecology by Chao et al. (2012, 2014). A key advantage of the Hill numbers or Renyi entropy is that virtually all existing biodiversity metrics can be represented by Hill numbers at different diversity orders (*q* = 0, 1, 2, …) (Chao et al. 2014). More importantly, all Hill numbers are in the unit of numbers of species or species equivalents such as OTUs. In other words, all Hill numbers are of the same base quantity (fundamental unit). The Specificity Diversity (SD) for a species assemblage (A) of diversity order *q* is defined as follows:
(2)

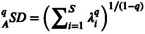

 where *S* is the number of species in species assemblage *A*, $\lambda_{i}$ = $\Delta_{i}$/$\sum_{i=1}^{S}\Delta_{i}$ is the *relative specificity* of species *i*, *q* is the order number of specificity diversity. As previously explained, a species assemblage can be a community, a metacommunity, or simply a group of species. However, since the specificity list is habitat‐specific, the potential or maximal sizes of species assemblage *A* should be limited to the number of species supported by a local habitat *h* (e.g., all AGM species of a host animal species). Therefore, SD can be used to summarise species composition information (in the form of species specificity) of a species assemblage, which could be a local community, metacommunity, or a portion of a community.

Obviously, SD is defined based on SSs, and it aggregates (summarises) the *composition information* (entropy or uncertainty) of individual SS in the species assemblage. If we consider the difference in information, or the level of uncertainty, as a type of heterogeneity in species composition primarily resulting from species interactions, then Specificity Diversity (SD) essentially becomes a proxy metric of *compositional heterogeneity*. This concept of compositional heterogeneity is predominantly from a microbial perspective. However, strictly speaking, microbial compositional heterogeneity is not solely a product of microbial species interactions. The heterogeneity of host animals, which serve as habitats for the microbes, should also play a significant role.

The Specificity Diversity (SD) differs from traditional species diversity in three key aspects. First, SD is specific to the habitat (from the host animal perspective) or assemblage (from the microbial perspective), incorporating ‘information input’ from alternative habitats, whereas traditional species diversity is a property of the local assemblage (here we limit the concept of diversity to alpha diversity). Second, SD summarises compositional information (both species abundance and distribution), which is dependent on species identities, whilst species diversity synthesises species abundance information and is independent of species identities. Third, SD is strongly influenced by species interactions, taking into account both local species and species in the regional species pool from alternative habitats. In other words, SD can be considered a proxy for *compositional heterogeneity*. In summary, the differences between specificity diversity and species diversity lie in their ability to capture compositional and heterogeneity information, which is largely absent in the case of traditional species diversity.

The Species Specificity and Specificity Diversity (SSD) Framework is a term used to encompass a set of metrics/algorithms for conducting community/metacommunity specificity analyses. This includes three components: the previously introduced Species Specificity (SS), Specificity Diversity (SD), and a pair of statistical test methods based on the principle of the permutation test (Ma 2024a, 2024b) (see Figure 1, Box 1, and Box 2). The Specificity Permutation (SP) test is designed to assess the differences in each species' specificity across different habitats (between communities supported by different habitats). It can be used to generate lists (catalogues) of unique species (US), enriched species (ES), and/or no‐difference (ND) species in each community (habitat). A second statistical test, the Specificity Diversity Permutation (SDP) test, is designed to evaluate the overall difference between species assemblages from different habitat types in terms of their SD. If two assemblages are found to be different in SD, it indicates that both assemblages are compositionally different, not only in terms of species abundance, but also in terms of their distribution (prevalence). This is therefore a more comprehensive measure than standard diversity difference, which only considers abundance.

Additionally, the SD differences at various diversity orders (*q*) reveal compositional differences at different species specificity levels, equivalently from different scales of the generalist–specialist spectrum. Whilst differences at higher diversity order (*q*) levels emphasise the influences of specialist species, differences at lower diversity order (*q*) levels tend to highlight the influences of generalist species and/or rare species (Ma 2024a, 2024b).

Both the SP and SDP tests are performed with 1000 times of resampling. This means that the community samples from both treatments are randomly remixed 1000 times, and the differences from these 1000 iterations of resampling are compared with the observed actual difference to compute a pseudo‐*p*‐value. This value is used to determine the difference at a certain statistical significance level, specifically *p* = 0.05, which is adopted in this study. Furthermore, to minimise the false positive rates, i.e., the incorrect identification of differences in SS or SD when many comparisons are performed, we applied False Discovery Rate (FDR) control to the results from the SP/SDP tests with a significance level *p*‐value of 0.05. This ensures that our findings from the SSD are more conservative (reliable) in terms of declaring differences when simultaneously comparing many microbial or host species.

Box 2 outlines the computational algorithms and procedures for implementing the SSD framework. The corresponding R‐language code is provided as Supporting Information to this article, along with a brief help document and demonstrative input data.

### 2.3 Hierarchical (Cross Host Taxa and Diet Type) Analyses of the AGM With SDF

The 4903 AGM samples (containing 473,359 microbial species) were collected from 318 animal species of 10 major classes covering all vertebrates and four major classes of invertebrates. As illustrated in Figure 1, we apply the SSD framework at four scales, including (*i*) animal species level, (*ii*) animal class level—pairwise comparisons of 10 animal classes, (*iii*) phylum level—comparisons of invertebrates vs. vertebrates, and (*iv*) pairwise comparisons of three diet types (herbivores, carnivores, and omnivores). These analyses highlight the AGM difference from different host taxa and diet types, as demonstrated by their unique and enriched microbial species and the SD differences at the microbial community/metacommunity level, which can be considered as reflecting the manifestos of host phylogenetic histories and diet habits. Besides the SSD analyses, we establish the quantitative power‐law models (explained below) between phylogenetic (evolutionary) timeline and SD for each animal diet type, as introduced below.

### 2.4 Relationships Between AGM Specificity Diversity (SD) and Host Phylogenetic Timeline (PT)

Amongst the three components of the SSD, species specificity (SS) is assigned to each microbial species, and specificity diversity (SD) is computed for species assemblage, including the whole AGM of an animal species. To explore the relationships between phylogeny, diet type, and SSD, the SD is obviously the most appropriate choice given its one‐to‐one map to the PT (phylogenetic timeline) of animal host species. We introduce the following power‐law model to model the relationship between PT and SD, i.e., PTSD power‐law model:

(3) $$\mathrm{PT}=a{\mathrm{SD}}^{b}$$

which is equivalent with the following log–log linear model:

(4) $$\ln\left(\mathrm{PT}\right)=\ln\left(a\right)+b\;\ln\left(\mathrm{SD}\right)$$

where SD is specificity diversity, PT is phylogenetic timeline, *a* and *b* are fitted parameters.

BOX 2 **Summary of the SSD Framework: Key Computational Algorithms and Procedures.**

**Table:** 

Specificity permutation (SP) test algorithm for classifying species with species specificity	
Step [1]	Compute the species specificity (SS) for each species according to Equation (1), and construct two specificity lists for each treatment pair (one list for **A** & **B**, respectively). Further compute the actual (observed) specificity difference for each species between two treatments (**A** & **B**), and construct the corresponding actual (observed) specificity difference list for the treatment pair: Δ_*i*_ = |Δ_Ai_ − Δ_Bi_|, where *i* is species number.
*Step* [2]	Assume that there are *m* samples and *n* samples in **A** and **B**, respectively. Pool together the (*m* + *n*) samples, and permutate them randomly. From the randomly permutated samples, take the first *m* samples as a simulated treatment **A**(*k*), and the remaining *n* samples as a simulated treatment **B**(*k*), *k* for the *k*th simulation (repetition), where *k* = 1, 2, … 1000. Compute specificity lists for the simulated treatment pair and construct corresponding simulated specificity difference list: Δ_*i*_(*k*) = |Δ_*Ai*_(*k*) − Δ_*Bi*_(*k*)|, where *k* is the sequence number of *k*th repetition (simulation) as explained below in Step [3].
*Step* [3]	Repeat *step* [2] for 1000 times (*k* = 1, 2, …, 1000) and obtain the set of 1000 Δ_*i*_(*k*). Compare Δ_*i*_(*k*) from step [2] with Δ_*i*_ from step [1] for each species *i*, count the number of times (*T*) when Δ_*i*_(*k*) > Δ_*i*,_ and compute a pseudo‐*p*‐value, i.e., *p*_*i*_‐value = (*T*/1000) for each species *i*. If *p*_*i*_‐value ≤ 0.05, then there is significant difference between treatments **A** & **B** (i.e., the treatment effect is significant) in species *i*'s specificity. If *p*_*i*_‐value > 0.05, then the difference is not statistically significant and is caused by random effects. In other words, the proportion of events (*T*) or simulated differences exceeded observed differences is not a small probability event, and therefore, the random effects exceeded the treatment effects (no significant difference is observed).
*Step* [4]	Classify each species into one of the following five categories based on the pseudo‐*p*‐values associated with their specificity as well as their Δ_*i*_ from Step [1]. Unique species (US) in the treatment A, if *p*_*i*_‐value ≤ 0.05 and Δ_*Bi*_ = 0 & Δ_*Ai*_ > 0. Unique species (US) in the treatment B, if *p*_*i*_‐value ≤ 0.05 and Δ_*Ai*_ = 0 & Δ_*Bi*_ > 0 Enriched species (ES) in treatment A, if *p*_*i*_‐value ≤ 0.05 and Δ_*Bi*_ > 0 and Δ_*Ai*_ > Δ_*Bi*_ Enriched species (ES) in treatment B, if *p*_*i*_‐value ≤ 0.05 and Δ_*Ai*_ > 0 and Δ_*Bi*_ > Δ_*Ai*_ Non‐significantly different (Indifferent) species, if *p*_*i*_‐value > 0.05.
**Specificity Diversity Permutation (SDP) Test Algorithm for Detecting Assemblage Differences**	
*Step* [1]	Compute specificity diversity (SD) with Equation (2) for each of the six species categories classified with previous *SP algorithm*: Unique species (US) in the treatment A, or B; Enriched species (ES) in A or B; and two additional categories of ‘significantly different species’ and ‘all species’. Further compute the difference in their SD between treatment A and B: Δ=SDAq−SDBq, where Δ represents the *observed* (*actual*) absolute difference of SD.
*Step* [2]	Using the same Step [2] of the previous *SP test algorithm* to perform random permutations, compute the specificity diversity (SD) for each group of permutated A and permutated B treatments with Equations ((2), (3), (4)), and further compute their simulated absolute difference of SD (i.e., Δk=SDkAq−SDkBq) with *k* = 1, 2, …, 1000 repetitions (simulations).
*Step* [3]	Compute a pseudo *p*‐value for each of the six categories by comparing the two Δs from Step (1) and Step (2). The pseudo *p*‐value is the proportion of the permutations with $\Delta \left(k\right)$ > Δ. That is, assuming *T* is the number of times satisfying $\Delta \left(k\right)$ > Δ amongst 1000 permutations, *p‐*value = *T*/1000. If *p‐*value ≤ 0.05, there are significant differences in the SD between treatments A & B; otherwise, the difference is insignificant statistically. Similar to the previous SP test, when *p*‐value > 0.05, the proportion of events (*T*) or simulated differences exceeded observed differences is not a small probability event, and therefore, the random effects exceeded the treatment effects (no significant difference is observed).

## 3 Results

### 3.1 Unique/Enriched Species (US/ES) of AGM of Different Animal Taxa and Diet Types

We use the term ‘habitat’ to refer to host animals of the AGMs (strictly, animal gastrointestinal tract), either to mean different animal taxa or different diet types (herbivores, carnivores, and omnivores). A hallmark of the SSD framework is the identification of unique species (US), enriched species (ES), and non‐significantly different species (NS) in each habitat, which could be host animal *species*, *class*, *phylum*, or *diet* types. Accordingly, from the host animal perspective, we adopted four scales of comparisons (a total of six comparisons) as follows: (*i*) host animal species vs. species, (*ii*) class vs. class, (*iii*) invertebrates vs. vertebrates, and (*iv–vi*) pairwise comparisons of three diet types (herbivores, carnivores, and omnivores).

**FIGURE 2:**
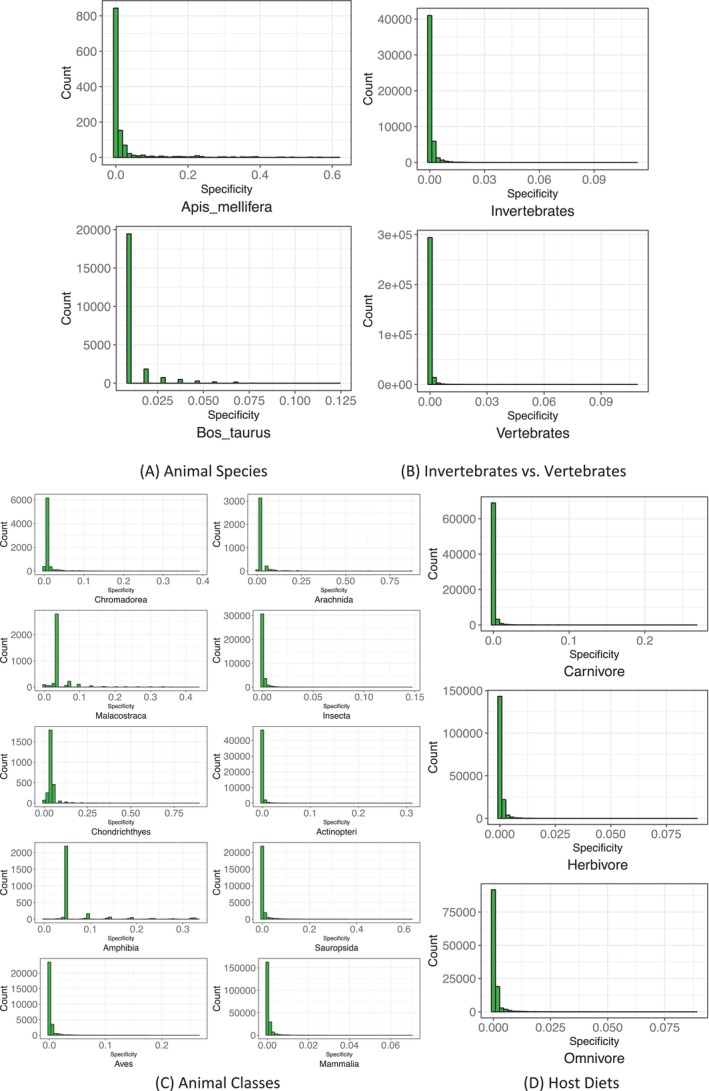
Species specificity distributions (represented in histograms) are highly left‐skewed: (A) species level comparison between two representative species; (B) phylum level comparison of invertebrates vs. vertebrates; (C) ten animal classes; (D) three diet types.

Figure 2 illustrates the distribution (in the form of a histogram) of species specificity of the AGMs, consisting of the four graphs (Figure 2A–D) corresponding to the AGM specificity histogram of (*i*) a pair of animal species ( *Apis mellifera* vs. *Bos taurus* ), which were chosen to observe the potential extremes of the differences, (*ii*) all samples of invertebrates vs. all samples of vertebrates, (*iii*) 10 animal classes, and (*iv*) 3 diet types. An intuitive and common property of these AGM specificity distributions is that they are highly skewed, similar to the species abundance distribution in community ecology. In addition, Table S2 (in MS‐Excel Sheets) show the species specificity values of the top 100 species with the highest specificity, corresponding to the previously defined animal taxa and diet types. Table S2 list the microbial phyla and classes to which the top 100 microbial species (listed in Table S2) belong.

**FIGURE 3:**
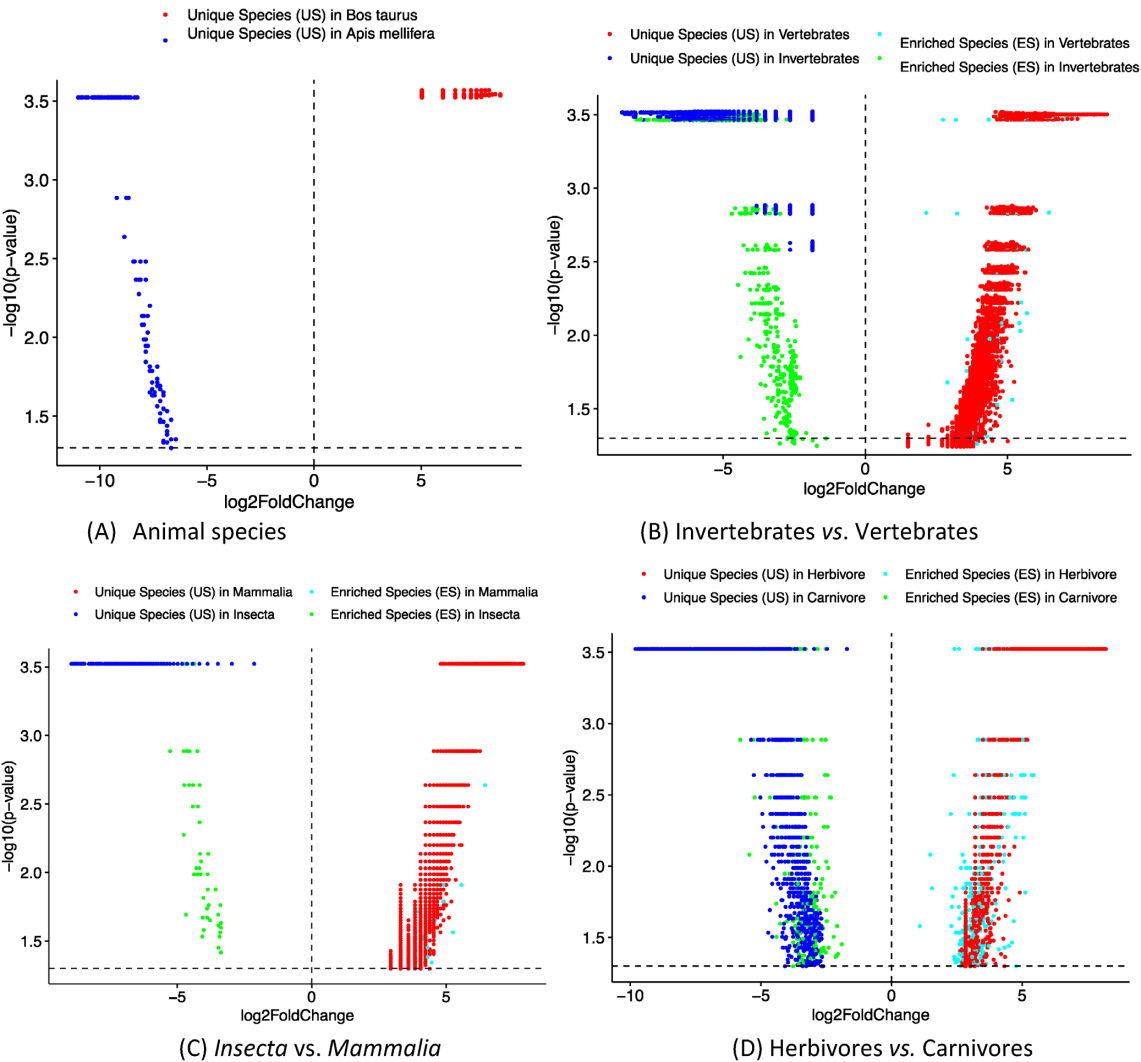
The volcano maps show the distribution of unique species (US), enriched species (ES) in each treatment of the comparison pair (both belong to the category of significantly different species in species specificity) and the insignificantly different species, generated by specificity permutation (SP) tests. The *X*‐axis represents the log‐transformation of the specificity fold change between two treatments of the comparison pair, where fold change = *SS*(former treatment)/*SS*(latter treatment) (*SS* represents specificity of species); *Y*‐axis represents the negative log‐transformation of the *p*‐value from SP tests of the specificity differences between the former and latter treatments. The vertical dotted line at *X* = 0 represents fold change = 1 [i.e., *SS*(former) = S(latter)], the points in the right side of this dotted line represent species with *SS*(former)/*SS*(latter) > 1 [i.e., *SS*(former) > *SS*(latter)], the left points represents species with *SS*(former)/*SS*(latter) < 1 [i.e., *SS*(former) < *SS*(latter)]. The horizontal dotted line represents *p*‐value = 0.05 [−log10*(0.05) = 1.301], the points above the line represent species specificity with significant differences between the former and latter, and the points below represent species of non‐significant differences in specificity. Therefore, the grey points represent species of non‐significant differences in species specificity between both treatments, cyan points represent significant enriched species in the former, green points represent significant enriched species in latter, red and blue points represent unique species in former and latter, respectively. Note, to reduce the file size of the volcano maps, the points representing for ‘insignificantly different species (IDS)’, which should be located at the bottom section of the Cartesian coordinate system, were omitted in the graphs. Note that adaptations to standard volcano maps have been made to include all relevant points in their respective regions of the Cartesian coordinate system. This adaptation does not affect the validity of the results. However, for precise results, readers are referred to the corresponding Supporting Information tables used to draw the volcano maps.

For the above described AGM species specificity lists for various animal taxa and diet types, the specificity permutation (SP) tests were performed to determine and catalogues various species categories, including US (unique species) and ES (enriched species) in each ‘habitat’ (taxon or diet type) of the comparison pair, and a fifth category of species without significant differences in species specificity (SS) or insignificantly different species (IS). The first four categories, i.e., 2 USs and 2 ESs in both habitats of the comparison, have significant differences in SS. Figure 3 illustrates the five species categories generated by the SP tests with volcano maps. An advantage of the volcano map is that it can show the distributions of the five species categories (2 USs, 2 ESs, IS) with scatter points in four quadrats of the coordination system, based on the magnitude of the differences in SS on the log scale. Table S1C–J shows the catalogues (lists) of the various species categories illustrated in Figure 3.

**FIGURE 4:**
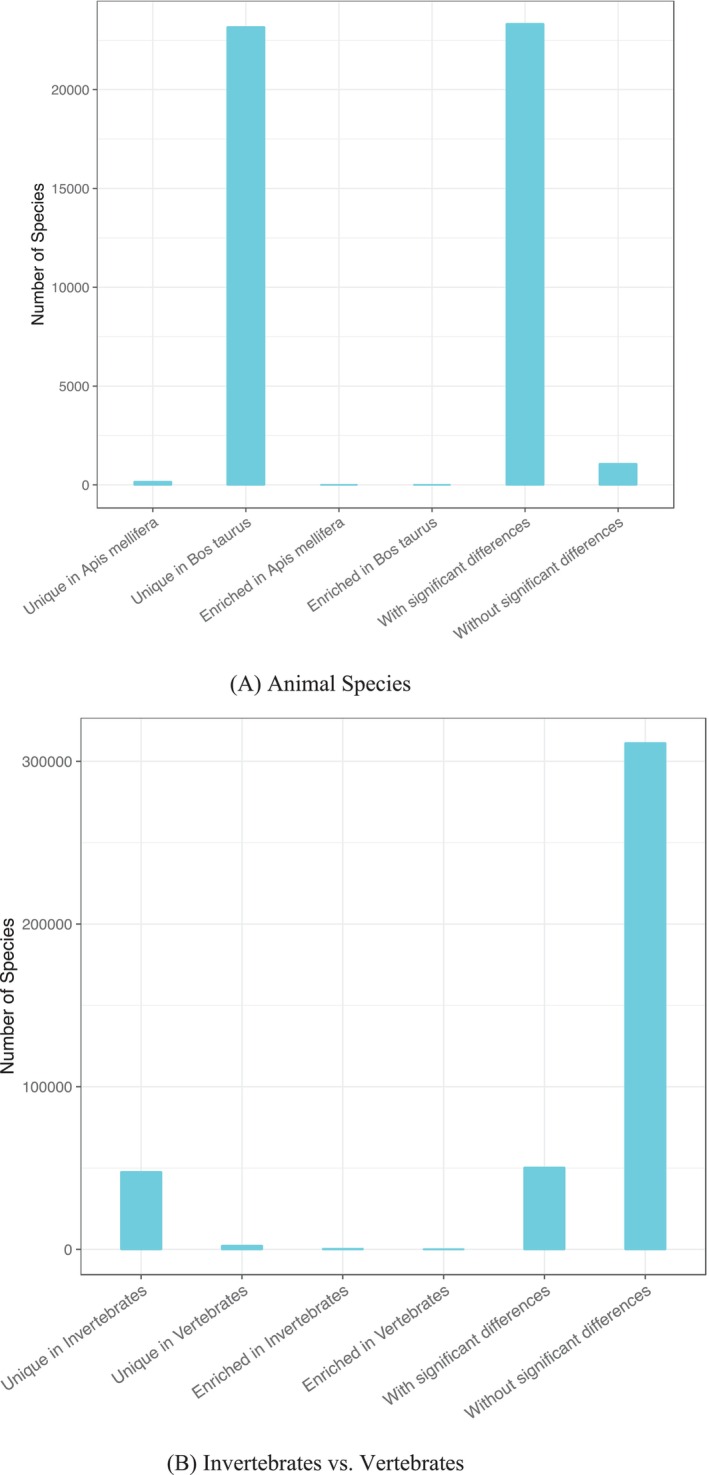
Species numbers (richness) of different species categories classified with specificity permutation tests, drawn based on Tables S1A and S9.

Figure 4 further shows the numbers of microbial species in each species category (*See* Table S1A for detailed information and Table S9 for the summary information), namely species richness of each species category, generated from the previously defined six pairwise comparisons. Figure 4A exhibits the species richness values of the six species categories of pairwise comparison of two animal species ( *Apis mellifera* vs. *Bos taurus* ): besides the five species categories previously mentioned, i.e., 2 USs, 2 ESs, and IS, a fifth category (with significant differences in species specificity) sums up the four categories of significant differences (i.e., 2 USs and 2 ESs). Similarly, Figure 4B illustrates the microbial species richness comparison between invertebrates and vertebrates. Table S9 shows the averages of species richness from pairwise comparisons of 10 animal classes and the averages from comparing three diet types.

### 3.2 Compositional Heterogeneity of AGMs in Terms of Specificity Diversity (SD) Between Animal Taxa or Diet Types

The previous results were generated from the computation of microbial species specificity (SS) and SS permutation (SSP) tests. The results reported in this section were generated from the computation of specificity diversity (SD) and SD permutation (SDP) tests. The computation/test scheme is the same as the one previously used for SSP tests, i.e., host animal species, class, phylum (invertebrates vs. vertebrates), and diet types. Besides that, the comparisons are also performed at the microbial species category (US, ES, IS) scales within each taxon/diet level comparison.

**FIGURE 5:**
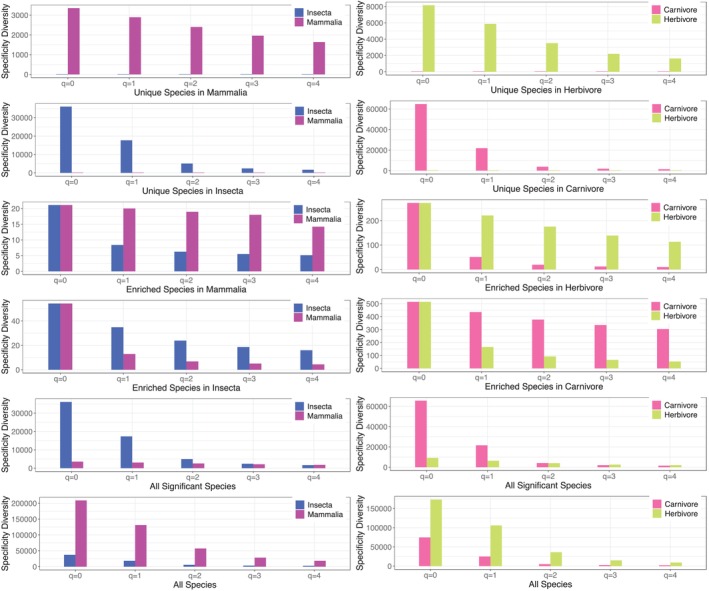
Specificity diversity (SD) (*Y*‐axis) of different species categories (assemblages) at different diversity orders (*q* = 0–4): Left—from comparison of *Insecta* vs. *Mammalia* classes; Right—from comparison of herbivores vs. carnivores.

Figure 5 illustrates two examples of the SDP tests, the comparison between *Insecta* and *Mammalia* classes, and between carnivores and herbivores. For example, in the comparison of *Insecta* and *Mammalia* (Figure 5A), the SD of six species categories including ES in Insecta, ES in Mammalia, US in Insecta, US in Mammalia, species with significant differences in SS, and all species without considering the difference in SS. The *Y*‐axis shows the SD, whilst the *X*‐axis shows the SD order *q* = 0–4. All of the *p*‐values from the SDP test results are listed in Tables S4–S6 for various test schemes introduced above.

The SDP test results displayed in Tables S4–S6 revealed that for the species categories of US and ES, in particular, the US category, the SD values are generally significantly different (*p*‐value < 0.05) between virtually all animal taxa and/or diet types. That is, each host animal taxon, or diet type, from species to phylum, seems to possess specific specificity diversity. Compared with traditional species diversity (Ma et al. 2022), the differences in specificity diversity are more prevalent than species (abundance) diversity. This should be expected because specificity, from which specificity diversity is defined and computed, is a synthesis of both abundance and distribution (prevalence), whilst traditional species diversity is based on species abundance only.

A general trend shown by Tables S4–S6 is that the significance levels (magnitudes) of the SD differences of various species categories between different taxa or between diet types are in the following orders: US > ES > ‘all significantly different species’ > all species (ignoring the differences in specificity). In terms of the proportions with differences in SDP tests, the levels for US, ES, all significantly different species, and all species are in the *approximate* ranges of [2/3 to 1], [1/2 to 1], [1/2, 2/3], and [1/10, 1/3], respectively. That is, for the comparisons between animal taxa (or diet types), the likelihood (proportions) with significant differences in the SD for the species categories exceeds ½ in general, as long as their species specificity is significantly different. In other words, species‐level differences in species specificity often, but not always, lead to holistic differences in specificity diversity. In the extreme (the last species category), when specificity differences are not in consideration, the holistic differences in SD are usually below 1/3. This extreme case, when specificity is ignored, is generally similar to the case of traditional species diversity as performed in Ma et al. (2022), which should be expected, as explained previously, for the reason that specificity and specificity diversity are synthetic metrics of distribution and abundance, whilst traditional species diversity only considers abundances. When the specificity of species is ignored, both species diversity and specificity diversity should display similar patterns in comparing AGMs.

Specificity diversity order (*q =* 0–4) determines the nonlinearity levels of the weighing scheme of the Hill numbers (i.e., Renyi's entropy that is used to measure specificity diversity). When *q* = 0, the specificity does not weigh in, and the specificity diversity defaults to traditional species richness in species diversity. For this reason, the SDP test for *q* = 0 is trivial for species categories of US and ES because, for US species richness in one treatment (taxon) of the comparison pair must be zero and for ES, it must be equal in both treatments. When *q* = 1, the Hill numbers are weighted in proportion to the relative species specificity, emphasising the contribution of typical species in species assemblage. When *q* > 1, the weighing scheme favours high specificity species, which tend to be specialist species in local species assemblage.

### 3.3 PTSD (Phylogenetic‐Timeline—Specificity‐Diversity) Modelling for Each Diet Types

We conjecture that the AGM specificity diversity (SD) of an animal species should be related to the phylogenetic timeline (PT) and diet types in some nonlinear patterns. As introduced in the materials and methods section, our modelling strategy is, for each animal diet type, to build a PTSD (phylogenetic‐timeline—specificity‐diversity) model. Table S7 lists the PTSD model parameters in the form of a power law model for carnivores, herbivores, omnivores, and all species without considering diet types (the combined). It turned out that the PTSD power‐law models are significant (*p*‐value < 0.05) for the herbivores and the whole animal kingdom (ignoring diet types), whilst the models for carnivores and omnivores are statistically insignificant. Furthermore, all scaling exponent parameters of the power law models are negative, which suggests that ancient host animal species with smaller ‘evolutionary age’ (phylogenetic timeline) have relatively lower AGM specificity diversity than modern animal species. Since specificity diversity is essentially entropy, high entropy values imply higher complexity (higher level of disorder or uncertainty), and low entropy values imply lower complexity. So it appears that the evolution of host animal species tends to raise the specificity diversity of their gastrointestinal microbiomes, equivalently increasing the complexity of AGM distribution and compositions.

**FIGURE 6:**
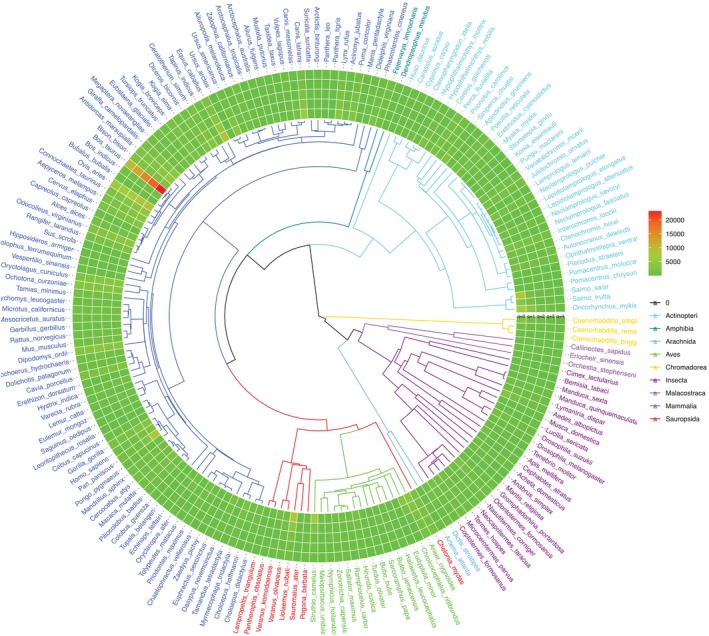
The phylogenetic tree (P‐Tree) of 183 animal species, annotated with their gastrointestinal tract microbiome specificity diversities: (i) Branches and species labels constitute a standard phylogenetic tree and were coloured differently for each of the 10 animal classes (species labels were coloured in terms of their class identities); (ii) The four bands constitute the heatmap of gut microbiome specificity diversities (in the Hill numbers at diversity order *q* = 0–4) of the animal species.

**FIGURE 7:**
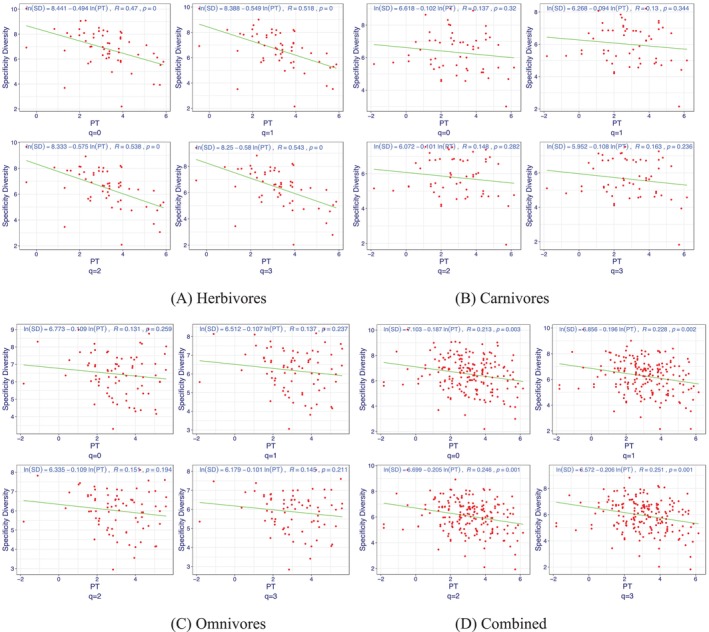
PTSD (phylogenetic‐timeline specificity‐diversity relationship) models for herbivores, carnivores, omnivores, and the combination of three diet types, respectively. The PTSD models for herbivores and the combination are statistically significant (*p*‐value < 0.05), and those for carnivores and omnivores are not.

Figure 6 illustrates the phylogenetic tree of 183 animal species out of 308 species, for which the PD data are available, and the tips of the tree branches are connected with the heatmap of specificity diversity at different diversity orders (*q* = 0–4). Figure 7 further illustrates the fitting of the PTSD on the log scale, for three diet types and the combination of the three diet types, respectively. The negative correlations on log scale between PT and SD are obvious in Figure 7.

## 4 Conclusions and Discussion

### 4.1 Conclusions

The present study is aimed at investigating the distributions and abundances of animal gastrointestinal microbiomes (AGMs) and their relationships with host animal phylogeny and diet types by leveraging the SSD framework (SDF) methodologically (Ma 2022) and big AGM datasets of 318 animal host species and 473,359 microbial species, covering all six vertebrate and four major invertebrate classes. The phylogeny information is represented implicitly by taxonomic ranks or taxa (species, class, etc.) and explicitly by phylogenetic timeline (PT). The diet type information is simply represented by a categorical variable, i.e., carnivores, herbivores, and omnivores. Therefore, the SSD analysis grouped by taxa and diet types implicitly reveals the influences of phylogeny and diet types on the distribution (prevalence) and abundance of AGMs, i.e., the influences of different microbial habitats (different taxa and diet types).

### 4.2 Discussion

Conceptually, this study diverges from existing similar research due to its adoption of the concepts of Species Specificity (SS) and Specificity Diversity (SD). These concepts synthesise both species abundance and distribution (prevalence) information across microbial communities/metacommunities (gastrointestinal microbiomes) and habitats (such as different species or classes of host animals). Therefore, SS and SD significantly differ from traditional species abundance and species diversity, even though both SD and species diversity utilise the same Renyi entropy formula (Hill numbers) to summarise species specificity and species abundance, respectively. Furthermore, a fundamental difference from species diversity is that SD can be considered a proxy for compositional heterogeneity, as previously argued. The difference between SS and species abundance is even more pronounced—SS synthesises both abundance and distribution, whilst species abundance is simply that—species abundance.

The other components of the SSD, including specificity permutation (SP) tests, specificity diversity (SD), specificity diversity permutation (SDP) tests, enable one to obtain US (unique species), ES (enriched species), and further test the holistic differences in SD of species categories (such as US and ES) between different habitats. The habitats refer to either host animal taxa (phylogeny information is implicitly built in) or diet types. We designed four kinds of animal taxon comparisons: species level, class (10 classes), phylum (invertebrates vs. vertebrates), and 3 kinds of pairwise diet comparisons. For each of those comparisons, the US/ES catalogues are detected and compiled with statistical rigour. Furthermore, holistic differences in SD from those comparisons are tested with statistical rigour.

Whilst those comparative results from SSD analyses implicitly reveal the influences of host phylogeny and diet types on the distributions and abundances of AGM, we further constructed PTSD (phylogenetic‐timeline–specificity diversity) power‐law models for each diet type and all diet types combined. It turned out that the PTSD power‐law models are significant for herbivores and all‐diet‐types combined, and the correlation relationships (scaling parameters) are negative, which suggests that evolution leads to a more complex AGM structure (distributions and abundances), as suggested by higher SD for more modern host animal species or lower SD for more ancient species. The power‐law form of the PTSD also explains the highly skewed distribution of species specificity observed in Figure 2.

Strictly, it is hardly appropriate to *directly* compare our results with existing extensive studies on microbial species diversity, phylogeny, diet types (Ley et al. 2008; Muegge et al. 2011, Delsuc et al. 2014; Vital et al. 2015; Degnan et al. 2012; Carmody et al. 2015; Vital et al. 2015, Bik et al. 2016; Martinson et al. 2017; Moeller et al. 2014, 2016, 2017; Groussin et al. 2017; Gaulke et al. 2018; Sherrill‐Mix et al. 2018, Mazel et al. 2018, Näpflin and Schmid‐Hempel 2018, Campo et al. 2019, Amato et al. 2019, Gomez et al. 2019, Youngblut et al. 2019, Lavrinienko and Tukalenko 2019, Song et al. 2020, Trevelline et al. 2020, Roja et al. 2021, Ma 2021, Ma 2022, Ma 2024a, Ma and Ellison 2024) because of the reasons discussed previously, particularly that distribution (prevalence) is explicitly measured, integrated with abundance, in our SSD framework. For example, in existing studies, it was hardly feasible to systematically identify US/ES species with statistical rigour (*p*‐values), which made existing studies only able to deal with holistic community properties (i.e., microbial biodiversity measures) and could not offer much meaningful information on species *compositions* (US/ES, etc.), not to mention their distribution (prevalence) across habitats (especially at larger taxon scales). In terms of the relatively new term, ‘compositional heterogeneity,’ which we use cautiously in this article due to its complexity and untested nature elsewhere, the results generated from the application of the SSD Framework bring us one step closer to heterogeneity research, thereby deviating from traditional diversity research.

As we approach the conclusion of this study, we would like to highlight another potential avenue for advancing AGM research: the complex network analysis of the AGM based on the SSD Framework. This approach could potentially differ from traditional network approaches that are based on species abundances (Ma and Ellison 2019; Ma 2020a; Ma and Shi 2024). Given the preliminary nature of the ‘specificity’ network analysis, we have chosen to present the results in this discussion section, rather than in the previous results section.

**FIGURE 8:**
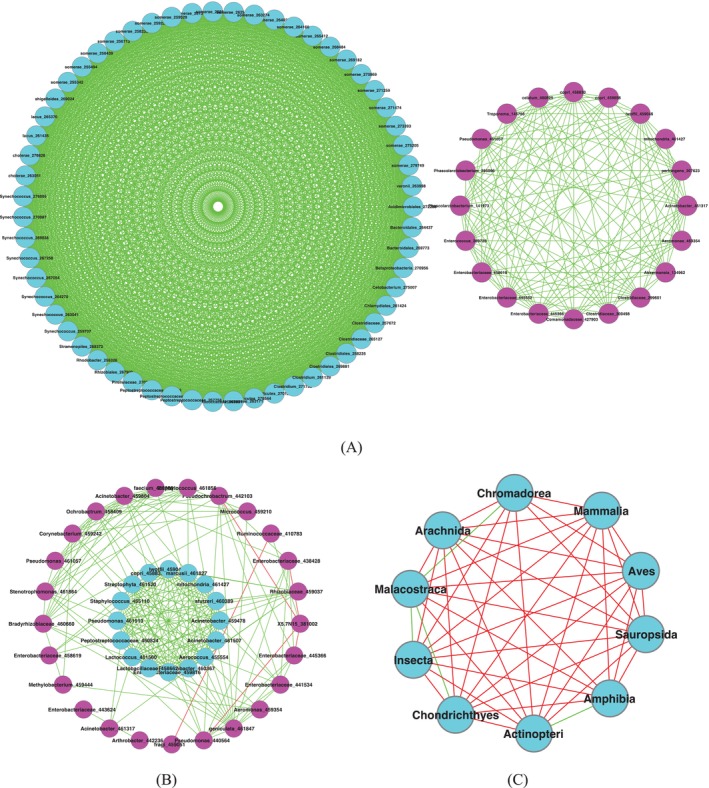
The species specificity network: (A) Microbial species specificity network at host animal species level (the left in cyan colour is the core and right in magenta is the periphery); (B) microbial species specificity network at host animal class level (the centre is the core and the outer circle is the periphery); (C) class specificity network with host animal classes as nodes based on the correlations in microbial species specificity.

Figure 8A displayed the AGM network built at the host animal species level in the form of core/periphery network structures. Figure 8B is similarly built as Figure 8A except that the host animal taxon level is class, rather than species. Figure 8C is built differently from the previous two networks, with host animal classes as nodes of the network (10 classes or 10 network nodes), with edges constructed based on the correlations between animal classes in their microbial species specificity.

To build these specificity networks, the species specificity values for 10 classes and 301 animal species, respectively, and a species specificity matrix is obtained with rows representing OTUs, columns representing host animal classes, and matrix elements representing the OTU specificity at specific habitat (class). To build the microbial species specificity network at the animal species level (Figure 8A), we filtered out the OTUs that occurred in less than 5% of animal species to compute Spearman's correlation coefficients, and the coefficients were further filtered with FDR (false discovery rate) control at a *p*‐value = 0.05. To build the species specificity network at the animal class level (Figure 8B), we filtered out the OTSs that occurred in less than 6 animal classes, and the other computational procedures (computation of correlation coefficients and FDR control) were the same as those used in building Figure 8A. To build the class specificity network with animal classes as 10 nodes (Figure 8C), the same species specificity matrix, previously discussed, is used with FDR control of a *p*‐value = 0.05. That is, the nodes are animal classes, and edges are constructed based on the correlations between classes in their microbial specificity values in the specificity matrix.

The message from ‘microbial species specificity network at host animal species level’ (Figure 8A) is that the core (the left circle with nodes in cyan colour) is densely connected and the periphery (the right circle with nodes in magenta) is relatively sparsely connected, and the core and periphery are virtually disconnected. We say they are ‘virtually’ disconnected because when the threshold for including network nodes (microbial species) is lowered to occurring in 3% of animal species, they are connected although sparsely. In addition, all network links are positive (green links), which suggests that species specificity tends to aggregate rather than segregate at host species level.

The message from microbial species specificity network at host animal class level (Figure 8B) is that the core (the inner circle with nodes in cyan) is densely connected and the periphery (the outer circle with nodes in magenta) is relatively sparsely connected, and the core and periphery are sparsely connected, which is different from its counterpart network at animal species level. The PN ratio (the ratio of positive to negative links) for this network at animal class level is 37.8, which indicates that there are a small number of negative links (red links) in this network, rather than zero as in the previous species‐level network in which the PN ratio was infinity. Nevertheless, the network is still predominantly positively correlated, which suggests that the microbial species are in general aggregated rather than segregated in animal gastrointestinal habitats at the host species level.

The message from ‘class specificity network with host classes as network nodes’ (Fig C) is that most nodes (classes) are negatively correlated with their microbial species specificity, and only 4 links are positive. The predominantly negative links suggest that the microbes are generally segregated amongst host animal classes—each animal class may have their class‐specific microbes. This pattern is contrastingly different from host animal species level pattern, where microbial species aggregate at the host animal species scale. We caution that this apparently contrasting difference between host species and class in microbial distribution needs further investigation in future.

Finally, we would like to underscore the potential practical significance of studying animal microbiomes, particularly in comparison with human microbiomes. In a previous study (Ma 2021), we examined the underlying mechanisms for community assembly and diversity maintenance in both the AGM (using the same datasets as this article) and human gut microbiomes. Just as the study of human microbiomes is crucial to understanding human health and diseases (e.g., Ma and Li 2019; Ma et al. 2019; Ma and Ellison 2019; Ma 2020a, 2020b, 2020c), the study of AGM is equally important for understanding the disease ecology of wildlife. Indeed, microbiome research has become a critical component in unifying all medical enterprises, including the medical ecology of humans and the disease ecology of animals (Ma and Zhang 2022).

Investigations on AGM can also be of critical significance for wildlife conservation and protection of endangered species. Some researchers and practitioners in the related fields have already been advocating the protection of holobionts (e.g., Carthey et al. 2019), rather than protecting animals alone as in traditional conservation biology. If the idea or paradigm of ‘holobiont conservation’ should be pursued, then the protection of the microbiome part of the holobiont should be equally, if not more, important than the protection of the host animal per se. In consideration of this potential paradigmatic shift in conservation biology, we detected and compiled a catalogue of exclusively unique species (EUS) in each of the 10 major animal classes in Table S1B and listed the EUS numbers in Table S10. This catalogue is different from previously analysed and discussed unique species (US) in previous sections (Tables S1A and S9), given the EUS (Table S10) is based on exclusive‐or (XOR) set operations across the whole animal kingdom and therefore exists only in a specific class. Obviously, this catalogue is of strategic significance for holobiont conservation because it reveals what are exclusively unique microbial species for an animal class, which most likely should receive prioritised attention. It turns out that the catalogue of EUS is rather small, comprising only 252 microbial species, with 100 of these being unique to amphibians (Table S10). Considering that these 252 EUS were identified from the 473,359 AGM species analysed in this study, the minuscule nature of the EUS is somewhat surprising, if not shocking. This suggests that animals may not be as different as we think in terms of their gastrointestinal microbiomes. Of course, similar phenomena are abundant in evolutionary biology. The surprising similarity between the human genome and that of primate mammals may even overshadow the similarity we uncover here. Perhaps, high similarity should be the norm, whether we're talking about the genome, metagenome, or hologenome. This could be a key takeaway from this study and certainly warrants further investigation. Perhaps the fact that amphibians host nearly 40% (100 out of 252) of the Enriched Unique Species (EUS) serves as another striking demonstration of their unique position in the evolution of the animal kingdom.

### 4.3 Limitations and Future Research

We thank an anonymous expert reviewer for identifying a critical limitation concerning the ecological plausibility of certain high‐specificity bacterial taxa, such as the assignment of strict anaerobes (e.g., *Ruminococcus*, *Prevotella*) to *Drosophila* or soil‐associated *Rhizobiales* to *Cimex*. These unexpected assignments highlight that our datasets likely included signals not representing true, stable host‐symbiont relationships. Following this insight, we investigated three potential sources.

First, regarding computational rigour, our SSD framework employs deterministic rules and permutation tests to filter noise. Whilst it performs robustly on other complex data types (e.g., SNPs, single‐cell genomics), its efficacy remains constrained by input data quality. Second, artificial laboratory contamination is a major plausible factor. Addressing this would require fine‐tuning the bioinformatics pipeline (QIIME2) we used to compute the OTU tables that were fed into our SSD framework, but curating a universally optimal parameter set for our meta‐dataset of 108 heterogeneous studies presents a substantial, unresolved challenge. Finally, distinguishing natural transient passengers from core symbionts remains a fundamental methodological frontier in the field. Therefore, whilst Table S2A serves as a valuable resource, we caution users—particularly for non‐mammalian hosts—to interpret entries with care. Future work should prioritise developing scalable, standardised filtering protocols for large meta‐datasets to more reliably differentiate symbiotic signals from artefacts.

We are also grateful to the expert reviewer for inspiring us to more explicitly contextualise our approach to host‐specificity within established ecological and evolutionary frameworks, such as phylosymbiosis (e.g., Brooks et al. 2016) and cophylogeny (e.g., Moeller et al. 2016). Integrating our findings into this ongoing discourse and situating them within the current, nuanced understanding of these frameworks is an important objective for future research.

The quest to understand the drivers of host‐specificity in animal microbiomes has been profoundly shaped by two complementary approaches: one focusing on community‐wide assembly patterns and the other on the evolutionary history of individual microbial lineages. Whilst the initial concepts of phylosymbiosis and bacterial cophylogeny were foundational, they have since been critically examined and refined, evolving from descriptive patterns towards more mechanistic and probabilistic models.

The phylosymbiosis framework, established by Brooks et al. (2016), posits that the evolutionary relationships between hosts are recapitulated in the ecological relationships of their microbial communities. This was a paradigm shift, suggesting that host phylogeny acts as a deep‐time filter for the microbiome, resulting in a measurable signal of host‐specificity at the entire community level. However, a primary critique emerged that this pattern could be an epiphenomenon of shared ecology and diet amongst closely related hosts, rather than a result of host‐genetic drivers (Moran and Sloan 2015).

In parallel, the cophylogeny framework, powerfully illustrated by Moeller et al. (2016) in hominids, revealed an even deeper layer of host‐specificity: the parallel diversification of host and microbe. Their finding that specific bacterial lineages in the gut had cospeciated with their hosts over millions of years pointed to an extreme form of specialisation, implying long‐term vertical transmission and co‐adaptation.

Within this refined conceptual landscape, the SSD framework we demonstrated can help integrate community‐level and taxon‐level analyses of host‐specificity. Traditional approaches often analyse these scales separately: one focusing on the overall community pattern (phylosymbiosis) and the other on the evolutionary history of specific bacterial taxa (cophylogeny). Our SSD offers a complementary methodology that bridges this scale of analysis.

Specifically, the SSD operates at two interconnected levels: (i) At the microbial taxon level, the Species Specificity (SS) metric quantifies the degree to which a given microbial group (e.g., an OTU) is restricted to a particular host group. This allows for the systematic identification of specialist and generalist taxa without requiring their phylogenetic trees. (ii) At the metacommunity level, the Specificity Diversity (SD) metric integrates the SS of all constituent taxa to quantify the overall distinctness or heterogeneity of a host's microbial assemblage. This provides a direct, quantitative measure of the community‐level patterns that concepts like phylosymbiosis seek to describe. Furthermore, the PTSD power‐law relationship between Specificity Diversity (SD) and the host Phylogenetic Timeline (PT) offers a new way to quantify how community‐level compositional heterogeneity scales with host evolutionary age. Whilst this model does not directly test the mechanisms of co‐diversification, it provides a correlative pattern that could be consistent with emerging concepts of how phylosymbiosis arises from complex host–microbe interactions.

In perspective, our SSD framework does not replace existing methods but addresses a specific gap by providing unified metrics to quantify host specificity at both taxon and community levels. Applying this approach across the animal kingdom offers a broad‐scale tool to complement established frameworks like phylosymbiosis and cophylogeny. Future work integrating experimental and computational validation will be essential to fully reconcile our findings with these foundational theories.

## Author Contributions

Zhanshan (Sam) Ma designed and performed the study, wrote, and revised the manuscript.

## Funding

This work was supported by Bullard Fellowship from Harvard University, National Natural Science Foundation of China, 72274192, and Prosperous Yunnan Talent Support Program.

## Ethics Statement

N/A, since the study does not involve any wet‐lab experiments or surveys on human or animal subjects, and all analysed datasets are already available in the public domain, as mentioned above.

## Conflicts of Interest

The author declares no conflicts of interest.

## Supporting information


**Data S1.** Supplementary Information.

**Data S2.** Tables S1B–S2D‐FDR‐V3‐Final.

**Data S3.** SSD_code_AGM.

## Data Availability

This study is a reanalysis of previously published raw sequencing data. The operational taxonomic unit (OTU) tables were recomputed from the original raw reads and can be obtained from the corresponding author for legitimate research purposes. The custom computational R code, help documentation, and demonstrative datasets developed for the analyses are available as part of the online supplementary information for this article. Comprehensive metadata pertaining to the source studies and the derived OTUs are documented in the following resources: Table S1 of Ma (2021) *mSystems* 6: e00633‐21. https://journals.asm.org/doi/10.1128/mSystems.00633‐21. Table S7 of Ma (2022) *FEMS Microbiology Ecology* 98(2), fiac021. https://academic.oup.com/femsec/article/98/2/fiac021/6534254. The Harvard Forest Data Archive (dataset HF448). https://harvardforest1.fas.harvard.edu/exist/apps/datasets/showData.html?id=HF448. Table S8 of this article.
