# Supplementary material for: How Host Phylogeny and Diet Shape the Specificity and Specificity Diversity of Animal Gut Microbiomes
Source: Environ Microbiol Rep. 2026 Jan 29;18(1):e70253. doi: 10.1111/1758-2229.70253 (PMC12856063; doi:10.1111/1758-2229.70253)
Supplement: Supplementary file 1 — Data S1. Supplementary Information. [file EMI4-18-e70253-s003.pdf]

Online Supplementary Information for: Ma ZS (2024) How Host Phylogeny and Diet Shape the Specificity and Specificity Diversity of Animal Gut Microbiomes

Design based on Host Animal Phylogeny and Diet Types

1. Species Level: Comparison between *Apis mellifera* vs. *Bos taurus* as an example
2. Phylum Level: Invertebrates vs. Vertebrates

3A. Class Level: 45 possible pairwise-class comparisons between 10 host animal classes
3B. Class Level: 9 sequential comparisons along the phylogenetic timeline, subset of 2A

4. Diets Level: Pairwise comparisons between Carnivore, Herbivore and Omnivore

Lists of Online Supplementary Tables

Species numbers (richness) of each species assemblages (groups, categories)

Table S1A. The number of AGM species in each species category, classified based on specificity permutation (SP) test with FDR (false discovery rate control) corrections at *P*-value=0.05

Table S1B. The exclusively unique species (EUS) in each animal class, *i.e., do not exist in any other 9 animal classes.* However, four classes including *Insecta* and *Mammalia* do not have any EUS.

Table S1B-S1J Excel Sheets

Table S1C. The catalogues of species categories from comparing *Apis mellifera* and *Bos taurus*

Table S1D. The catalogues of species categories from comparing between Invertebrates and Vertebrates

Table S1E. The catalogues of species categories from comparing *Insecta* and *Amphibia* classes

Table S1F. The catalogues of species categories from comparing between Insecta and Mammalia Classes

Table S1G. The catalogues of species categories from comparing between Amphibia and Mammalia

Table S1H. The catalogues of species categories from comparing between Carnivore and Herbivore

Table S1I. The catalogues of species categories from comparing between Carnivore and Omnivore

Table S1J. The catalogues of species categories from comparing between Herbivore and Omnivore

Top 100 species on the species specificity lists from the comparisons at species, class, phylum and diet type levels

Table 2A-2D are in separate MS-Excel sheets

Table S2A. The top 100 (sorted by species specificity decreasingly) microbial species for each host animal species

Table S2B. The top (sorted by specificity) 100 microbial species for Invertebrates and Vertebrates, respectively

Table S2C. The top 100 (sorted by species specificity decreasingly) microbial species for each of the 10 host animal classes

Table S2D. The top 100 species (sorted by species specificity) for each diet type (Carnivore, Herbivore and Omnivore)

Table S2E. The catalogues of microbial phyla and classes of top 100 microbial species with the highest specificity from comparing *Apis mellifera* vs. *Bos taurus* (the numbers inside the parentheses represent the number of species belonging to that taxon).

Table S2F. The catalogues of microbial phyla and classes of top 100 microbial species with the highest specificity from pairwise comparisons between 10 animal classes (the numbers inside the parentheses represent the number of microbial species belonging to that phylum or class).

Table S2G. The catalogues of microbial phyla and classes of top 100 microbial species with the highest specificity from pairwise comparisons of the three diet types (the numbers inside the parentheses represent the number of microbial species belonging to that phylum or class).

Table S2H. The catalogues of microbial phyla and classes of top 100 microbial species with the highest specificity from comparing invertebrates and vertebrates (the numbers inside the parentheses represent the number of microbial species belonging to that phylum or class.

SDP (specificity diversity permutation) tests at host species level: from comparing two representative species (*Apis mellifera* and *Bos taurus*)

Table S3. Permutation tests for the *specificity diversity* (SD) from comparison between *Apis mellifera* and *Bos taurus*

SDP (specificity diversity permutation) tests for the SD (specificity diversity) from comparing between invertebrates and vertebrates

Table S4. SDP (Specificity diversity permutation) tests of the SD from comparing between invertebrates and vertebrates

SDP (Specificity diversity permutation) tests at host class level: from sequential comparisons (along the phylogenetic timeline) of two classes (Table 5A-5F)

Table S5A. SDP (Specificity diversity permutation) tests for the US (unique species) in the former class, from sequential comparisons (along the phylogenetic timeline) of two classes

Table S5B. SDP (Specificity diversity permutation) tests for the US (unique species) in the latter class, from sequential comparisons (along the phylogenetic timeline) of two classes

Table S5C. SDP (Specificity diversity permutation) tests for the ES (enriched species) in the former class, from sequential comparisons (along the phylogenetic timeline) of two classes

Table S5D. SDP (Specificity diversity permutation) tests for the ES (enriched species) in the latter class, from sequential comparisons (along the phylogenetic timeline) of two classes

Table S5E. SDP (Specificity diversity permutation) tests for the SD of all species with significant different species in species specificity, from sequential comparisons (along the phylogenetic timeline) of two classes

Table S5F. SDP (Specificity diversity permutation) tests for the SD of all species without considering the differences in specificity, from sequential comparisons (along the phylogenetic timeline) of two classes

SDP (Specificity diversity permutation) tests at host class level: from pairwise comparisons between 10 classes (Table S5G-S5L)

Table S5G. SDP (Specificity diversity permutation) tests for the US (unique species) category in the former, from pairwise comparison between 10 host animal classes □

□

Table S5H. SDP (Specificity diversity permutation) tests for the US (unique species) category in the latter class, from pairwise comparison between 10 host animal classes □

□

Table S5I. SDP (Specificity diversity permutation) tests for the ES (enriched species) category in the former, from pairwise comparison between 10 host animal classes □

□

Table S5J. SDP (Specificity diversity permutation) tests for the ES (enriched species) category in the latter class, from pairwise comparison between 10 host animal classes □

□

Table S5K. SDP (Specificity diversity permutation) tests for all species with significant differences in specificity, from pairwise comparison between 10 host animal classes

Table S5L. SDP (Specificity diversity permutation) tests for all species without considering specificity, from pairwise comparison between 10 host animal classes □

SDP (Specificity diversity permutation) tests for the pairwise comparisons of the three diet types (Table S6A-S6F)

Table S6A. SDP (specificity diversity permutation) tests for the species category of the US (unique species) in the former diet type

Table S6B. SDP (specificity diversity permutation) tests for the species category of the US (unique species) in the latter diet type

Table S6C. SDP (specificity diversity permutation) tests for the species category of the ES (enriched species) in the former diet type

Table S6D. SDP (specificity diversity permutation) tests for the species category of the ES (enriched species) in the latter diet type

Table S6E. SDP (specificity diversity permutation) tests for the species category of all species with significant differences

Table S6F. SDP (specificity diversity permutation) tests for all species (without considering species specificity)

Table S7. The relationships between the phylogenetic timeline (PT) and AGM (animal gastrointestinal microbiome) specificity diversity (SD) (PTSD) for each diet type and
for all diet types pooled together

Table S8. Summary of the animal gut microbiome datasets

Table S9. The number of AGM species in each species category, classified based on specificity permutation (SP) test with FDR (false discovery rate control) corrections at *P*-value=0.05

Table S10. Number of exclusively unique species (EUS) in each animal class

Table S1A. The number of AGM species in each species category, classified based on specificity permutation (SP) test with FDR (false discovery rate control) corrections at *P*-value=0.05

**Comparisons (Former *vs*. Latter)**	**With significant differences in specificity**				**With Significant Difference in Specificity**	**Without Significant difference in Specificity**	**Total Species**
	**Unique in the former**	**Unique in the latter**	**Enriched in the former**	**Enriched in the latter**			
Apis mellifera vs. *Bos taurus* (two representative species)							
Apis mellifera vs. Bos taurus	159	23171	0	0	23330	1064	24394
A total of 45 pairwise comparisons of the AGMs between 10 host animal classes							
Chromadorea vs. Arachnida	43	3636	0	0	3679	7427	11106
Chromadorea vs. Malacostraca	12	3551	5	62	3630	7391	11021
Chromadorea vs. Insecta	6867	110	199	2	7178	36001	43179
Chromadorea vs. Chondrichthyes	20	2711	0	0	2731	7450	10181
Chromadorea vs. Actinopteri	7188	325	121	0	7634	49651	57285
Chromadorea vs. Amphibia	6	2798	2	12	2818	7450	10268
Chromadorea vs. Sauropsida	7221	1518	57	27	8823	24118	32941
Chromadorea vs. Aves	7427	704	15	0	8146	28579	36725
Chromadorea vs. Mammalia	7269	15	66	0	7350	207815	215165
Arachnida vs. Malacostraca	89	3737	0	1	3827	3555	7382
Arachnida vs. Insecta	3569	0	17	0	3586	36295	39881
Arachnida vs. Chondrichthyes	82	2709	0	2	2793	3561	6354
Arachnida vs. Actinopteri	3584	26	24	0	3634	50047	53681
Arachnida vs. Amphibia	40	2842	0	0	2882	3605	6487
Arachnida vs. Sauropsida	3578	134	1	10	3723	25575	29298
Arachnida vs. Aves	3638	27	1	0	3666	29270	32936
Arachnida vs. Mammalia	3606	0	6	0	3612	207890	211502
Malacostraca vs. Insecta	3260	29127	375	0	32762	6810	39572
Malacostraca vs. Chondrichthyes	3701	44	0	8	3753	2659	6412
Malacostraca vs. Actinopteri	3571	42000	136	0	45707	7961	53668
Malacostraca vs. Amphibia	110	2827	2	1	2940	3631	6571
Malacostraca vs. Sauropsida	3629	58	42	2	3731	25618	29349
Malacostraca vs. Aves	3699	4	20	0	3723	29274	32997
Malacostraca vs. Mammalia	3509	178878	180	0	182567	28838	211405
Insecta vs. Chondrichthyes	0	2613	0	21	2634	36291	38925
Insecta vs. Actinopteri	35467	3265	80	21	38833	46731	85564
Insecta vs. Amphibia	31304	2752	0	85	34141	4923	39064
Insecta vs. Sauropsida	322	25076	7	281	25686	35702	61388
Insecta vs. Aves	996	29180	6	2	30184	35308	65492
Insecta vs. Mammalia	35835	3337	54	21	39247	204484	243731
Chondrichthyes vs. Actinopteri	2489	41515	212	0	44216	8370	52586
Chondrichthyes vs. Amphibia	30	2844	0	0	2874	2681	5555
Chondrichthyes vs. Sauropsida	2708	59	0	0	2767	25661	28428
Chondrichthyes vs. Aves	2627	1	32	0	2660	29265	31925
Chondrichthyes vs. Mammalia	1666	173578	849	0	176093	33469	209562
Actinopteri vs. Amphibia	44454	2200	0	612	47266	5031	52297
Actinopteri vs. Sauropsida	727	25522	1	85	26335	49284	75619
Actinopteri vs. Aves	1345	29249	3	4	30601	48745	79346
Actinopteri vs. Mammalia	49149	7818	33	43	57043	200002	257045

Amphibia vs. Sauropsida	2811	26	4	0	2841	25690	28531
Amphibia vs. Aves	2839	0	5	0	2844	29293	32137
Amphibia vs. Mammalia	489	0	46	0	535	210093	210628
Sauropsida vs. Aves	25609	1444	38	0	27091	27816	54907
Sauropsida vs. Mammalia	25081	135	171	1	25388	207589	232977
Aves vs. Mammalia	28272	553	12	1	28838	207330	236168
**Mean**	8220.8	13976.6	62.7	29.0	22289.2	51427.3	73716.5
**Standard Error**	1933.2	5543.8	21.2	14.9	5629.9	10262.5	11864.5
The 9 sequential (along the phylogenetic timeline) class-level comparisons selected from the previous 45 pairwise class							
Chromadorea vs. Arachnida	43	3636	0	0	3679	7427	11106
Arachnida vs. Malacostraca	89	3737	0	1	3827	3555	7382
Malacostraca vs. Insecta	3260	29127	375	0	32762	6810	39572
Insecta vs. Chondrichthyes	0	2613	0	21	2634	36291	38925
Chondrichthyes vs. Actinopteri	2489	41515	212	0	44216	8370	52586
Actinopteri vs. Amphibia	44454	2200	0	612	47266	5031	52297
Amphibia vs. Sauropsida	2811	26	4	0	2841	25690	28531
Sauropsida vs. Aves	25609	1444	38	0	27091	27816	54907
Aves vs. Mammalia	28272	553	12	1	28838	207330	236168
**Mean**	11891.889	9427.889	71.222	70.556	21461.556	36480.000	57941.556
**Standard Error**	5506.645	5018.240	44.335	67.719	6152.953	21720.734	23018.201
Pairwise comparisons of the three diet types							
Carnivore *vs.* Herbivore	64500	8122	512	271	73405	168228	241633
Carnivore *vs.* Omnivore	63821	7300	1021	615	72757	111688	184445
Herbivore *vs.* Omnivore	8836	105874	616	820	116146	167073	283219
Invertebrates vs. Vertebrates							
Invertebrates and Vertebrates	47632	2237	343	84	50296	311220	361516

Table S2E. The catalogues of microbial phyla and classes of top 100 microbial species with the highest specificity from comparing *Apis mellifera* vs. *Bos taurus* (the numbers inside the parentheses represent the number of species belonging to that taxon).

Taxon Level	*Apis mellifera*	*Bos taurus*
**Phylum**	*Proteobacteria (28)*<br>*OD1 (24)*<br>*Bacteria (20)*<br>*Firmicutes (8)*<br>*Actinobacteria (8)*<br>*Bacteroidetes (6)*<br>*TM7 (2)*<br>*Verrucomicrobia (1)*<br>*Spirochaetes (1)*<br>*Cyanobacteria (1)*<br>*Acidobacteria (1)*	*Firmicutes (53)*<br>*Bacteroidetes (29)*<br>*Proteobacteria (5)*<br>*Spirochaetes (4)*<br>*Verrucomicrobia (2)*<br>*Fibrobacteres (2)*<br>*Actinobacteria (2)*<br>*Synergistetes (1)*<br>*Cyanobacteria (1)*<br>*Bacteria (1)*
**Class**	*Bacteria (44)*<br>*Alphaproteobacteria (14)*<br>*Gammaproteobacteria (8)*<br>*Bacilli (8)*<br>*Actinobacteria (8)*<br>*Betaproteobacteria (3)*<br>*[Saprospirae] (2)*<br>*TM7-3 (2)*<br>*Epsilonproteobacteria (2)*<br>*Cytophagia (2)*<br>*[Chloracidobacteria] (1)*<br>*Verrucomicrobiae (1)*<br>*Spirochaetes (1)*<br>*Sphingobacteriia (1)*<br>*ML635J-21 (1)*<br>*Flavobacteriia (1)*<br>*Deltaproteobacteria (1)*	*Clostridia (51)*<br>*Bacteroidia (28)*<br>*Spirochaetes (3)*<br>*Verruco-5 (2)*<br>*Fibrobacteria (2)*<br>*Betaproteobacteria (2)*<br>*Actinobacteria (2)*<br>*Synergistia (1)*<br>*MVP-15 (1)*<br>*Flavobacteriia (1)*<br>*Erysipelotrichi (1)*<br>*Epsilonproteobacteria (1)*<br>*Deltaproteobacteria (1)*<br>*Chloroplast (1)*<br>*Bacteria (1)*<br>*Bacilli (1)*<br>*Alphaproteobacteria (1)*

Table S2F. The catalogues of microbial phyla and classes of top 100 microbial species with the highest specificity from pairwise comparisons between 10 animal classes (the numbers inside the parentheses represent the number of microbial species belonging to that phylum or class).

	Chromadore	*Arachnida*	*Malacostraca*	*Insecta*	*Chondrichthyes*
Phylum level	Proteobacteria (65)<br>Bacteroidetes (27)<br>Firmicutes (3)<br>Actinobacteria (3)<br>Planctomycetes (1)<br>Deferribacteres (1)	Proteobacteria (72)<br>Tenericutes (9)<br>Bacteroidetes (6)<br>Actinobacteria (6)<br>Firmicutes (4)<br>Planctomycetes (2)<br>Verrucomicrobia (1)	Proteobacteria (35)<br>Firmicutes (22)<br>Bacteroidetes (21)<br>Actinobacteria (10)<br>Tenericutes (5)<br>Fusobacteria (3)<br>Cyanobacteria (2)<br>Verrucomicrobia (1)<br>Bacteria (1)	Proteobacteria (37)<br>OD1 (16)<br>Firmicutes (16)<br>Bacteria (13)<br>Actinobacteria (7)<br>Bacteroidetes (6)<br>Verrucomicrobia (1)<br>TM7 (1)<br>Spirochaetes (1)<br>Cyanobacteria (1)<br>Acidobacteria (1)	Firmicutes (54)<br>Bacteroidetes (25)<br>Proteobacteria (9)<br>Tenericutes (6)<br>Bacteria (2)<br>Actinobacteria (2)<br>Lentisphaerae (1)<br>Euryarchaeota (1)
	Actinopteri	*Amphibia*	*Sauropsida*	*Aves*	*Mammalia*
	Firmicutes (58)<br>Proteobacteria (29)<br>Actinobacteria (6)<br>Fusobacteria (3)<br>Cyanobacteria (3)<br>Bacteroidetes (1)	Proteobacteria (46)<br>Firmicutes (23)<br>Bacteroidetes (13)<br>Bacteria (6)<br>Cyanobacteria (3)<br>Synergistetes (2)<br>Planctomycetes (2)<br>Verrucomicrobia (1)<br>Tenericutes (1)<br>GN02 (1)<br>Deferribacteres (1)<br>Actinobacteria (1)	Firmicutes (45)<br>Bacteroidetes (36)<br>Proteobacteria (10)<br>Verrucomicrobia (4)<br>Cyanobacteria (3)<br>Tenericutes (1)<br>Bacteria (1)	Firmicutes (59)<br>Proteobacteria (15)<br>Bacteroidetes (10)<br>Cyanobacteria (5)<br>Verrucomicrobia (4)<br>Actinobacteria (2)<br>Tenericutes (1)<br>Euryarchaeota (1)<br>Chlamydiae (1)<br>Bacteria (1)	Firmicutes (34)<br>Proteobacteria (20)<br>Bacteria (16)<br>Bacteroidetes (13)<br>Actinobacteria (9)<br>Tenericutes (3)<br>Fusobacteria (2)<br>OD1 (1)<br>Cyanobacteria (1)<br>Crenarchaeota (1)
Class level	*Chromadore*	*Arachnida*	*Malacostraca*	*Insecta*	*Chondrichthyes*
	Alphaproteobacteria (31)<br>Gammaproteobacteria (28)<br>Flavobacteriia (14)<br>[Saprospirae] (8)<br>Sphingobacteriia (5)<br>Betaproteobacteria (4)<br>Bacilli (3)<br>Actinobacteria (3)<br>Planctomycetia (1)<br>Deltaproteobacteria (1)<br>Deferribacteres (1)<br>Bacteria (1)	Alphaproteobacteria (41)<br>Gammaproteobacteria (16)<br>Betaproteobacteria (14)<br>Mollicutes (9)<br>Actinobacteria (6)<br>Cytophagia (5)<br>Bacilli (4)<br>Phycisphaerae (2)<br>[Spartobacteria] (1)<br>Flavobacteriia (1)<br>Deltaproteobacteria (1)	Gammaproteobacteria (17)<br>Clostridia (12)<br>Alphaproteobacteria (11)<br>Flavobacteriia (10)<br>Bacteroidia (10)<br>Actinobacteria (10)<br>Bacilli (9)<br>Mollicutes (5)<br>Betaproteobacteria (4)<br>Bacteria (4)<br>Fusobacteriia (3)<br>Chloroplast (2)<br>Verrucomicrobiae (1)<br>Erysipelotrichi (1)<br>Epsilonproteobacteria (1)	Bacteria (30)<br>Gammaproteobacteria (16)<br>Bacilli (13)<br>Alphaproteobacteria (13)<br>Actinobacteria (7)<br>Betaproteobacteria (5)<br>[Saprospirae] (2)<br>Epsilonproteobacteria (2)<br>Cytophagia (2)<br>Clostridia (2)<br>[Chloracidobacteria] (1)<br>Verrucomicrobiae (1)<br>TM7-3 (1)<br>Spirochaetes (1)<br>Sphingobacteriia (1)<br>ML635J-21 (1)<br>Flavobacteriia (1)<br>Deltaproteobacteria (1)	Clostridia (41)<br>Bacteroidia (24)<br>Bacilli (8)<br>Mollicutes (6)<br>Erysipelotrichi (5)<br>Gammaproteobacteria (4)<br>Alphaproteobacteria (4)<br>Bacteria (2)<br>Actinobacteria (2)<br>[Lentisphaeria] (1)<br>Sphingobacteriia (1)<br>Methanobacteria (1)<br>Betaproteobacteria (1)
	Actinopteri	*Amphibia*	*Sauropsida*	*Aves*	*Mammalia*
	Bacilli (42)<br>Gammaproteobacteria (20)<br>Clostridia (16)<br>Alphaproteobacteria (7)<br>Coriobacteriia (4)<br>Fusobacteriia (3)<br>Chloroplast (3)<br>Betaproteobacteria (2)<br>Actinobacteria (2)<br>Bacteroidia (1)	Clostridia (20)<br>Deltaproteobacteria (16)<br>Gammaproteobacteria (12)<br>Betaproteobacteria (10)<br>Bacteroidia (8)<br>Alphaproteobacteria (8)<br>Bacteria (6)<br>Flavobacteriia (5)<br>Chloroplast (3)<br>Bacilli (3)<br>Synergistia (2)<br>vadinHA49 (1)<br>Verrucomicrobiae (1)<br>Planctomycetia (1)<br>Mollicutes (1)<br>Deferribacteres (1)<br>Coriobacteriia (1)<br>BD1-5 (1)	Clostridia (39)<br>Bacteroidia (36)<br>Bacilli (5)<br>Verrucomicrobiae (4)<br>Gammaproteobacteria (4)<br>Chloroplast (3)<br>Alphaproteobacteria (3)<br>Deltaproteobacteria (2)<br>Mollicutes (1)<br>Erysipelotrichi (1)<br>Epsilonproteobacteria (1)<br>Bacteria (1)	Clostridia (41)<br>Bacilli (17)<br>Bacteroidia (7)<br>Gammaproteobacteria (6)<br>Chloroplast (5)<br>Verrucomicrobiae (4)<br>Alphaproteobacteria (4)<br>Epsilonproteobacteria (3)<br>[Saprospirae] (2)<br>Betaproteobacteria (2)<br>Bacteria (2)<br>Actinobacteria (2)<br>Mollicutes (1)<br>Methanobacteria (1)<br>Erysipelotrichi (1)<br>Chlamydiia (1)	Clostridia (22)<br>Bacteria (18)<br>Gammaproteobacteria (10)<br>Bacteroidia (9)<br>Bacilli (9)<br>Actinobacteria (5)<br>Coriobacteriia (4)<br>Betaproteobacteria (4)<br>Mollicutes (3)<br>Erysipelotrichi (3)<br>Alphaproteobacteria (3)<br>Fusobacteriia (2)<br>Flavobacteriia (2)<br>Epsilonproteobacteria (2)<br>Sphingobacteriia (1)<br>MHVG (1)<br>Deltaproteobacteria (1)<br>Chloroplast (1)

Table S2G. The catalogues of microbial phyla and classes of top 100 microbial species with the highest specificity from pairwise comparisons of the three diet types (the numbers inside the parentheses represent the number of microbial species belonging to that phylum or class).

Taxon	Carnivore	Herbivore	Omnivore
Phylum	Firmicutes (57)<br>Proteobacteria (31)<br>Actinobacteria (6)<br>Fusobacteria (3)<br>Cyanobacteria (3)	Proteobacteria (36)<br>Firmicutes (25)<br>Bacteroidetes (11)<br>Bacteria (9)<br>OD1 (7)<br>Actinobacteria (4)<br>Verrucomicrobia (3)<br>Fusobacteria (3)<br>Tenericutes (1)<br>Planctomycetes (1)	Firmicutes (57)<br>Proteobacteria (14)<br>Bacteroidetes (12)<br>Verrucomicrobia (4)<br>Bacteria (4)<br>Tenericutes (3)<br>Actinobacteria (3)<br>Euryarchaeota (1)<br>Cyanobacteria (1)<br>Chlamydiae (1)
Class	Bacilli (41)<br>Gammaproteobacteria (21)<br>Clostridia (16)<br>Alphaproteobacteria (8)<br>Coriobacteriia (4)<br>Fusobacteriia (3)<br>Chloroplast (3)<br>Betaproteobacteria (2)<br>Actinobacteria (2)	Gammaproteobacteria (21)<br>Bacilli (18)<br>Bacteria (16)<br>Betaproteobacteria (7)<br>Alphaproteobacteria (7)<br>Bacteroidia (6)<br>Clostridia (5)<br>Actinobacteria (4)<br>Verrucomicrobiae (3)<br>Fusobacteriia (3)<br>Saprospirae (2)<br>Erysipelotrichi (2)<br>Cytophagia (2)<br>Planctomycetia (1)<br>Flavobacteriia (1)<br>Epsilonproteobacteria (1)<br>CK-1C4-19 (1)	Clostridia (37)<br>Bacilli (18)<br>Bacteroidia (10)<br>Gammaproteobacteria (8)<br>Verrucomicrobiae (4)<br>Bacteria (4)<br>Mollicutes (3)<br>Betaproteobacteria (3)<br>Flavobacteriia (2)<br>Erysipelotrichi (2)<br>Coriobacteriia (2)<br>Alphaproteobacteria (2)<br>Methanobacteria (1)<br>Epsilonproteobacteria (1)<br>Chloroplast (1)<br>Chlamydiia (1)<br>Actinobacteria (1)

Table S2H. The catalogues of microbial phyla and classes of top 100 microbial species with the highest specificity from comparing invertebrates and vertebrates (the numbers inside the parentheses represent the number of microbial species belonging to that phylum or class).

Taxon	Invertebrates	Vertebrates
Phylum	Proteobacteria (43)<br>OD1 (13)<br>Bacteria (13)<br>Firmicutes (11)<br>Actinobacteria (9)<br>Bacteroidetes (7)<br>Verrucomicrobia (1)<br>TM7 (1)<br>Spirochaetes (1)<br>Cyanobacteria (1)	Firmicutes (56)<br>Proteobacteria (26)<br>Actinobacteria (6)<br>Cyanobacteria (4)<br>Fusobacteria (3)<br>Bacteroidetes (3)<br>Verrucomicrobia (1)<br>Bacteria (1)
Class	Bacteria (27)<br>Gammaproteobacteria (21)<br>Alphaproteobacteria (13)<br>Bacilli (10)<br>Actinobacteria (9)<br>Betaproteobacteria (7)<br>[Saprospirae] (2)<br>Sphingobacteriia (2)<br>Cytophagia (2)<br>Verrucomicrobiae (1)<br>TM7-3 (1)<br>Spirochaetes (1)<br>Flavobacteriia (1)<br>Epsilonproteobacteria (1)<br>Deltaproteobacteria (1)<br>Chloroplast (1)	Bacilli (38)<br>Gammaproteobacteria (20)<br>Clostridia (18)<br>Alphaproteobacteria (5)<br>Coriobacteriia (4)<br>Chloroplast (4)<br>Fusobacteriia (3)<br>Bacteroidia (3)<br>Actinobacteria (2)<br>Verrucomicrobiae (1)<br>Betaproteobacteria (1)<br>Bacteria (1)

SDP (specificity diversity permutation) tests at host species level: from comparing two representative species (*Apis mellifera* and *Bos taurus*)

Table S3. Permutation tests for the *specificity diversity* (SD) from comparison between *Apis mellifera* and *Bos taurus*

Category	Comparison	Order	Former	Latter	Delta	Lower (95%)	Upper (95%)	*p*-Value
Unique species in the former case	*Apis mellifera vs. Bos taurus*	***q=0***	159.000	0.000	159.000	-1.389	3.819	0.000
		q=1	118.222	0.000	118.222	-12.382	23.206	0.000
		q=2	96.542	0.000	96.542	-16.227	25.381	0.000
		q=3	84.496	0.000	84.496	-18.088	25.892	0.000
		q=4	76.988	0.000	76.988	-19.139	26.083	0.000
Unique species in the latter case	*Apis mellifera vs. Bos taurus*	***q=0***	0.000	23171.000	-23171.000	2983.006	13757.642	0.000
		q=1	0.000	19572.190	-19572.190	2437.328	11914.815	0.000
		q=2	0.000	15129.575	-15129.575	1579.598	9920.713	0.000
		q=3	0.000	11527.710	-11527.710	260.419	8285.790	0.000
		q=4	0.000	9323.779	-9323.779	-783.288	7003.325	0.000
Enriched species in the former case	*Apis mellifera vs. Bos taurus*	***q=0***	NA	NA	NA	NA	NA	NA
		q=1	NA	NA	NA	NA	NA	NA
		q=2	NA	NA	NA	NA	NA	NA
		q=3	NA	NA	NA	NA	NA	NA
		q=4	NA	NA	NA	NA	NA	NA
Enriched species in the latter case	*Apis mellifera vs. Bos taurus*	***q=0***	NA	NA	NA	NA	NA	NA
		q=1	NA	NA	NA	NA	NA	NA
		q=2	NA	NA	NA	NA	NA	NA
		q=3	NA	NA	NA	NA	NA	NA
		q=4	NA	NA	NA	NA	NA	NA
All species with significant differences	*Apis mellifera vs. Bos taurus*	***q=0***	159.000	23171.000	-23012.000	2984.404	13758.674	0.000
		q=1	118.222	19572.190	-19453.968	29.901	10922.262	0.000
		q=2	96.542	15129.575	-15033.033	-3646.277	5871.714	0.000
		q=3	84.496	11527.710	-11443.214	-2512.788	2644.067	0.000
		q=4	76.988	9323.779	-9246.792	-1565.824	1592.234	0.000
All species	*Apis mellifera vs. Bos taurus*	***q=0***	1223.000	23171.000	-21948.000	3395.156	14088.284	0.000
		q=1	293.185	19572.190	-19279.005	364.136	11171.475	0.000
		q=2	151.339	15129.575	-14978.236	-3589.633	6038.371	0.000
		q=3	119.274	11527.710	-11408.436	-2556.375	2715.972	0.000
		q=4	104.615	9323.779	-9219.164	-1593.105	1629.105	0.000
Percentage of Significant Differences (%)		***q=0***						100%
		q=1						100%
		q=2						100%
		q=3						100%
		q=4						100%

SDP (specificity diversity permutation) tests for the SD (specificity diversity) from comparing between invertebrates and vertebrates

Table S4. SDP (Specificity diversity permutation) tests of the SD from comparing between invertebrates and vertebrates

Groups	Treatments	Order	Former	Latter	Delta	Lower (95%)	Upper (95%)	*p*-Value
Unique species in the former case	Invertebrates vs. Vertebrates	*q=0*	47632.000	0.000	47632.000	-26568.208	-16803.884	0.000
		q=1	24494.113	0.000	24494.113	-17663.048	-8899.786	0.000
		q=2	7218.507	0.000	7218.507	-7358.951	313.059	0.026
		q=3	3199.287	0.000	3199.287	-3454.563	2060.218	0.034
		q=4	2097.086	0.000	2097.086	-2246.275	1598.395	0.035
Unique species in the latter case	Invertebrates vs. Vertebrates	*q=0*	0.000	2237.000	-2237.000	-3.196	1.700	0.000
		q=1	0.000	1787.354	-1787.354	-288.247	88.469	0.000
		q=2	0.000	1305.652	-1305.652	-407.454	236.868	0.000
		q=3	0.000	945.973	-945.973	-399.029	293.687	0.000
		q=4	0.000	740.184	-740.184	-344.705	277.791	0.000
Enriched species in the former case	Invertebrates vs. Vertebrates	*q=0*	343.000	343.000	0.000	-35.354	-6.796	1.000
		q=1	223.481	53.467	170.014	-81.368	8.419	0.000
		q=2	146.552	21.755	124.797	-71.844	19.055	0.000
		q=3	108.723	14.845	93.878	-59.783	20.858	0.000
		q=4	89.698	12.284	77.414	-51.674	20.254	0.000
Enriched species in the latter case	Invertebrates vs. Vertebrates	*q=0*	84.000	84.000	0.000	-0.063	0.061	0.001
		q=1	7.059	73.234	-66.175	-12.841	8.052	0.000
		q=2	4.567	63.522	-58.955	-17.249	12.283	0.000
		q=3	4.063	55.978	-51.916	-17.675	13.545	0.000
		q=4	3.821	50.642	-46.820	-16.811	13.390	0.000
All species with significant differences	Invertebrates vs. Vertebrates	*q=0*	48059.000	2664.000	45395.000	-26593.927	-16821.813	0.000
		q=1	23273.631	1868.530	21405.100	-8616.186	-1647.930	0.000
		q=2	6852.788	1372.286	5480.503	-2521.154	1317.686	0.000
		q=3	3191.043	1001.492	2189.551	-1538.328	1119.026	0.005
		q=4	2146.730	785.536	1361.194	-1156.667	913.472	0.012
All species	Invertebrates vs. Vertebrates	*q=0*	49930.000	313884.000	-263954.000	-176226.542	-149467.900	0.000
		q=1	24261.580	144611.825	-120350.245	-102714.439	-74273.225	0.000
		q=2	7157.526	35252.006	-28094.480	-31613.141	-825.981	0.067
		q=3	3309.503	13512.746	-10203.243	-13152.843	8789.998	0.085
		q=4	2218.042	8006.035	-5787.992	-8145.423	6505.170	0.125
Percentage of Significant Differences (%)		*q=0*						83.3
		q=1						100
		q=2						83.3
		q=3						83.3
		q=4						83.3

SDP (Specificity diversity permutation) tests at host class level: from sequential comparisons (along the phylogenetic timeline) of two classes (Table S5A-S5F)

Table S5A. SDP (Specificity diversity permutation) tests for the US (unique species) in the former class, from sequential comparisons (along the phylogenetic timeline) of two classes

Treatments	Order	Former	Latter	Delta	Lower (95%)	Upper (95%)	*p*-Value
Chromadorea vs. Arachnida	***q=0***	43.000	NA	43.000	-0.779	0.931	0.000
	q=1	40.118	NA	40.118	-4.548	12.071	0.000
	q=2	37.323	NA	37.323	-6.952	15.776	0.000
	q=3	34.830	NA	34.830	-8.276	17.073	0.000
	q=4	32.755	NA	32.755	-8.915	17.403	0.000
Arachnida vs. Malacostraca	***q=0***	89.000	NA	89.000	-3.658	8.514	0.000
	q=1	72.490	NA	72.490	-8.398	14.814	0.000
	q=2	58.931	NA	58.931	-9.664	15.197	0.000
	q=3	49.657	NA	49.657	-9.649	14.184	0.000
	q=4	43.757	NA	43.757	-9.314	13.110	0.000
Malacostraca vs. Insecta	***q=0***	3260.000	NA	3260.000	-3583.302	-2523.976	0.000
	q=1	2628.254	NA	2628.254	-2944.848	-2047.356	0.079
	q=2	1787.508	NA	1787.508	-2255.613	-1270.100	0.254
	q=3	1197.850	NA	1197.850	-1707.182	-704.767	0.263
	q=4	914.030	NA	914.030	-1321.415	-484.145	0.252
Insecta vs. Chondrichthyes	***q=0***	NA	NA	NA	NA	NA	NA
	q=1	NA	NA	NA	NA	NA	NA
	q=2	NA	NA	NA	NA	NA	NA
	q=3	NA	NA	NA	NA	NA	NA
	q=4	NA	NA	NA	NA	NA	NA
Chondrichthyes vs. Actinopteri	***q=0***	2489.000	NA	2489.000	-2815.590	-1894.042	0.000
	q=1	2023.095	NA	2023.095	-2421.223	-1439.253	0.208
	q=2	1315.179	NA	1315.179	-1798.188	-846.443	0.820
	q=3	748.781	NA	748.781	-1257.160	-339.480	0.834
	q=4	488.211	NA	488.211	-929.640	-121.165	0.834
Actinopteri vs. Amphibia	***q=0***	44454.000	NA	44454.000	42010.910	43979.654	0.000
	q=1	18232.541	NA	18232.541	20880.389	31514.352	0.999
	q=2	1122.761	NA	1122.761	205.653	4580.341	0.887
	q=3	494.794	NA	494.794	-533.587	1388.360	0.471
	q=4	368.554	NA	368.554	-658.991	936.301	0.407
Amphibia vs. Sauropsida	***q=0***	2811.000	NA	2811.000	-3140.444	-1662.696	0.000
	q=1	2203.682	NA	2203.682	-2454.159	-1390.392	0.000
	q=2	1604.776	NA	1604.776	-1874.937	-1025.464	0.159
	q=3	1234.806	NA	1234.806	-1500.918	-730.276	0.217
	q=4	1043.053	NA	1043.053	-1252.099	-561.470	0.152
Sauropsida vs. Aves	***q=0***	25609.000	NA	25609.000	-8422.697	-2932.453	0.000
	q=1	8592.455	NA	8592.455	-3708.383	-817.723	0.000
	q=2	2887.479	NA	2887.479	-1480.274	806.219	0.000
	q=3	1713.761	NA	1713.761	-831.485	655.758	0.000
	q=4	1258.601	NA	1258.601	-604.125	502.751	0.000
Aves vs. Mammalia	***q=0***	28272.000	NA	28272.000	-15587.786	-10303.110	0.000
	q=1	11210.426	NA	11210.426	-8380.032	-3909.138	0.000
	q=2	3727.197	NA	3727.197	-3165.357	586.125	0.006

	q=3	2165.369	NA	2165.369	-1562.918	875.648	0.001
	q=4	1620.910	NA	1620.910	-1072.225	675.645	0.000
Percentage of Significant Differences (%)	***q=0***						100%
	q=1						66.70%
	q=2						55.60%
	q=3						55.60%
	q=4						55.60%

Table S5B. SDP (Specificity diversity permutation) tests for the US (unique species) in the latter class, from sequential comparisons (along the phylogenetic timeline) of two classes

**Treatments**	**Order**	**Former**	**Latter**	**Delta**	**Lower (95%)**	**Upper (95%)**	***p*-Value**
Chromadorea vs. Arachnida	***q=0***	NA	3636.000	-3636.000	1293.799	3283.645	0.000
	q=1	NA	2579.806	-2579.806	1078.306	2528.796	0.000
	q=2	NA	1222.548	-1222.548	670.782	1470.862	0.246
	q=3	NA	617.272	-617.272	162.492	843.017	0.254
	q=4	NA	418.643	-418.643	-60.604	571.986	0.148
Arachnida vs. Malacostraca	***q=0***	NA	3737.000	-3737.000	-657.419	2072.849	0.000
	q=1	NA	2865.794	-2865.794	-647.512	1789.846	0.000
	q=2	NA	1882.244	-1882.244	-699.028	1506.980	0.001
	q=3	NA	1289.770	-1289.770	-802.419	1306.957	0.020
	q=4	NA	1014.249	-1014.249	-837.589	1142.475	0.047
Malacostraca vs. Insecta	***q=0***	NA	29127.000	-29127.000	-29057.106	-25751.190	0.000
	q=1	NA	23334.587	-23334.587	-26606.208	-22316.113	0.861
	q=2	NA	3705.692	-3705.692	-11704.523	-3743.424	0.988
	q=3	NA	1000.733	-1000.733	-2795.231	175.022	0.650
	q=4	NA	620.730	-620.730	-1513.821	660.703	0.438
Insecta vs. Chondrichthyes	***q=0***	NA	2613.000	-2613.000	1836.667	3017.987	0.000
	q=1	NA	2192.388	-2192.388	1398.368	2674.827	0.141
	q=2	NA	1559.268	-1559.268	887.767	2096.566	0.390
	q=3	NA	986.200	-986.200	400.554	1536.496	0.382
	q=4	NA	681.720	-681.720	148.136	1169.175	0.347
Chondrichthyes vs. Actinopteri	***q=0***	NA	41515.000	-41515.000	-40580.656	-38250.444	0.000
	q=1	NA	12691.031	-12691.031	-25355.044	-15549.149	1.000
	q=2	NA	864.075	-864.075	-2959.976	197.109	0.725
	q=3	NA	440.860	-440.860	-1090.226	751.913	0.420
	q=4	NA	342.376	-342.376	-806.527	775.827	0.424
Actinopteri vs. Amphibia	***q=0***	NA	2200.000	-2200.000	1846.237	2411.343	0.000
	q=1	NA	2136.334	-2136.334	1779.782	2348.808	0.000
	q=2	NA	2031.873	-2031.873	1690.554	2237.205	0.000
	q=3	NA	1870.014	-1870.014	1551.529	2065.239	0.014
	q=4	NA	1657.860	-1657.860	1363.593	1842.868	0.035
Amphibia vs. Sauropsida	***q=0***	NA	26.000	-26.000	-0.169	0.159	0.000
	q=1	NA	25.107	-25.107	-4.047	1.435	0.000
	q=2	NA	24.229	-24.229	-5.986	2.404	0.000
	q=3	NA	23.399	-23.399	-7.049	3.153	0.000
	q=4	NA	22.644	-22.644	-7.630	3.699	0.000
Sauropsida vs. Aves	***q=0***	NA	1444.000	-1444.000	-22.763	1.883	0.000
	q=1	NA	1103.048	-1103.048	-118.267	20.371	0.000
	q=2	NA	843.208	-843.208	-128.386	46.111	0.000
	q=3	NA	678.119	-678.119	-127.878	63.852	0.000

	q=4	NA	576.464	-576.464	-127.270	75.519	0.000
Aves vs. Mammalia	***q=0***	NA	553.000	-553.000	-1.799	1.455	0.000
	q=1	NA	512.335	-512.335	-59.785	-3.891	0.000
	q=2	NA	470.845	-470.845	-78.985	1.479	0.000
	q=3	NA	431.723	-431.723	-87.369	11.064	0.000
	q=4	NA	397.485	-397.485	-91.902	21.861	0.000
Percentage of Significant Differences (%)	***q=0***						100%
	q=1						66.70%
	q=2						55.60%
	q=3						55.60%
	q=4						55.60%

Table S5C. SDP (Specificity diversity permutation) tests for the ES (enriched species) in the former class, from sequential comparisons (along the phylogenetic timeline) of two classes

Treatments	Order	Former	Latter	Delta	Lower (95%)	Upper (95%)	*p*-Value
Chromadorea vs. Arachnida	***q=0***	NA	NA	NA	NA	NA	NA
	q=1	NA	NA	NA	NA	NA	NA
	q=2	NA	NA	NA	NA	NA	NA
	q=3	NA	NA	NA	NA	NA	NA
	q=4	NA	NA	NA	NA	NA	NA
Arachnida vs. Malacostraca	***q=0***	NA	NA	NA	NA	NA	NA
	q=1	NA	NA	NA	NA	NA	NA
	q=2	NA	NA	NA	NA	NA	NA
	q=3	NA	NA	NA	NA	NA	NA
	q=4	NA	NA	NA	NA	NA	NA
Malacostraca vs. Insecta	***q=0***	375.000	375.000	0.000	-408.514	-246.972	1.000
	q=1	254.213	34.335	219.878	-343.463	-113.786	0.724
	q=2	182.839	7.950	174.890	-258.847	-40.850	0.419
	q=3	148.351	5.173	143.179	-200.108	11.262	0.172
	q=4	130.287	4.350	125.937	-165.572	36.930	0.129
Insecta vs. Chondrichthyes	***q=0***	NA	NA	NA	NA	NA	NA
	q=1	NA	NA	NA	NA	NA	NA
	q=2	NA	NA	NA	NA	NA	NA
	q=3	NA	NA	NA	NA	NA	NA
	q=4	NA	NA	NA	NA	NA	NA
Chondrichthyes vs. Actinopteri	***q=0***	212.000	212.000	0.000	-256.710	-140.950	1.000
	q=1	174.788	80.996	93.793	-270.980	-82.115	0.910
	q=2	131.396	59.989	71.407	-249.299	-66.333	0.917
	q=3	92.737	52.685	40.053	-221.525	-50.117	0.959
	q=4	69.515	48.701	20.814	-195.057	-33.756	0.979
Actinopteri vs. Amphibia	***q=0***	NA	NA	NA	NA	NA	NA
	q=1	NA	NA	NA	NA	NA	NA
	q=2	NA	NA	NA	NA	NA	NA
	q=3	NA	NA	NA	NA	NA	NA
	q=4	NA	NA	NA	NA	NA	NA
Amphibia vs. Sauropsida	***q=0***	4.000	4.000	0.000	-4.734	-0.562	0.973
	q=1	3.780	2.349	1.430	-4.594	0.354	0.722
	q=2	3.600	2.001	1.598	-4.307	0.549	0.669
	q=3	3.464	1.865	1.598	-4.067	0.610	0.633

	q=4	3.365	1.789	1.576	-3.891	0.635	0.600
Sauropsida vs. Aves	***q=0***	38.000	38.000	0.000	-3.242	1.542	0.565
	q=1	20.088	8.479	11.609	-6.412	4.333	0.000
	q=2	13.650	4.860	8.790	-5.592	4.062	0.000
	q=3	11.192	3.874	7.318	-4.881	3.668	0.000
	q=4	10.028	3.449	6.580	-4.422	3.387	0.000
Aves vs. Mammalia	***q=0***	12.000	12.000	0.000	-7.677	2.895	0.753
	q=1	11.752	7.435	4.317	-9.945	5.278	0.319
	q=2	11.541	6.235	5.307	-10.842	6.499	0.334
	q=3	11.364	5.712	5.653	-11.162	7.004	0.331
	q=4	11.218	5.411	5.807	-11.244	7.193	0.327
Percentage of Significant Differences (%)	***q=0***						44.40%
	q=1						55.60%
	q=2						55.60%
	q=3						55.60%
	q=4						55.60%

Table S5D. SDP (Specificity diversity permutation) tests for the ES (enriched species) in the latter class, from sequential comparisons (along the phylogenetic timeline) of two classes

Treatments	Order	Former	Latter	Delta	Lower (95%)	Upper (95%)	*p*-Value
Chromadorea vs. Arachnida	***q=0***	NA	NA	NA	NA	NA	NA
	q=1	NA	NA	NA	NA	NA	NA
	q=2	NA	NA	NA	NA	NA	NA
	q=3	NA	NA	NA	NA	NA	NA
	q=4	NA	NA	NA	NA	NA	NA
Arachnida vs. Malacostraca	***q=0***	1.000	1.000	0.000	-0.230	0.262	0.016
	q=1	1.000	1.000	0.000	-0.230	0.262	0.016
	q=2	1.000	1.000	0.000	-0.230	0.262	0.016
	q=3	1.000	1.000	0.000	-0.230	0.262	0.016
	q=4	1.000	1.000	0.000	-0.230	0.262	0.016
Malacostraca vs. Insecta	***q=0***	NA	NA	NA	NA	NA	NA
	q=1	NA	NA	NA	NA	NA	NA
	q=2	NA	NA	NA	NA	NA	NA
	q=3	NA	NA	NA	NA	NA	NA
	q=4	NA	NA	NA	NA	NA	NA
Insecta vs. Chondrichthyes	***q=0***	21.000	21.000	0.000	9.674	22.980	1.000
	q=1	6.064	13.187	-7.124	4.494	17.817	0.867
	q=2	4.874	9.216	-4.342	2.447	14.714	0.896
	q=3	4.501	7.251	-2.750	1.401	12.882	0.916
	q=4	4.331	6.228	-1.897	0.814	11.722	0.928
Chondrichthyes vs. Actinopteri	***q=0***	NA	NA	NA	NA	NA	NA
	q=1	NA	NA	NA	NA	NA	NA
	q=2	NA	NA	NA	NA	NA	NA
	q=3	NA	NA	NA	NA	NA	NA
	q=4	NA	NA	NA	NA	NA	NA
Actinopteri vs. Amphibia	***q=0***	612.000	612.000	0.000	344.732	742.002	1.000
	q=1	300.910	493.278	-192.368	185.684	599.088	0.924
	q=2	184.187	425.059	-240.872	107.780	508.836	0.740
	q=3	119.055	386.220	-267.165	52.048	467.199	0.661
	q=4	86.333	362.273	-275.941	11.546	447.942	0.625

Amphibia vs. Sauropsida	***q=0***	NA	NA	NA	NA	NA	NA
	q=1	NA	NA	NA	NA	NA	NA
	q=2	NA	NA	NA	NA	NA	NA
	q=3	NA	NA	NA	NA	NA	NA
	q=4	NA	NA	NA	NA	NA	NA
Sauropsida vs. Aves	***q=0***	NA	NA	NA	NA	NA	NA
	q=1	NA	NA	NA	NA	NA	NA
	q=2	NA	NA	NA	NA	NA	NA
	q=3	NA	NA	NA	NA	NA	NA
	q=4	NA	NA	NA	NA	NA	NA
Aves vs. Mammalia	***q=0***	1.000	1.000	NA	NA	NA	NA
	q=1	1.000	1.000	NA	NA	NA	NA
	q=2	1.000	1.000	NA	NA	NA	NA
	q=3	1.000	1.000	NA	NA	NA	NA
	q=4	1.000	1.000	NA	NA	NA	NA
Percentage of Significant Differences (%)	***q=0***						77.80%
	q=1						77.80%
	q=2						77.80%
	q=3						77.80%
	q=4						77.80%

Table S5E. SDP (Specificity diversity permutation) tests for the SD of all species with significant different species in species specificity, from sequential comparisons (along the phylogenetic timeline) of two classes

Treatments	Order	Former	Latter	Delta	Lower (95%)	Upper (95%)	*p*-Value
Chromadorea vs. Arachnida	***q=0***	43.000	3636.000	-3593.000	1293.924	3283.672	0.000
	q=1	40.118	2579.806	-2539.688	324.168	2093.528	0.000
	q=2	37.323	1222.548	-1185.225	-447.740	895.032	0.000
	q=3	34.830	617.272	-582.441	-454.290	495.954	0.004
	q=4	32.755	418.643	-385.888	-361.564	362.091	0.022
Arachnida vs. Malacostraca	***q=0***	90.000	3738.000	-3648.000	-653.267	2073.585	0.000
	q=1	72.513	2864.896	-2792.383	-784.875	1824.486	0.000
	q=2	58.937	1881.539	-1822.602	-965.770	1538.759	0.004
	q=3	49.660	1290.167	-1240.507	-1024.901	1271.879	0.029
	q=4	43.760	1015.110	-971.350	-944.129	1063.927	0.050
Malacostraca vs. Insecta	***q=0***	3635.000	29502.000	-25867.000	-32492.367	-29078.693	1.000
	q=1	2780.448	23364.353	-20583.905	-28248.187	-24021.699	1.000
	q=2	1829.602	3713.604	-1884.002	-12745.008	-5115.130	1.000
	q=3	1259.707	1003.952	255.755	-3423.617	-21.565	0.970
	q=4	993.646	622.613	371.033	-1808.959	639.141	0.704
Insecta vs. Chondrichthyes	***q=0***	21.000	2634.000	-2613.000	307.597	3821.119	0.208
	q=1	6.064	2147.425	-2141.362	1338.044	2597.755	0.131
	q=2	4.874	1412.855	-1407.981	713.323	1889.547	0.321
	q=3	4.501	812.530	-808.029	228.042	1281.484	0.375
	q=4	4.331	529.854	-525.523	22.818	941.790	0.373
Chondrichthyes vs. Actinopteri	***q=0***	2701.000	41727.000	-39026.000	-43242.785	-40695.607	1.000
	q=1	2197.508	12709.804	-10512.296	-27509.738	-17404.032	1.000
	q=2	1441.958	864.746	577.212	-3435.683	158.049	0.887
	q=3	827.098	441.117	385.981	-1232.307	820.907	0.525

	q=4	538.746	342.554	196.192	-899.299	852.106	0.675
Actinopteri vs. Amphibia	***q=0***	45066.000	2812.000	42254.000	44615.222	46719.656	1.000
	q=1	18328.557	2221.809	16106.748	22185.207	32064.479	1.000
	q=2	1126.269	1634.016	-507.747	372.923	5336.654	0.990
	q=3	495.959	1263.395	-767.437	-591.313	1644.740	0.336
	q=4	369.325	1068.388	-699.063	-742.603	1088.708	0.172
Amphibia vs. Sauropsida	***q=0***	2815.000	30.000	2785.000	-3143.134	-1665.312	0.238
	q=1	2206.819	25.115	2181.704	-1499.451	-24.774	0.000
	q=2	1608.146	24.231	1583.916	-500.312	421.681	0.000
	q=3	1238.270	23.400	1214.870	-314.553	348.389	0.000
	q=4	1046.315	22.645	1023.670	-242.623	274.043	0.000
Sauropsida vs. Aves	***q=0***	25647.000	1482.000	24165.000	-8432.182	-2945.548	0.000
	q=1	8393.153	1103.853	7289.300	-3410.632	-203.899	0.000
	q=2	2760.423	843.453	1916.970	-1169.077	520.202	0.000
	q=3	1610.229	678.269	931.960	-691.366	431.780	0.003
	q=4	1168.996	576.578	592.418	-559.122	396.705	0.019
Aves vs. Mammalia	***q=0***	28285.000	566.000	27719.000	-15590.543	-10305.479	0.000
	q=1	11221.702	513.316	10708.386	-6808.975	-2475.485	0.000
	q=2	3732.169	471.722	3260.447	-2257.274	583.668	0.002
	q=3	2167.821	432.507	1735.314	-1212.330	577.506	0.004
	q=4	1622.553	398.181	1224.372	-885.496	426.871	0.003
Percentage of Significant Differences (%)	***q=0***						44.40%
	q=1						55.60%
	q=2						55.60%
	q=3						55.60%
	q=4						44.40%

Table S5F. SDP (Specificity diversity permutation) tests for the SD of all species without considering the differences in specificity, from sequential comparisons (along the phylogenetic timeline) of two classes

Treatments	Order	Former	Latter	Delta	Lower (95%)	Upper (95%)	*p*-Value
Chromadorea vs. Arachnida	***q=0***	7470.000	3645.000	3825.000	5602.298	8120.934	1.000
	q=1	3608.169	2583.698	1024.471	2854.481	5275.554	1.000
	q=2	1425.619	1224.139	201.480	274.601	2404.208	0.979
	q=3	816.322	617.931	198.391	-514.008	1269.942	0.759
	q=4	589.355	419.042	170.313	-592.067	929.104	0.700
Arachnida vs. Malacostraca	***q=0***	3645.000	3744.000	-99.000	92.793	2699.905	0.980
	q=1	2585.378	2868.068	-282.691	-86.877	2321.408	0.924
	q=2	1225.049	1883.387	-658.338	-456.512	1872.326	0.537
	q=3	618.319	1291.332	-673.012	-886.839	1539.060	0.348
	q=4	419.277	1015.955	-596.678	-1030.074	1324.740	0.355
Malacostraca vs. Insecta	***q=0***	3744.000	36312.000	-32568.000	-38567.074	-34670.776	1.000
	q=1	2843.815	17233.712	-14389.898	-22528.869	-18335.759	1.000
	q=2	1864.807	4804.931	-2940.124	-8596.649	-4184.910	0.998
	q=3	1280.164	2215.750	-935.586	-4047.122	-414.022	0.920
	q=4	1008.118	1502.936	-494.818	-2639.740	467.625	0.798
Insecta vs. Chondrichthyes	***q=0***	36312.000	2711.000	33601.000	33907.250	37836.096	0.977
	q=1	17388.383	2196.835	15191.548	17933.916	22228.077	1.000
	q=2	4885.541	1440.938	3444.604	4011.349	8459.091	0.990
	q=3	2254.721	826.352	1428.370	336.252	4005.356	0.780

	q=4	1528.149	537.930	990.219	-511.129	2644.386	0.555
Chondrichthyes vs. Actinopteri	***q=0***	2711.000	50097.000	-47386.000	-50610.398	-47342.164	0.953
	q=1	2205.342	13785.932	-11580.591	-21071.891	-15496.576	1.000
	q=2	1446.782	2755.660	-1308.878	-5425.178	-1049.723	0.961
	q=3	829.509	1384.645	-555.136	-2590.871	760.584	0.693
	q=4	540.155	991.533	-451.378	-1836.494	1012.032	0.626
Actinopteri vs. Amphibia	***q=0***	50097.000	2844.000	47253.000	48576.414	51138.716	0.999
	q=1	13506.426	2246.907	11259.519	15287.721	20800.988	1.000
	q=2	2664.671	1650.965	1013.706	1488.688	5628.271	0.994
	q=3	1346.527	1274.886	71.642	-299.080	2756.088	0.974
	q=4	967.127	1077.190	-110.063	-666.080	1939.329	0.916
Amphibia vs. Sauropsida	***q=0***	2844.000	25720.000	-22876.000	-24979.036	-22505.984	0.914
	q=1	2228.301	8442.129	-6213.828	-11170.969	-8485.080	1.000
	q=2	1622.693	2776.517	-1153.824	-3405.069	-1207.233	0.975
	q=3	1248.273	1619.455	-371.182	-1642.222	182.269	0.798
	q=4	1054.034	1174.765	-120.731	-1126.652	435.816	0.877
Sauropsida vs. Aves	***q=0***	25720.000	29298.000	-3578.000	-15293.544	-8900.040	1.000
	q=1	8417.135	11754.945	-3337.810	-8239.438	-2173.804	0.885
	q=2	2766.051	3873.996	-1107.946	-3189.123	1480.223	0.472
	q=3	1612.785	2232.248	-619.464	-1687.641	1252.404	0.444
	q=4	1170.646	1665.395	-494.749	-1240.330	1005.415	0.417
Aves vs. Mammalia	***q=0***	29298.000	207896.000	-178598.000	-121707.354	-99147.786	0.000
	q=1	11580.276	130024.721	-118444.445	-83004.488	-58684.930	0.000
	q=2	3816.430	56081.258	-52264.828	-40155.621	-11512.963	0.000
	q=3	2205.743	27153.470	-24947.727	-18907.633	5617.114	0.003
	q=4	1647.776	17273.362	-15625.586	-11850.213	6170.683	0.003
Percentage of Significant Differences (%)	***q=0***						11.10%
	q=1						11.10%
	q=2						11.10%
	q=3						11.10%
	q=4						11.10%

SDP (Specificity diversity permutation) tests at host class level: from pairwise comparisons between 10 classes (Table S5G-S5L)

Table S5G. SDP (Specificity diversity permutation) tests for the US (unique species) category in the former, from pairwise comparison between 10 host animal classes

Treatments	Order	Former	Latter	Delta	Lower (95%)	Upper (95%)	*p*-Value
Chromadorea vs. Malacostraca	*q=0*	12.000	NA	12.000	-0.394	0.426	0.000
	q=1	11.529	NA	11.529	-1.287	3.532	0.000
	q=2	11.065	NA	11.065	-1.870	4.556	0.000
	q=3	10.632	NA	10.632	-2.242	4.965	0.000
	q=4	10.247	NA	10.247	-2.463	5.119	0.000
Chromadorea vs. Insecta	*q=0*	6867.000	NA	6867.000	-5226.470	-3027.178	0.000
	q=1	3724.917	NA	3724.917	-3458.546	-1619.375	0.001
	q=2	1627.666	NA	1627.666	-1795.997	-184.147	0.049
	q=3	990.297	NA	990.297	-957.152	266.778	0.013
	q=4	749.786	NA	749.786	-660.644	330.124	0.011
Chromadorea vs. Chondrichthyes	*q=0*	20.000	NA	20.000	-0.549	0.603	0.000
	q=1	19.190	NA	19.190	-2.173	5.575	0.000
	q=2	18.417	NA	18.417	-3.075	7.338	0.000
	q=3	17.720	NA	17.720	-3.635	8.114	0.000
	q=4	17.120	NA	17.120	-3.969	8.450	0.000
Chromadorea vs. Actinopteri	*q=0*	7188.000	NA	7188.000	-5894.133	-3748.483	0.000
	q=1	3845.650	NA	3845.650	-3747.217	-2048.837	0.003
	q=2	1655.911	NA	1655.911	-1804.515	-427.321	0.053
	q=3	974.812	NA	974.812	-927.310	121.158	0.005
	q=4	704.626	NA	704.626	-648.121	250.928	0.006
Chromadorea vs. Amphibia	*q=0*	6.000	NA	6.000	-0.145	0.157	0.000
	q=1	5.915	NA	5.915	-0.522	1.400	0.000
	q=2	5.835	NA	5.835	-0.663	1.846	0.000
	q=3	5.761	NA	5.761	-0.751	2.040	0.000
	q=4	5.695	NA	5.695	-0.816	2.131	0.000
Chromadorea vs. Sauropsida	*q=0*	7221.000	NA	7221.000	-2581.395	258.963	0.000
	q=1	3785.580	NA	3785.580	-1864.639	481.199	0.000
	q=2	1559.253	NA	1559.253	-1135.735	665.140	0.003
	q=3	881.362	NA	881.362	-681.422	531.178	0.003
	q=4	619.659	NA	619.659	-525.842	441.341	0.011
Chromadorea vs. Aves	*q=0*	7427.000	NA	7427.000	-4095.590	-1326.222	0.000
	q=1	3632.985	NA	3632.985	-2581.426	-395.507	0.000
	q=2	1442.248	NA	1442.248	-1244.528	386.116	0.004
	q=3	823.702	NA	823.702	-726.358	493.139	0.007
	q=4	592.459	NA	592.459	-556.082	440.247	0.015
Chromadorea vs. Mammalia	*q=0*	7269.000	NA	7269.000	-6162.338	-4150.320	0.000
	q=1	3701.091	NA	3701.091	-3746.375	-2159.737	0.013
	q=2	1525.570	NA	1525.570	-1704.447	-420.947	0.063
	q=3	895.298	NA	895.298	-906.816	134.469	0.014
	q=4	656.852	NA	656.852	-656.295	255.077	0.015
Arachnida vs. Insecta	*q=0*	3569.000	NA	3569.000	-3799.142	-2670.620	0.000
	q=1	2522.691	NA	2522.691	-2738.341	-2164.939	0.300
	q=2	1193.295	NA	1193.295	-1694.803	-1063.811	0.812
	q=3	604.505	NA	604.505	-1014.529	-446.570	0.749

	q=4	410.883	NA	410.883	-672.921	-254.413	0.586
Arachnida vs. Chondrichthyes	***q=0***	82.000	NA	82.000	-2.836	5.282	0.000
	q=1	68.576	NA	68.576	-7.534	12.797	0.000
	q=2	56.701	NA	56.701	-8.682	13.323	0.000
	q=3	48.162	NA	48.162	-8.634	12.352	0.000
	q=4	42.589	NA	42.589	-8.315	11.315	0.000
Arachnida vs. Actinopteri	***q=0***	3584.000	NA	3584.000	-3835.073	-2771.405	0.000
	q=1	2540.132	NA	2540.132	-2755.170	-2246.528	0.369
	q=2	1197.399	NA	1197.399	-1754.987	-1045.835	0.747
	q=3	603.798	NA	603.798	-1059.067	-435.296	0.719
	q=4	410.019	NA	410.019	-702.089	-258.744	0.611
Arachnida vs. Amphibia	***q=0***	40.000	NA	40.000	-1.529	2.273	0.000
	q=1	35.539	NA	35.539	-3.344	7.145	0.000
	q=2	31.648	NA	31.648	-4.639	8.379	0.000
	q=3	28.643	NA	28.643	-5.103	8.401	0.000
	q=4	26.440	NA	26.440	-5.230	8.108	0.000
Arachnida vs. Sauropsida	***q=0***	3578.000	NA	3578.000	-3459.789	-1690.549	0.000
	q=1	2530.283	NA	2530.283	-2631.463	-1399.623	0.000
	q=2	1197.020	NA	1197.020	-1538.006	-847.481	0.518
	q=3	606.100	NA	606.100	-903.758	-270.286	0.422
	q=4	411.851	NA	411.851	-613.568	-30.020	0.231
Arachnida vs. Aves	***q=0***	3638.000	NA	3638.000	-3743.069	-2250.563	0.000
	q=1	2581.345	NA	2581.345	-2788.881	-1856.471	0.061
	q=2	1223.078	NA	1223.078	-1663.015	-1048.297	0.815
	q=3	617.455	NA	617.455	-998.484	-396.984	0.686
	q=4	418.752	NA	418.752	-672.001	-156.723	0.398
Arachnida vs. Mammalia	***q=0***	3606.000	NA	3606.000	-3857.501	-2887.687	0.000
	q=1	2556.806	NA	2556.806	-2765.482	-2306.396	0.657
	q=2	1208.177	NA	1208.177	-1761.307	-1034.599	0.953
	q=3	609.624	NA	609.624	-1052.283	-432.276	0.939
	q=4	413.746	NA	413.746	-697.145	-262.855	0.838
Malacostraca vs. Chondrichthyes	***q=0***	3701.000	NA	3701.000	-1424.102	1210.964	0.000
	q=1	2834.314	NA	2834.314	-1260.460	1089.304	0.000
	q=2	1859.411	NA	1859.411	-1131.938	1012.436	0.000
	q=3	1274.706	NA	1274.706	-1081.327	1008.838	0.006
	q=4	1003.219	NA	1003.219	-1015.760	973.564	0.026
Malacostraca vs. Actinopteri	***q=0***	3571.000	NA	3571.000	-3902.711	-2874.065	0.000
	q=1	2867.564	NA	2867.564	-3182.009	-2308.814	0.061
	q=2	1977.005	NA	1977.005	-2387.386	-1489.545	0.193
	q=3	1353.408	NA	1353.408	-1783.325	-893.828	0.206
	q=4	1042.423	NA	1042.423	-1393.240	-635.491	0.197
Malacostraca vs. Amphibia	***q=0***	110.000	NA	110.000	-9.912	14.094	0.000
	q=1	105.400	NA	105.400	-30.070	39.207	0.000
	q=2	101.685	NA	101.685	-41.967	52.349	0.000
	q=3	98.660	NA	98.660	-48.918	59.568	0.000
	q=4	96.156	NA	96.156	-53.036	63.664	0.000
Malacostraca vs. Sauropsida	***q=0***	3629.000	NA	3629.000	-3878.099	-1983.781	0.000
	q=1	2899.445	NA	2899.445	-3214.017	-1583.929	0.028
	q=2	1993.892	NA	1993.892	-2493.512	-998.103	0.233
	q=3	1369.846	NA	1369.846	-1927.838	-500.966	0.332
	q=4	1059.145	NA	1059.145	-1530.951	-252.588	0.302

Malacostraca vs. Aves	***q=0***	3699.000	NA	3699.000	-4014.318	-2442.550	0.000
	q=1	2846.905	NA	2846.905	-3198.780	-1897.400	0.061
	q=2	1875.213	NA	1875.213	-2414.287	-1115.046	0.326
	q=3	1284.141	NA	1284.141	-1818.645	-602.618	0.342
	q=4	1008.388	NA	1008.388	-1426.732	-384.333	0.303
Malacostraca vs. Mammalia	***q=0***	3509.000	NA	3509.000	-3824.994	-2890.638	0.000
	q=1	2736.193	NA	2736.193	-3024.794	-2260.830	0.628
	q=2	1816.229	NA	1816.229	-2212.563	-1375.410	0.727
	q=3	1235.377	NA	1235.377	-1629.319	-823.888	0.730
	q=4	962.212	NA	962.212	-1272.205	-606.587	0.716
Insecta vs. Actinopteri	***q=0***	35467.000	NA	35467.000	-9614.946	809.812	0.000
	q=1	17106.004	NA	17106.004	-7231.135	2091.312	0.000
	q=2	4727.222	NA	4727.222	-4025.775	2911.644	0.007
	q=3	2170.789	NA	2170.789	-2148.324	1916.919	0.037
	q=4	1472.960	NA	1472.960	-1391.197	1261.793	0.030
Insecta vs. Amphibia	***q=0***	31304.000	NA	31304.000	28586.398	31420.370	0.000
	q=1	30289.612	NA	30289.612	27734.151	30687.803	0.008
	q=2	19738.444	NA	19738.444	18355.047	30023.266	0.847
	q=3	3648.964	NA	3648.964	-89.264	17626.916	0.836
	q=4	1477.178	NA	1477.178	-901.837	7036.699	0.774
Insecta vs. Sauropsida	***q=0***	322.000	NA	322.000	-2.994	4.404	0.000
	q=1	264.086	NA	264.086	-25.401	69.464	0.000
	q=2	214.335	NA	214.335	-39.420	83.389	0.000
	q=3	180.271	NA	180.271	-43.051	82.034	0.000
	q=4	159.110	NA	159.110	-42.242	77.217	0.000
Insecta vs. Aves	***q=0***	996.000	NA	996.000	-36.733	53.705	0.000
	q=1	738.032	NA	738.032	-187.087	293.461	0.000
	q=2	512.918	NA	512.918	-220.206	300.402	0.000
	q=3	380.967	NA	380.967	-195.419	252.269	0.000
	q=4	313.097	NA	313.097	-166.133	211.434	0.000
Insecta vs. Mammalia	***q=0***	35835.000	NA	35835.000	-12247.088	-1795.476	0.000
	q=1	17500.474	NA	17500.474	-8803.599	573.461	0.000
	q=2	4918.365	NA	4918.365	-4544.330	2710.808	0.012
	q=3	2240.374	NA	2240.374	-2402.994	2036.528	0.045
	q=4	1508.583	NA	1508.583	-1567.685	1364.543	0.040
Chondrichthyes vs. Amphibia	***q=0***	30.000	NA	30.000	-1.076	1.880	0.000
	q=1	27.515	NA	27.515	-4.293	7.690	0.000
	q=2	24.948	NA	24.948	-5.401	8.880	0.000
	q=3	22.435	NA	22.435	-5.713	9.047	0.000
	q=4	20.216	NA	20.216	-5.736	8.904	0.000
Chondrichthyes vs. Sauropsida	***q=0***	2708.000	NA	2708.000	-3085.515	-1294.129	0.000
	q=1	2211.914	NA	2211.914	-2717.719	-887.590	0.125
	q=2	1456.596	NA	1456.596	-2134.255	-448.305	0.478
	q=3	836.291	NA	836.291	-1563.643	-40.619	0.577
	q=4	544.401	NA	544.401	-1184.716	167.093	0.569
Chondrichthyes vs. Aves	***q=0***	2627.000	NA	2627.000	-2998.736	-1596.450	0.000
	q=1	2144.933	NA	2144.933	-2623.061	-1167.410	0.192
	q=2	1409.270	NA	1409.270	-2022.277	-672.515	0.535
	q=3	806.534	NA	806.534	-1462.537	-215.000	0.609
	q=4	524.632	NA	524.632	-1102.399	9.874	0.570
Chondrichthyes vs.	***q=0***	1666.000	NA	1666.000	-1876.119	-1314.507	0.000

Mammalia	***q=1***	1302.814	NA	1302.814	-1527.176	-996.768	0.154
	q=2	833.549	NA	833.549	-1073.165	-587.186	0.285
	q=3	523.478	NA	523.478	-741.594	-304.469	0.264
	q=4	381.276	NA	381.276	-568.948	-176.955	0.248
Actinopteri vs. Sauropsida	***q=0***	727.000	NA	727.000	-4.262	6.518	0.000
	q=1	533.666	NA	533.666	-60.335	127.244	0.000
	q=2	385.406	NA	385.406	-91.558	141.812	0.000
	q=3	300.549	NA	300.549	-90.972	125.204	0.000
	q=4	254.515	NA	254.515	-81.943	108.248	0.000
Actinopteri vs. Aves	***q=0***	1345.000	NA	1345.000	-10.682	17.558	0.000
	q=1	883.473	NA	883.473	-118.455	208.244	0.000
	q=2	563.281	NA	563.281	-160.202	209.855	0.000
	q=3	409.193	NA	409.193	-143.073	170.224	0.000
	q=4	335.316	NA	335.316	-121.149	140.036	0.000
Actinopteri vs. Mammalia	***q=0***	49149.000	NA	49149.000	-7924.209	495.475	0.000
	q=1	13472.174	NA	13472.174	-4777.065	2227.400	0.000
	q=2	2689.577	NA	2689.577	-2184.269	2051.534	0.011
	q=3	1356.908	NA	1356.908	-1066.755	1047.222	0.010
	q=4	973.719	NA	973.719	-703.587	689.630	0.006
Amphibia vs. Aves	***q=0***	2839.000	NA	2839.000	-3204.103	-1944.803	0.000
	q=1	2227.571	NA	2227.571	-2483.168	-1613.371	0.000
	q=2	1623.187	NA	1623.187	-1860.025	-1200.503	0.135
	q=3	1248.809	NA	1248.809	-1474.087	-878.564	0.182
	q=4	1054.476	NA	1054.476	-1235.056	-696.459	0.099
Amphibia vs. Mammalia	***q=0***	489.000	NA	489.000	-598.218	-334.064	0.000
	q=1	436.994	NA	436.994	-604.288	-198.579	0.000
	q=2	394.176	NA	394.176	-562.285	-155.264	0.000
	q=3	362.615	NA	362.615	-527.502	-128.683	0.000
	q=4	340.392	NA	340.392	-503.436	-109.386	0.000
Sauropsida vs. Mammalia	***q=0***	25081.000	NA	25081.000	-17801.753	-12905.035	0.000
	q=1	8560.932	NA	8560.932	-7610.764	-5256.848	0.000
	q=2	2833.551	NA	2833.551	-2234.967	-181.323	0.001
	q=3	1666.759	NA	1666.759	-1097.584	454.595	0.000
	q=4	1219.367	NA	1219.367	-779.931	444.618	0.000
Percentage of Significant Differences (%)	***q=0***						100%
	q=1						72.20%
	q=2						55.60%
	q=3						61.10%
	q=4						61.10%

Table S5H. SDP (Specificity diversity permutation) tests for the US (unique species) category in the latter class, from pairwise comparison between 10 host animal classes

Treatments	Order	Former	Latter	Delta	Lower (95%)	Upper (95%)	*p*-Value
Chromadorea vs. Malacostraca	***q=0***	NA	3551.000	-3551.000	1628.924	3672.524	0.000
	q=1	NA	2854.152	-2854.152	1295.595	3078.899	0.004
	q=2	NA	1974.532	-1974.532	810.978	2398.420	0.160
	q=3	NA	1356.118	-1356.118	361.085	1850.765	0.268
	q=4	NA	1045.734	-1045.734	118.552	1470.249	0.237
Chromadorea vs.	***q=0***	NA	110.000	-110.000	-0.719	0.649	0.000

Insecta	***q=1***	NA	99.545	-99.545	-17.088	4.934	0.000
	q=2	NA	90.752	-90.752	-23.871	8.575	0.000
	q=3	NA	83.999	-83.999	-26.911	10.803	0.000
	q=4	NA	79.018	-79.018	-28.222	12.001	0.000
Chromadorea vs. Chondrichthyes	***q=0***	NA	2711.000	-2711.000	942.947	3030.695	0.000
	q=1	NA	2214.602	-2214.602	572.273	2692.002	0.066
	q=2	NA	1458.452	-1458.452	188.395	2164.274	0.395
	q=3	NA	837.263	-837.263	-162.280	1616.402	0.524
	q=4	NA	544.972	-544.972	-334.327	1230.027	0.536
Chromadorea vs. Actinopteri	***q=0***	NA	325.000	-325.000	-1.034	0.908	0.000
	q=1	NA	267.689	-267.689	-47.547	20.762	0.000
	q=2	NA	219.913	-219.913	-59.952	33.723	0.000
	q=3	NA	187.065	-187.065	-59.488	37.681	0.000
	q=4	NA	166.022	-166.022	-55.917	37.361	0.000
Chromadorea vs. Amphibia	***q=0***	NA	2798.000	-2798.000	1371.419	3149.967	0.000
	q=1	NA	2191.961	-2191.961	1172.256	2474.603	0.000
	q=2	NA	1594.676	-1594.676	891.310	1889.152	0.149
	q=3	NA	1226.607	-1226.607	629.604	1518.013	0.214
	q=4	NA	1036.209	-1036.209	461.919	1277.161	0.146
Chromadorea vs. Sauropsida	***q=0***	NA	1518.000	-1518.000	-11.079	3.279	0.000
	q=1	NA	1166.038	-1166.038	-137.714	58.271	0.000
	q=2	NA	864.258	-864.258	-163.540	94.142	0.000
	q=3	NA	652.365	-652.365	-160.241	108.297	0.000
	q=4	NA	519.462	-519.462	-149.406	110.179	0.000
Chromadorea vs. Aves	***q=0***	NA	704.000	-704.000	-3.190	1.638	0.000
	q=1	NA	604.895	-604.895	-67.006	4.702	0.000
	q=2	NA	516.029	-516.029	-85.586	18.799	0.000
	q=3	NA	446.060	-446.060	-92.193	32.070	0.000
	q=4	NA	394.809	-394.809	-94.521	42.033	0.000
Chromadorea vs. Mammalia	***q=0***	NA	15.000	-15.000	0.000	0.000	0.000
	q=1	NA	14.781	-14.781	-2.289	0.935	0.000
	q=2	NA	14.579	-14.579	-3.577	1.620	0.000
	q=3	NA	14.395	-14.395	-4.362	2.147	0.000
	q=4	NA	14.230	-14.230	-4.841	2.539	0.000
Arachnida vs. Insecta	***q=0***	NA	NA	NA	NA	NA	NA
	q=1	NA	NA	NA	NA	NA	NA
	q=2	NA	NA	NA	NA	NA	NA
	q=3	NA	NA	NA	NA	NA	NA
	q=4	NA	NA	NA	NA	NA	NA
Arachnida vs. Chondrichthyes	***q=0***	NA	2709.000	-2709.000	-1025.552	1918.434	0.000
	q=1	NA	2219.241	-2219.241	-1037.061	1753.528	0.000
	q=2	NA	1465.367	-1465.367	-1031.075	1535.055	0.030
	q=3	NA	838.308	-838.308	-1009.517	1291.847	0.185
	q=4	NA	543.592	-543.592	-931.240	1071.190	0.315
Arachnida vs. Actinopteri	***q=0***	NA	26.000	-26.000	-0.090	0.086	0.000
	q=1	NA	25.660	-25.660	-1.932	0.844	0.000
	q=2	NA	25.335	-25.335	-3.158	1.342	0.000
	q=3	NA	25.029	-25.029	-4.035	1.709	0.000
	q=4	NA	24.742	-24.742	-4.690	1.996	0.000
Arachnida vs. Amphibia	***q=0***	NA	2842.000	-2842.000	-207.481	2124.845	0.000
	q=1	NA	2229.986	-2229.986	-188.755	1795.657	0.000

	q=2	NA	1625.477	-1625.477	-197.397	1485.883	0.000
	q=3	NA	1250.966	-1250.966	-261.946	1266.146	0.011
	q=4	NA	1056.440	-1056.440	-324.949	1106.137	0.023
Arachnida vs. Sauropsida	***q=0***	NA	134.000	-134.000	-0.488	0.412	0.000
	q=1	NA	125.835	-125.835	-15.884	0.135	0.000
	q=2	NA	117.169	-117.169	-22.299	1.691	0.000
	q=3	NA	108.472	-108.472	-25.696	4.367	0.000
	q=4	NA	100.358	-100.358	-27.735	7.380	0.000
Arachnida vs. Aves	***q=0***	NA	27.000	-27.000	-0.063	0.061	0.000
	q=1	NA	26.639	-26.639	-5.194	1.254	0.000
	q=2	NA	26.260	-26.260	-7.621	2.057	0.000
	q=3	NA	25.867	-25.867	-9.059	2.910	0.000
	q=4	NA	25.468	-25.468	-9.954	3.708	0.000
Arachnida vs. Mammalia	***q=0***	NA	NA	NA	NA	NA	NA
	q=1	NA	NA	NA	NA	NA	NA
	q=2	NA	NA	NA	NA	NA	NA
	q=3	NA	NA	NA	NA	NA	NA
	q=4	NA	NA	NA	NA	NA	NA
Malacostraca vs. Chondrichthyes	***q=0***	NA	44.000	-44.000	-2.984	2.504	0.000
	q=1	NA	41.804	-41.804	-12.035	10.743	0.000
	q=2	NA	39.654	-39.654	-13.840	12.482	0.000
	q=3	NA	37.651	-37.651	-14.344	12.962	0.000
	q=4	NA	35.858	-35.858	-14.424	13.035	0.000
Malacostraca vs. Actinopteri	***q=0***	NA	42000.000	-42000.000	-41131.524	-38815.950	0.000
	q=1	NA	12766.553	-12766.553	-25068.166	-15494.471	1.000
	q=2	NA	888.655	-888.655	-2819.111	122.419	0.718
	q=3	NA	454.455	-454.455	-1034.876	723.484	0.358
	q=4	NA	352.747	-352.747	-771.018	770.514	0.348
Malacostraca vs. Amphibia	***q=0***	NA	2827.000	-2827.000	-693.345	1654.985	0.000
	q=1	NA	2217.112	-2217.112	-617.953	1427.788	0.000
	q=2	NA	1615.492	-1615.492	-556.458	1210.690	0.000
	q=3	NA	1243.361	-1243.361	-547.057	1061.135	0.005
	q=4	NA	1050.243	-1050.243	-551.615	954.736	0.011
Malacostraca vs. Sauropsida	***q=0***	NA	58.000	-58.000	-0.253	0.223	0.000
	q=1	NA	55.213	-55.213	-8.243	1.868	0.000
	q=2	NA	52.322	-52.322	-12.197	3.720	0.000
	q=3	NA	49.488	-49.488	-14.402	5.525	0.000
	q=4	NA	46.874	-46.874	-15.602	7.023	0.000
Malacostraca vs. Aves	***q=0***	NA	4.000	-4.000	-0.063	0.061	0.000
	q=1	NA	3.986	-3.986	-0.785	0.390	0.000
	q=2	NA	3.973	-3.973	-1.052	0.502	0.000
	q=3	NA	3.959	-3.959	-1.172	0.558	0.000
	q=4	NA	3.947	-3.947	-1.238	0.595	0.000
Malacostraca vs. Mammalia	***q=0***	NA	178878.000	-178878.000	-175087.020	-168822.708	0.000
	q=1	NA	173026.082	-173026.082	-169086.042	-162473.169	0.000
	q=2	NA	163461.798	-163461.798	-159996.393	-153426.385	0.000
	q=3	NA	133729.788	-133729.788	-138428.540	-122467.017	0.197
	q=4	NA	69148.858	-69148.858	-86228.675	-53466.800	0.373
Insecta vs.	***q=0***	NA	3265.000	-3265.000	-68.085	2.329	0.000

Actinopteri	***q=1***	NA	1712.403	-1712.403	-403.819	325.203	0.000
	q=2	NA	875.987	-875.987	-357.196	328.329	0.000
	q=3	NA	578.582	-578.582	-259.881	245.593	0.000
	q=4	NA	456.398	-456.398	-201.385	191.052	0.000
Insecta vs. Amphibia	***q=0***	NA	2752.000	-2752.000	2129.319	3097.407	0.000
	q=1	NA	2151.609	-2151.609	1727.169	2387.337	0.000
	q=2	NA	1560.351	-1560.351	1280.839	1745.378	0.087
	q=3	NA	1198.596	-1198.596	955.464	1365.761	0.119
	q=4	NA	1012.735	-1012.735	770.965	1156.131	0.030
Insecta vs. Sauropsida	***q=0***	NA	25076.000	-25076.000	9362.544	14833.010	0.000
	q=1	NA	9073.795	-9073.795	3995.571	6786.139	0.000
	q=2	NA	3020.922	-3020.922	-217.816	2253.370	0.001
	q=3	NA	1748.249	-1748.249	-642.855	1168.945	0.001
	q=4	NA	1258.551	-1258.551	-548.948	831.701	0.001
Insecta vs. Aves	***q=0***	NA	29180.000	-29180.000	5453.818	11731.568	0.000
	q=1	NA	11728.749	-11728.749	1347.972	6780.250	0.000
	q=2	NA	3866.980	-3866.980	-1334.737	2872.044	0.000
	q=3	NA	2228.616	-2228.616	-1095.947	1446.926	0.000
	q=4	NA	1662.877	-1662.877	-782.361	978.663	0.000
Insecta vs. Mammalia	***q=0***	NA	3337.000	-3337.000	-27.110	12.664	0.000
	q=1	NA	2879.568	-2879.568	-399.314	154.896	0.000
	q=2	NA	2384.045	-2384.045	-516.078	273.646	0.000
	q=3	NA	1945.026	-1945.026	-524.791	322.914	0.000
	q=4	NA	1621.886	-1621.886	-489.185	325.184	0.000
Chondrichthyes vs. Amphibia	***q=0***	NA	2844.000	-2844.000	-559.729	1720.797	0.000
	q=1	NA	2231.706	-2231.706	-503.378	1480.040	0.000
	q=2	NA	1626.706	-1626.706	-466.734	1250.306	0.000
	q=3	NA	1251.816	-1251.816	-484.220	1093.280	0.001
	q=4	NA	1057.095	-1057.095	-508.034	980.063	0.008
Chondrichthyes vs. Sauropsida	***q=0***	NA	59.000	-59.000	-0.157	0.145	0.000
	q=1	NA	56.232	-56.232	-7.665	2.055	0.000
	q=2	NA	53.350	-53.350	-11.433	3.926	0.000
	q=3	NA	50.527	-50.527	-13.502	5.664	0.000
	q=4	NA	47.936	-47.936	-14.583	7.070	0.000
Chondrichthyes vs. Aves	***q=0***	NA	1.000	-1.000	0.000	0.000	0.000
	q=1	NA	1.000	-1.000	0.000	0.000	0.000
	q=2	NA	1.000	-1.000	0.000	0.000	0.000
	q=3	NA	1.000	-1.000	0.000	0.000	0.000
	q=4	NA	1.000	-1.000	0.000	0.000	0.000
Chondrichthyes vs. Mammalia	***q=0***	NA	173578.000	-173578.000	-169566.986	-163032.864	0.000
	q=1	NA	169116.733	-169116.733	-165002.990	-158251.648	0.000
	q=2	NA	160991.610	-160991.610	-157394.772	-150617.234	0.000
	q=3	NA	130231.368	-130231.368	-136098.749	-118217.093	0.229
	q=4	NA	64764.057	-64764.057	-82308.911	-48548.149	0.374
Actinopteri vs. Sauropsida	***q=0***	NA	25522.000	-25522.000	11687.525	16942.251	0.000
	q=1	NA	8596.245	-8596.245	4587.660	7188.103	0.000
	q=2	NA	2865.213	-2865.213	-23.563	2137.272	0.000
	q=3	NA	1691.047	-1691.047	-510.169	1072.286	0.000
	q=4	NA	1238.462	-1238.462	-465.125	763.893	0.000

Actinopteri vs. Aves	*q=0*	NA	29249.000	-29249.000	8626.907	14852.011	0.000
	q=1	NA	11764.882	-11764.882	2935.159	8316.308	0.000
	q=2	NA	3878.002	-3878.002	-931.153	3252.135	0.006
	q=3	NA	2234.120	-2234.120	-991.639	1589.804	0.000
	q=4	NA	1666.639	-1666.639	-733.089	1076.689	0.000
Actinopteri vs. Mammalia	*q=0*	NA	7818.000	-7818.000	-129.335	43.883	0.000
	q=1	NA	6434.395	-6434.395	-811.110	524.153	0.000
	q=2	NA	4944.060	-4944.060	-1058.194	815.987	0.000
	q=3	NA	3725.925	-3725.925	-1086.258	913.058	0.000
	q=4	NA	2934.350	-2934.350	-993.627	871.639	0.000
Amphibia vs. Aves	*q=0*	NA	NA	NA	NA	NA	NA
	q=1	NA	NA	NA	NA	NA	NA
	q=2	NA	NA	NA	NA	NA	NA
	q=3	NA	NA	NA	NA	NA	NA
	q=4	NA	NA	NA	NA	NA	NA
Amphibia vs. Mammalia	*q=0*	NA	NA	NA	NA	NA	NA
	q=1	NA	NA	NA	NA	NA	NA
	q=2	NA	NA	NA	NA	NA	NA
	q=3	NA	NA	NA	NA	NA	NA
	q=4	NA	NA	NA	NA	NA	NA
Sauropsida vs. Mammalia	*q=0*	NA	135.000	-135.000	-0.351	0.313	0.000
	q=1	NA	130.515	-130.515	-21.268	5.492	0.000
	q=2	NA	125.987	-125.987	-31.927	10.290	0.000
	q=3	NA	121.527	-121.527	-38.038	14.716	0.000
	q=4	NA	117.256	-117.256	-41.504	18.480	0.000
Percentage of Significant Differences (%)	*q=0*						100%
	q=1						94.40%
	q=2						86.10%
	q=3						77.80%
	q=4						80.60%

Table S5I. SDP (Specificity diversity permutation) tests for the ES (enriched species) category in the former, from pairwise comparison between 10 host animal classes

Treatments	Order	Former	Latter	Delta	Lower (95%)	Upper (95%)	*p*-Value
Chromadorea vs. Malacostraca	*q=0*	5.000	5.000	0.000	-0.061	0.063	0.001
	q=1	4.881	1.787	3.094	-0.684	0.998	0.002
	q=2	4.781	1.407	3.374	-0.945	1.396	0.002
	q=3	4.698	1.312	3.386	-1.080	1.601	0.001
	q=4	4.631	1.275	3.356	-1.154	1.711	0.001
Chromadorea vs. Insecta	*q=0*	199.000	199.000	0.000	-65.415	-17.009	1.000
	q=1	119.612	13.439	106.173	-72.438	1.229	0.000
	q=2	76.581	5.374	71.206	-59.192	9.582	0.001
	q=3	57.965	4.281	53.684	-49.127	11.973	0.005
	q=4	49.236	3.922	45.314	-42.733	12.382	0.008
Chromadorea vs. Chondrichthyes	*q=0*	NA	NA	NA	NA	NA	NA
	q=1	NA	NA	NA	NA	NA	NA
	q=2	NA	NA	NA	NA	NA	NA
	q=3	NA	NA	NA	NA	NA	NA
	q=4	NA	NA	NA	NA	NA	NA

Chromadorea vs. Actinopteri	***q=0***	121.000	121.000	0.000	-45.057	-11.607	1.000
	q=1	73.439	19.882	53.557	-45.358	-4.911	0.004
	q=2	50.372	10.869	39.503	-38.807	0.559	0.013
	q=3	40.179	8.283	31.896	-34.156	3.492	0.042
	q=4	35.078	7.078	28.000	-31.118	5.020	0.050
Chromadorea vs. Amphibia	***q=0***	2.000	2.000	0.000	-0.061	0.063	0.001
	q=1	2.000	1.512	0.488	-0.186	0.296	0.016
	q=2	1.999	1.329	0.670	-0.227	0.387	0.013
	q=3	1.999	1.261	0.738	-0.243	0.428	0.011
	q=4	1.998	1.231	0.767	-0.251	0.450	0.008
Chromadorea vs. Sauropsida	***q=0***	57.000	57.000	0.000	-1.318	0.860	0.221
	q=1	41.851	7.779	34.072	-12.578	9.079	0.000
	q=2	32.311	3.718	28.592	-13.236	10.196	0.000
	q=3	27.152	2.904	24.248	-12.753	10.273	0.000
	q=4	24.242	2.606	21.636	-12.258	10.181	0.000
Chromadorea vs. Aves	***q=0***	15.000	15.000	0.000	-2.441	1.133	0.442
	q=1	10.344	2.104	8.240	-5.655	3.153	0.000
	q=2	7.491	1.624	5.868	-5.986	3.889	0.026
	q=3	5.998	1.498	4.501	-5.822	4.060	0.096
	q=4	5.218	1.441	3.777	-5.585	4.034	0.150
Chromadorea vs. Mammalia	***q=0***	66.000	66.000	0.000	-26.236	-3.942	1.000
	q=1	42.934	5.317	37.617	-32.334	-2.772	0.002
	q=2	28.513	3.447	25.067	-28.728	1.590	0.056
	q=3	21.828	3.069	18.759	-25.111	3.665	0.126
	q=4	18.737	2.912	15.825	-22.518	4.426	0.147
Arachnida vs. Insecta	***q=0***	17.000	17.000	0.000	-20.622	-9.360	1.000
	q=1	15.716	9.098	6.617	-20.411	-4.250	0.919
	q=2	14.209	6.455	7.754	-16.946	-3.609	0.876
	q=3	12.741	5.178	7.563	-14.090	-2.938	0.832
	q=4	11.539	4.536	7.003	-12.416	-2.306	0.692
Arachnida vs. Chondrichthyes	***q=0***	NA	NA	NA	NA	NA	NA
	q=1	NA	NA	NA	NA	NA	NA
	q=2	NA	NA	NA	NA	NA	NA
	q=3	NA	NA	NA	NA	NA	NA
	q=4	NA	NA	NA	NA	NA	NA
Arachnida vs. Actinopteri	***q=0***	24.000	24.000	0.000	-26.385	-16.063	1.000
	q=1	17.393	9.137	8.256	-22.876	-8.933	0.942
	q=2	12.896	5.315	7.581	-18.611	-5.943	0.905
	q=3	10.664	4.203	6.461	-15.839	-3.999	0.875
	q=4	9.539	3.727	5.813	-14.206	-2.852	0.833
Arachnida vs. Amphibia	***q=0***	NA	NA	0.000	0.000	0.000	0.000
	q=1	NA	NA	0.000	0.000	0.000	0.000
	q=2	NA	NA	0.000	0.000	0.000	0.000
	q=3	NA	NA	0.000	0.000	0.000	0.000
	q=4	NA	NA	0.000	0.000	0.000	0.000
Arachnida vs. Sauropsida	***q=0***	1.000	1.000	0.000	-1.593	0.315	0.645
	q=1	1.000	1.000	0.000	-1.593	0.315	0.645
	q=2	1.000	1.000	0.000	-1.593	0.315	0.645
	q=3	1.000	1.000	0.000	-1.593	0.315	0.645
	q=4	1.000	1.000	0.000	-1.593	0.315	0.645
Arachnida vs. Aves	***q=0***	1.000	1.000	0.000	-1.588	-0.018	0.805

	q=1	1.000	1.000	0.000	-1.588	-0.018	0.805
	q=2	1.000	1.000	0.000	-1.588	-0.018	0.805
	q=3	1.000	1.000	0.000	-1.588	-0.018	0.805
	q=4	1.000	1.000	0.000	-1.588	-0.018	0.805
Arachnida vs. Mammalia	***q=0***	6.000	6.000	0.000	-6.777	-3.947	1.000
	q=1	4.237	2.602	1.634	-6.111	-2.264	0.966
	q=2	3.310	2.281	1.029	-5.316	-1.633	0.973
	q=3	2.883	2.199	0.684	-4.786	-1.277	0.975
	q=4	2.665	2.162	0.502	-4.448	-1.075	0.976
Malacostraca vs. Chondrichthyes	***q=0***	NA	NA	NA	NA	NA	NA
	q=1	NA	NA	NA	NA	NA	NA
	q=2	NA	NA	NA	NA	NA	NA
	q=3	NA	NA	NA	NA	NA	NA
	q=4	NA	NA	NA	NA	NA	NA
Malacostraca vs. Actinopteri	***q=0***	136.000	136.000	0.000	-155.220	-82.356	1.000
	q=1	86.308	51.563	34.745	-109.765	-48.902	0.989
	q=2	66.107	34.603	31.504	-84.332	-29.282	0.934
	q=3	57.754	27.610	30.143	-71.969	-16.446	0.843
	q=4	53.427	23.842	29.584	-64.491	-9.019	0.794
Malacostraca vs. Amphibia	***q=0***	2.000	2.000	0.000	-0.122	0.126	0.001
	q=1	1.997	1.862	0.135	-0.391	0.480	0.291
	q=2	1.994	1.755	0.239	-0.467	0.574	0.269
	q=3	1.991	1.679	0.312	-0.492	0.602	0.226
	q=4	1.988	1.627	0.361	-0.500	0.611	0.195
Malacostraca vs. Sauropsida	***q=0***	42.000	42.000	0.000	-38.646	0.772	1.000
	q=1	34.759	5.057	29.702	-37.216	10.661	0.049
	q=2	31.649	3.193	28.456	-34.274	17.020	0.005
	q=3	29.990	2.877	27.113	-30.475	18.694	0.000
	q=4	28.937	2.765	26.172	-27.354	18.498	0.000
Malacostraca vs. Aves	***q=0***	20.000	20.000	0.000	-21.805	-8.681	1.000
	q=1	13.875	8.905	4.970	-18.602	-3.782	0.944
	q=2	10.335	6.355	3.980	-15.882	-2.176	0.923
	q=3	8.612	5.606	3.006	-14.082	-1.256	0.918
	q=4	7.725	5.273	2.452	-12.912	-0.705	0.923
Malacostraca vs. Mammalia	***q=0***	180.000	180.000	0.000	-190.368	-135.282	1.000
	q=1	120.175	31.366	88.809	-144.335	-73.985	0.889
	q=2	82.671	13.835	68.836	-106.222	-41.797	0.726
	q=3	64.846	9.391	55.456	-84.305	-25.064	0.600
	q=4	56.074	7.690	48.384	-71.991	-16.775	0.516
Insecta vs. Actinopteri	***q=0***	80.000	80.000	0.000	-1.579	1.093	0.243
	q=1	58.532	12.117	46.415	-20.286	15.029	0.000
	q=2	36.523	4.563	31.960	-25.431	21.267	0.004
	q=3	23.489	3.470	20.019	-23.367	20.666	0.074
	q=4	17.766	3.092	14.674	-20.003	18.111	0.137
Insecta vs. Amphibia	***q=0***	NA	NA	NA	NA	NA	NA
	q=1	NA	NA	NA	NA	NA	NA
	q=2	NA	NA	NA	NA	NA	NA
	q=3	NA	NA	NA	NA	NA	NA
	q=4	NA	NA	NA	NA	NA	NA
Insecta vs. Sauropsida	***q=0***	7.000	7.000	0.000	0.000	0.000	0.000
	q=1	5.699	5.120	0.579	-1.852	2.284	0.594

	q=2	4.662	4.471	0.191	-2.724	3.096	0.906
	q=3	3.999	4.176	-0.177	-3.001	3.277	0.928
	q=4	3.609	4.003	-0.394	-3.034	3.249	0.829
Insecta vs. Aves	***q=0***	6.000	6.000	0.000	0.000	0.000	0.000
	q=1	4.366	2.175	2.191	-1.780	2.199	0.029
	q=2	3.495	1.706	1.788	-1.806	2.131	0.083
	q=3	3.098	1.568	1.530	-1.622	1.876	0.090
	q=4	2.896	1.504	1.392	-1.481	1.694	0.090
Insecta vs. Mammalia	***q=0***	54.000	54.000	0.000	-2.257	1.135	0.417
	q=1	34.592	12.650	21.942	-13.223	9.503	0.000
	q=2	23.592	6.512	17.081	-14.026	11.447	0.008
	q=3	18.368	4.821	13.547	-12.670	10.765	0.027
	q=4	15.687	4.151	11.536	-11.277	9.716	0.040
Chondrichthyes vs. Amphibia	***q=0***	NA	NA	NA	NA	NA	NA
	q=1	NA	NA	NA	NA	NA	NA
	q=2	NA	NA	NA	NA	NA	NA
	q=3	NA	NA	NA	NA	NA	NA
	q=4	NA	NA	NA	NA	NA	NA
Chondrichthyes vs. Sauropsida	***q=0***	NA	NA	NA	NA	NA	NA
	q=1	NA	NA	NA	NA	NA	NA
	q=2	NA	NA	NA	NA	NA	NA
	q=3	NA	NA	NA	NA	NA	NA
	q=4	NA	NA	NA	NA	NA	NA
Chondrichthyes vs. Aves	***q=0***	32.000	32.000	0.000	-43.856	-9.450	1.000
	q=1	24.389	15.192	9.197	-49.127	8.190	1.000
	q=2	17.198	7.208	9.990	-44.072	10.959	1.000
	q=3	12.653	4.932	7.721	-39.331	12.441	1.000
	q=4	10.306	4.164	6.142	-36.018	13.230	1.000
Chondrichthyes vs. Mammalia	***q=0***	849.000	849.000	0.000	-979.013	-604.223	1.000
	q=1	727.018	183.462	543.556	-917.060	-323.093	0.887
	q=2	484.133	76.365	407.768	-742.090	-177.393	0.883
	q=3	227.935	51.413	176.523	-582.826	-69.559	0.899
	q=4	133.779	41.534	92.245	-473.994	-8.390	0.955
Actinopteri vs. Sauropsida	***q=0***	1.000	1.000	0.000	-0.133	0.143	0.005
	q=1	1.000	1.000	0.000	-0.133	0.143	0.005
	q=2	1.000	1.000	0.000	-0.133	0.143	0.005
	q=3	1.000	1.000	0.000	-0.133	0.143	0.005
	q=4	1.000	1.000	0.000	-0.133	0.143	0.005
Actinopteri vs. Aves	***q=0***	3.000	3.000	0.000	-0.120	0.128	0.004
	q=1	2.973	2.236	0.737	-0.438	0.808	0.071
	q=2	2.948	1.886	1.062	-0.535	0.990	0.028
	q=3	2.926	1.728	1.198	-0.589	1.069	0.020
	q=4	2.906	1.647	1.259	-0.623	1.107	0.015
Actinopteri vs. Mammalia	***q=0***	33.000	33.000	0.000	-0.437	0.391	0.039
	q=1	29.260	4.934	24.326	-6.587	5.936	0.000
	q=2	26.215	2.323	23.891	-8.624	7.981	0.000
	q=3	23.907	1.926	21.980	-9.369	8.803	0.000
	q=4	22.204	1.794	20.410	-9.628	9.136	0.000
Amphibia vs. Aves	***q=0***	5.000	5.000	0.000	-6.177	-2.845	1.000
	q=1	4.160	1.716	2.444	-6.343	-1.581	0.927
	q=2	3.513	1.440	2.074	-5.930	-1.315	0.929

	q=3	3.110	1.351	1.758	-5.536	-1.164	0.929
	q=4	2.872	1.311	1.560	-5.225	-1.061	0.929
Amphibia vs. Mammalia	*q=0*	46.000	46.000	0.000	-54.713	-27.561	1.000
	q=1	38.994	6.831	32.162	-47.956	-11.678	0.591
	q=2	34.843	4.353	30.489	-41.186	-4.308	0.000
	q=3	32.404	3.667	28.737	-36.043	-0.807	0.000
	q=4	30.887	3.356	27.531	-32.422	0.855	0.000
Sauropsida vs. Mammalia	*q=0*	171.000	171.000	0.000	-34.426	-11.504	1.000
	q=1	97.346	29.085	68.261	-35.631	2.060	0.000
	q=2	62.396	16.526	45.870	-29.781	8.806	0.000
	q=3	46.408	13.284	33.123	-24.912	10.593	0.003
	q=4	38.534	11.868	26.667	-21.538	10.542	0.004
Percentage of Significant Differences (%)	*q=0*						41.70%
	q=1						58.30%
	q=2						58.30%
	q=3						52.80%
	q=4						50%

Table S5J. SDP (Specificity diversity permutation) tests for the ES (enriched species) category in the latter class, from pairwise comparison between 10 host animal classes

Treatments	Order	Former	Latter	Delta	Lower (95%)	Upper (95%)	*p*-Value
Chromadorea vs. Malacostraca	*q=0*	62.000	62.000	0.000	6.397	49.449	1.000
	q=1	15.017	45.485	-30.467	-5.980	39.412	0.172
	q=2	10.441	38.916	-28.474	-12.070	33.620	0.045
	q=3	9.092	35.903	-26.811	-14.645	29.821	0.017
	q=4	8.377	34.187	-25.810	-15.124	26.972	0.004
Chromadorea vs. Insecta	*q=0*	2.000	2.000	0.000	0.000	0.000	0.000
	q=1	1.975	1.737	0.239	-0.729	0.577	0.534
	q=2	1.952	1.577	0.375	-0.864	0.711	0.433
	q=3	1.930	1.488	0.442	-0.859	0.724	0.345
	q=4	1.910	1.439	0.471	-0.829	0.707	0.280
Chromadorea vs. Chondrichthyes	*q=0*	NA	NA	NA	NA	NA	NA
	q=1	NA	NA	NA	NA	NA	NA
	q=2	NA	NA	NA	NA	NA	NA
	q=3	NA	NA	NA	NA	NA	NA
	q=4	NA	NA	NA	NA	NA	NA
Chromadorea vs. Actinopteri	*q=0*	NA	NA	NA	NA	NA	NA
	q=1	NA	NA	NA	NA	NA	NA
	q=2	NA	NA	NA	NA	NA	NA
	q=3	NA	NA	NA	NA	NA	NA
	q=4	NA	NA	NA	NA	NA	NA
Chromadorea vs. Amphibia	*q=0*	12.000	12.000	0.000	4.614	13.572	1.000
	q=1	6.533	10.252	-3.718	1.295	13.481	0.887
	q=2	5.389	8.733	-3.343	0.803	12.309	0.882
	q=3	4.976	7.608	-2.633	0.574	11.267	0.899
	q=4	4.771	6.844	-2.074	0.414	10.476	0.916
Chromadorea vs. Sauropsida	*q=0*	27.000	27.000	0.000	0.000	0.000	0.000
	q=1	7.523	22.219	-14.695	-3.071	2.482	0.000
	q=2	5.396	18.762	-13.366	-4.472	3.785	0.000

	q=3	4.772	16.409	-11.637	-5.119	4.453	0.000
	q=4	4.478	14.852	-10.374	-5.291	4.678	0.000
Chromadorea vs. Aves	***q=0***	NA	NA	NA	NA	NA	NA
	q=1	NA	NA	NA	NA	NA	NA
	q=2	NA	NA	NA	NA	NA	NA
	q=3	NA	NA	NA	NA	NA	NA
	q=4	NA	NA	NA	NA	NA	NA
Chromadorea vs. Mammalia	***q=0***	NA	NA	NA	NA	NA	NA
	q=1	NA	NA	NA	NA	NA	NA
	q=2	NA	NA	NA	NA	NA	NA
	q=3	NA	NA	NA	NA	NA	NA
	q=4	NA	NA	NA	NA	NA	NA
Arachnida vs. Insecta	***q=0***	NA	NA	NA	NA	NA	NA
	q=1	NA	NA	NA	NA	NA	NA
	q=2	NA	NA	NA	NA	NA	NA
	q=3	NA	NA	NA	NA	NA	NA
	q=4	NA	NA	NA	NA	NA	NA
Arachnida vs. Chondrichthyes	***q=0***	2.000	2.000	0.000	-0.396	0.462	0.043
	q=1	1.858	1.879	-0.020	-0.700	0.834	0.951
	q=2	1.749	1.782	-0.032	-0.855	1.000	0.946
	q=3	1.672	1.710	-0.038	-0.917	1.062	0.946
	q=4	1.620	1.659	-0.039	-0.943	1.087	0.947
Arachnida vs. Actinopteri	***q=0***	NA	NA	NA	NA	NA	NA
	q=1	NA	NA	NA	NA	NA	NA
	q=2	NA	NA	NA	NA	NA	NA
	q=3	NA	NA	NA	NA	NA	NA
	q=4	NA	NA	NA	NA	NA	NA
Arachnida vs. Amphibia	***q=0***	NA	NA	NA	NA	NA	NA
	q=1	NA	NA	NA	NA	NA	NA
	q=2	NA	NA	NA	NA	NA	NA
	q=3	NA	NA	NA	NA	NA	NA
	q=4	NA	NA	NA	NA	NA	NA
Arachnida vs. Sauropsida	***q=0***	10.000	10.000	0.000	-0.090	0.086	0.002
	q=1	3.884	8.935	-5.051	-1.671	1.086	0.000
	q=2	2.352	8.120	-5.768	-2.221	1.557	0.000
	q=3	1.972	7.549	-5.577	-2.395	1.749	0.000
	q=4	1.834	7.157	-5.324	-2.437	1.822	0.000
Arachnida vs. Aves	***q=0***	NA	NA	NA	NA	NA	NA
	q=1	NA	NA	NA	NA	NA	NA
	q=2	NA	NA	NA	NA	NA	NA
	q=3	NA	NA	NA	NA	NA	NA
	q=4	NA	NA	NA	NA	NA	NA
Arachnida vs. Mammalia	***q=0***	NA	NA	NA	NA	NA	NA
	q=1	NA	NA	NA	NA	NA	NA
	q=2	NA	NA	NA	NA	NA	NA
	q=3	NA	NA	NA	NA	NA	NA
	q=4	NA	NA	0.000	0.000	0.000	0.000
Malacostraca vs. Chondrichthyes	***q=0***	8.000	8.000	0.000	-0.108	0.106	0.003
	q=1	4.761	7.315	-2.555	-1.702	1.667	0.002
	q=2	3.497	6.725	-3.228	-2.132	2.100	0.002
	q=3	2.962	6.244	-3.282	-2.305	2.279	0.003

	q=4	2.702	5.866	-3.164	-2.386	2.363	0.008
Malacostraca vs. Actinopteri	***q=0***	NA	NA	NA	NA	NA	NA
	q=1	NA	NA	NA	NA	NA	NA
	q=2	NA	NA	NA	NA	NA	NA
	q=3	NA	NA	NA	NA	NA	NA
	q=4	NA	NA	NA	NA	NA	NA
Malacostraca vs. Amphibia	***q=0***	1.000	1.000	0.000	-0.436	0.548	0.066
	q=1	1.000	1.000	0.000	-0.436	0.548	0.066
	q=2	1.000	1.000	0.000	-0.436	0.548	0.066
	q=3	1.000	1.000	0.000	-0.436	0.548	0.066
	q=4	1.000	1.000	0.000	-0.436	0.548	0.066
Malacostraca vs. Sauropsida	***q=0***	2.000	2.000	0.000	0.000	0.000	0.000
	q=1	1.165	1.861	-0.695	-0.633	0.541	0.032
	q=2	1.073	1.753	-0.680	-0.834	0.746	0.083
	q=3	1.056	1.677	-0.621	-0.899	0.828	0.164
	q=4	1.049	1.625	-0.575	-0.917	0.859	0.243
Malacostraca vs. Aves	***q=0***	NA	NA	NA	NA	NA	NA
	q=1	NA	NA	NA	NA	NA	NA
	q=2	NA	NA	NA	NA	NA	NA
	q=3	NA	NA	NA	NA	NA	NA
	q=4	NA	NA	NA	NA	NA	NA
Malacostraca vs. Mammalia	***q=0***	NA	NA	NA	NA	NA	NA
	q=1	NA	NA	NA	NA	NA	NA
	q=2	NA	NA	NA	NA	NA	NA
	q=3	NA	NA	NA	NA	NA	NA
	q=4	NA	NA	NA	NA	NA	NA
Insecta vs. Actinopteri	***q=0***	21.000	21.000	0.000	-0.318	0.286	0.021
	q=1	6.103	19.569	-13.466	-3.359	2.814	0.000
	q=2	3.736	18.407	-14.671	-4.813	4.178	0.000
	q=3	3.015	17.444	-14.429	-5.749	5.131	0.000
	q=4	2.711	16.632	-13.920	-6.331	5.758	0.000
Insecta vs. Amphibia	***q=0***	85.000	85.000	0.000	58.028	94.326	1.000
	q=1	10.105	73.715	-63.610	27.890	78.381	0.040
	q=2	5.625	63.434	-57.809	13.612	60.010	0.000
	q=3	4.607	55.567	-50.960	5.821	48.809	0.000
	q=4	4.150	50.055	-45.906	2.123	42.292	0.001
Insecta vs. Sauropsida	***q=0***	281.000	281.000	0.000	7.293	32.195	1.000
	q=1	40.527	162.662	-122.135	-11.260	48.463	0.000
	q=2	13.676	111.703	-98.027	-19.134	45.930	0.000
	q=3	8.350	87.484	-79.135	-21.212	41.289	0.000
	q=4	6.695	74.296	-67.601	-21.156	37.150	0.000
Insecta vs. Aves	***q=0***	2.000	2.000	0.000	-0.643	1.007	0.210
	q=1	1.198	1.449	-0.250	-1.146	1.336	0.780
	q=2	1.092	1.272	-0.180	-1.122	1.250	0.821
	q=3	1.070	1.214	-0.144	-1.064	1.167	0.830
	q=4	1.062	1.189	-0.127	-1.019	1.109	0.832
Insecta vs. Mammalia	***q=0***	21.000	21.000	0.000	-0.110	0.104	0.003
	q=1	8.282	19.901	-11.619	-3.540	2.246	0.000
	q=2	6.140	18.847	-12.707	-5.196	3.702	0.000
	q=3	5.403	17.894	-12.491	-6.162	4.742	0.000
	q=4	5.031	17.073	-12.042	-6.684	5.389	0.000

Chondrichthyes vs. Amphibia	***q=0***	NA	NA	NA	NA	NA	NA
	q=1	NA	NA	NA	NA	NA	NA
	q=2	NA	NA	NA	NA	NA	NA
	q=3	NA	NA	NA	NA	NA	NA
	q=4	NA	NA	NA	NA	NA	NA
Chondrichthyes vs. Sauropsida	***q=0***	NA	NA	NA	NA	NA	NA
	q=1	NA	NA	NA	NA	NA	NA
	q=2	NA	NA	NA	NA	NA	NA
	q=3	NA	NA	NA	NA	NA	NA
	q=4	NA	NA	NA	NA	NA	NA
Chondrichthyes vs. Aves	***q=0***	NA	NA	NA	NA	NA	NA
	q=1	NA	NA	NA	NA	NA	NA
	q=2	NA	NA	NA	NA	NA	NA
	q=3	NA	NA	NA	NA	NA	NA
	q=4	NA	NA	NA	NA	NA	NA
Chondrichthyes vs. Mammalia	***q=0***	NA	NA	NA	NA	NA	NA
	q=1	NA	NA	NA	NA	NA	NA
	q=2	NA	NA	NA	NA	NA	NA
	q=3	NA	NA	NA	NA	NA	NA
	q=4	NA	NA	NA	NA	NA	NA
Actinopteri vs. Sauropsida	***q=0***	85.000	85.000	0.000	3.683	17.375	1.000
	q=1	14.280	42.981	-28.701	-3.666	17.168	0.000
	q=2	8.049	26.641	-18.592	-5.865	13.878	0.002
	q=3	6.463	20.349	-13.887	-6.059	11.763	0.005
	q=4	5.720	17.390	-11.670	-5.823	10.458	0.008
Actinopteri vs. Aves	***q=0***	4.000	4.000	0.000	-0.947	2.897	0.626
	q=1	3.189	3.999	-0.810	-1.201	2.749	0.474
	q=2	3.019	3.998	-0.979	-1.353	2.762	0.412
	q=3	2.940	3.997	-1.057	-1.403	2.738	0.389
	q=4	2.881	3.997	-1.115	-1.415	2.706	0.370
Actinopteri vs. Mammalia	***q=0***	43.000	43.000	0.000	-0.220	0.208	0.009
	q=1	8.859	34.572	-25.713	-8.289	7.409	0.000
	q=2	5.335	26.809	-21.474	-10.921	10.197	0.000
	q=3	4.354	21.344	-16.989	-11.427	10.915	0.000
	q=4	3.896	18.057	-14.161	-11.106	10.745	0.007
Amphibia vs. Aves	***q=0***	NA	NA	NA	NA	NA	NA
	q=1	NA	NA	NA	NA	NA	NA
	q=2	NA	NA	NA	NA	NA	NA
	q=3	NA	NA	NA	NA	NA	NA
	q=4	NA	NA	NA	NA	NA	NA
Amphibia vs. Mammalia	***q=0***	NA	NA	NA	NA	NA	NA
	q=1	NA	NA	NA	NA	NA	NA
	q=2	NA	NA	NA	NA	NA	NA
	q=3	NA	NA	NA	NA	NA	NA
	q=4	NA	NA	NA	NA	NA	NA
Sauropsida vs. Mammalia	***q=0***	1.000	1.000	0.000	0.000	0.000	0.000
	q=1	1.000	1.000	0.000	0.000	0.000	0.000
	q=2	1.000	1.000	0.000	0.000	0.000	0.000
	q=3	1.000	1.000	0.000	0.000	0.000	0.000
	q=4	1.000	1.000	0.000	0.000	0.000	0.000
Percentage of	***q=0***						77.80%

Significant Differences (%)	***q=1***	80.60%
	q=2	80.60%
	q=3	80.60%
	q=4	80.60%

Table S5K. SDP (Specificity diversity permutation) tests for all species with significant differences in specificity, from pairwise comparison between 10 host animal classes

Treatments	Order	Former	Latter	Delta	Lower (95%)	Upper (95%)	*p*-Value
Chromadorea vs. Malacostraca	***q=0***	79.000	3618.000	-3539.000	1646.277	3711.051	0.026
	q=1	18.234	2796.433	-2778.199	694.067	2571.333	0.000
	q=2	16.527	1856.764	-1840.237	-354.488	1244.311	0.000
	q=3	15.814	1275.608	-1259.794	-516.002	688.791	0.000
	q=4	15.297	1001.793	-986.496	-444.301	494.644	0.000
Chromadorea vs. Insecta	***q=0***	7068.000	311.000	6757.000	-5276.800	-3059.342	0.000
	q=1	3382.146	103.720	3278.426	-2167.079	-455.567	0.000
	q=2	1344.856	93.151	1251.706	-757.214	337.491	0.000
	q=3	774.402	85.917	688.485	-455.033	282.625	0.000
	q=4	559.746	80.705	479.041	-348.628	217.543	0.000
Chromadorea vs. Chondrichthyes	***q=0***	20.000	2711.000	-2691.000	943.005	3030.691	0.007
	q=1	19.190	2214.602	-2195.412	85.427	2246.485	0.000
	q=2	18.417	1458.452	-1440.035	-533.384	1076.880	0.000
	q=3	17.720	837.263	-819.543	-530.782	600.988	0.010
	q=4	17.120	544.972	-527.851	-430.462	436.748	0.026
Chromadorea vs. Actinopteri	***q=0***	7309.000	446.000	6863.000	-5929.303	-3770.103	0.000
	q=1	3517.553	268.637	3248.915	-1328.396	47.147	0.000
	q=2	1390.695	220.180	1170.516	-424.667	284.310	0.000
	q=3	798.232	187.236	610.996	-270.429	203.270	0.001
	q=4	577.088	166.157	410.930	-211.131	160.720	0.001
Chromadorea vs. Amphibia	***q=0***	20.000	2812.000	-2792.000	1380.217	3159.369	0.158
	q=1	7.933	2202.312	-2194.379	712.496	2303.601	0.000
	q=2	7.836	1603.419	-1595.583	-356.458	1345.882	0.000
	q=3	7.758	1233.927	-1226.169	-563.532	764.654	0.000
	q=4	7.686	1042.496	-1034.810	-523.802	553.493	0.001
Chromadorea vs. Sauropsida	***q=0***	7305.000	1602.000	5703.000	-2584.444	253.754	0.000
	q=1	3515.870	1179.731	2336.139	-1275.898	712.463	0.000
	q=2	1372.375	870.778	501.598	-647.267	514.604	0.105
	q=3	784.156	659.323	124.832	-434.512	364.243	0.573
	q=4	566.804	528.663	38.142	-335.888	287.387	0.829
Chromadorea vs. Aves	***q=0***	7442.000	719.000	6723.000	-4096.990	-1327.682	0.000
	q=1	3592.497	605.114	2987.383	-1801.026	327.936	0.000
	q=2	1419.184	516.105	903.079	-822.073	474.578	0.012
	q=3	813.011	446.110	366.901	-540.839	348.882	0.138
	q=4	587.155	394.848	192.307	-427.677	278.882	0.348
Chromadorea vs. Mammalia	***q=0***	7335.000	81.000	7254.000	-6181.845	-4160.991	0.000
	q=1	3534.635	15.399	3519.236	-3268.627	-1687.302	0.000
	q=2	1397.806	14.830	1382.975	-1262.908	25.098	0.003
	q=3	801.693	14.584	787.108	-712.018	326.483	0.002
	q=4	579.229	14.397	564.832	-526.888	309.003	0.009
Arachnida vs. Insecta	***q=0***	3586.000	17.000	3569.000	-4710.838	-1272.522	0.066

	q=1	2537.323	9.098	2528.225	-2750.674	-2172.586	0.454
	q=2	1201.343	6.455	1194.888	-1702.122	-1073.827	0.948
	q=3	608.145	5.178	602.967	-1021.188	-452.426	0.900
	q=4	413.108	4.536	408.572	-678.339	-257.873	0.770
Arachnida vs. Chondrichthyes	***q=0***	84.000	2711.000	-2627.000	-1023.294	1918.688	0.000
	q=1	68.623	2215.102	-2146.479	-1138.843	1756.798	0.000
	q=2	56.712	1459.297	-1402.584	-1174.363	1481.921	0.039
	q=3	48.170	837.638	-789.468	-1030.641	1140.728	0.172
	q=4	42.595	545.031	-502.436	-832.371	873.401	0.251
Arachnida vs. Actinopteri	***q=0***	3608.000	50.000	3558.000	-3859.344	-2789.586	0.235
	q=1	2556.295	25.705	2530.590	-962.659	-73.109	0.000
	q=2	1210.143	25.346	1184.797	-248.560	233.242	0.000
	q=3	611.644	25.037	586.606	-170.776	190.702	0.000
	q=4	415.179	24.749	390.430	-138.819	155.665	0.000
Arachnida vs. Amphibia	***q=0***	40.000	2842.000	-2802.000	-206.724	2124.832	0.001
	q=1	35.539	2229.986	-2194.447	-398.530	1817.131	0.001
	q=2	31.648	1625.477	-1593.829	-731.228	1510.330	0.005
	q=3	28.643	1250.966	-1222.323	-934.680	1241.880	0.021
	q=4	26.440	1056.440	-1030.000	-923.516	1039.659	0.024
Arachnida vs. Sauropsida	***q=0***	3589.000	145.000	3444.000	-3460.514	-1691.182	0.005
	q=1	2531.915	133.569	2398.346	-1098.653	168.830	0.000
	q=2	1197.873	122.485	1075.387	-369.860	319.308	0.000
	q=3	606.500	111.673	494.828	-229.358	224.139	0.001
	q=4	412.097	102.118	309.978	-175.251	170.447	0.004
Arachnida vs. Aves	***q=0***	3639.000	28.000	3611.000	-3743.919	-2251.321	0.027
	q=1	2582.345	26.642	2555.703	-1929.906	-629.343	0.000
	q=2	1223.789	26.260	1197.529	-613.572	291.242	0.000
	q=3	617.799	25.867	591.932	-349.259	297.384	0.000
	q=4	418.963	25.468	393.495	-263.672	240.867	0.009
Arachnida vs. Mammalia	***q=0***	3612.000	6.000	3606.000	-5303.192	85.992	0.050
	q=1	2559.417	2.602	2556.814	-2767.095	-2306.190	0.633
	q=2	1211.343	2.281	1209.062	-1757.734	-1040.018	0.952
	q=3	612.158	2.199	609.959	-1050.975	-437.166	0.941
	q=4	415.512	2.162	413.349	-697.646	-265.794	0.844
Malacostraca vs. Chondrichthyes	***q=0***	3709.000	52.000	3657.000	-1423.423	1209.803	0.000
	q=1	2835.041	46.886	2788.154	-1298.869	1136.952	0.000
	q=2	1859.627	41.394	1818.233	-1180.630	1084.838	0.000
	q=3	1274.819	36.111	1238.708	-1058.784	1016.119	0.012
	q=4	1003.298	31.663	971.635	-931.600	911.437	0.037
Malacostraca vs. Actinopteri	***q=0***	3707.000	42136.000	-38429.000	-44749.236	-42212.590	1.000
	q=1	2843.739	12781.672	-9937.933	-27715.207	-17867.002	1.000
	q=2	1869.875	889.258	980.617	-3427.524	44.686	0.782
	q=3	1281.759	454.688	827.071	-1214.739	800.452	0.140
	q=4	1007.514	352.908	654.606	-886.259	863.935	0.130
Malacostraca vs. Amphibia	***q=0***	113.000	2830.000	-2717.000	-688.812	1654.750	0.000
	q=1	107.359	2217.775	-2110.416	-759.029	1492.336	0.000
	q=2	103.626	1616.256	-1512.630	-861.928	1347.491	0.005
	q=3	100.598	1244.334	-1143.736	-923.928	1222.893	0.035
	q=4	98.099	1051.254	-953.155	-915.196	1107.800	0.058
Malacostraca vs. Sauropsida	***q=0***	3673.000	102.000	3571.000	-3906.930	-1992.854	0.085
	q=1	2823.339	57.319	2766.020	-1576.344	44.668	0.000

	q=2	1859.694	54.023	1805.672	-523.678	428.569	0.000
	q=3	1274.645	51.013	1223.631	-321.633	323.988	0.000
	q=4	1001.528	48.305	953.223	-242.330	245.088	0.000
Malacostraca vs. Aves	***q=0***	3719.000	24.000	3695.000	-4033.381	-2453.975	0.162
	q=1	2850.225	4.060	2846.165	-2909.127	-1476.898	0.000
	q=2	1871.206	3.991	1867.214	-1415.817	100.132	0.000
	q=3	1282.547	3.973	1278.573	-680.962	447.232	0.000
	q=4	1008.942	3.959	1004.983	-475.464	451.608	0.001
Malacostraca vs. Mammalia	***q=0***	3689.000	179058.000	-175369.000	-178621.900	-172329.110	0.558
	q=1	2830.217	173044.621	-170214.405	-170797.651	-164110.761	0.026
	q=2	1862.797	163320.315	-161457.519	-156555.317	-147377.151	0.000
	q=3	1277.955	133130.164	-131852.208	-119949.663	-98336.745	0.000
	q=4	1005.170	68876.537	-67871.367	-64301.776	-47298.775	0.018
Insecta vs. Actinopteri	***q=0***	35568.000	3366.000	32202.000	-9645.648	774.240	0.000
	q=1	16912.859	1730.640	15182.219	-5123.562	2503.847	0.000
	q=2	4702.366	884.876	3817.490	-2063.200	1777.814	0.000
	q=3	2176.361	583.572	1592.789	-1114.764	1023.827	0.003
	q=4	1479.645	459.931	1019.714	-768.076	711.123	0.005
Insecta vs. Amphibia	***q=0***	31389.000	2837.000	28552.000	31223.864	34161.984	0.999
	q=1	30299.337	2225.250	28074.087	29093.465	32080.795	0.993
	q=2	19742.628	1621.430	18121.198	18685.544	27785.387	0.982
	q=3	3651.223	1247.773	2403.450	1838.021	15680.282	0.997
	q=4	1478.023	1053.831	424.192	-364.795	7097.468	0.974
Insecta vs. Sauropsida	***q=0***	610.000	25364.000	-24754.000	9378.394	14858.058	0.000
	q=1	276.563	8281.176	-8004.614	2503.338	5375.159	0.000
	q=2	221.069	2721.760	-2500.691	-256.575	1441.746	0.000
	q=3	185.345	1589.703	-1404.358	-356.128	787.540	0.000
	q=4	163.384	1156.698	-993.313	-294.496	585.919	0.000
Insecta vs. Aves	***q=0***	1004.000	29188.000	-28184.000	5467.019	11735.703	0.000
	q=1	738.884	11728.557	-10989.673	768.447	5808.279	0.000
	q=2	511.523	3868.255	-3356.732	-938.078	2076.121	0.000
	q=3	379.984	2229.655	-1849.671	-774.021	1154.366	0.000
	q=4	312.829	1663.659	-1350.830	-615.267	877.487	0.000
Insecta vs. Mammalia	***q=0***	35910.000	3412.000	32498.000	-12253.718	-1804.420	0.000
	q=1	17162.444	2900.349	14262.094	-7023.560	1622.202	0.000
	q=2	4793.234	2403.212	2390.022	-3373.477	2097.500	0.117
	q=3	2215.995	1962.503	253.492	-2068.673	1515.149	0.781
	q=4	1505.146	1637.145	-131.999	-1549.343	1163.166	0.839
Chondrichthyes vs. Amphibia	***q=0***	30.000	2844.000	-2814.000	-558.890	1720.762	0.000
	q=1	27.515	2231.706	-2204.192	-583.997	1512.776	0.000
	q=2	24.948	1626.706	-1601.758	-685.809	1323.039	0.001
	q=3	22.435	1251.816	-1229.382	-829.936	1186.538	0.013
	q=4	20.216	1057.095	-1036.878	-884.369	1076.713	0.023
Chondrichthyes vs. Sauropsida	***q=0***	2708.000	59.000	2649.000	-3085.515	-1294.141	0.100
	q=1	2211.914	56.232	2155.682	-1183.678	208.020	0.000
	q=2	1456.596	53.350	1403.247	-401.238	338.810	0.000
	q=3	836.291	50.527	785.764	-245.312	233.789	0.000
	q=4	544.401	47.936	496.465	-183.498	172.044	0.000
Chondrichthyes vs. Aves	***q=0***	2659.000	33.000	2626.000	-3030.165	-1618.327	0.133
	q=1	2167.971	1.099	2166.872	-2541.875	-1054.419	0.004
	q=2	1425.313	1.023	1424.290	-1570.246	-71.260	0.008

	q=3	819.181	1.018	818.163	-787.630	370.814	0.005
	q=4	534.112	1.016	533.097	-528.335	414.897	0.030
Chondrichthyes vs. Mammalia	***q=0***	2515.000	174427.000	-171912.000	-171964.678	-165409.034	0.011
	q=1	2025.278	169295.436	-167270.157	-165263.538	-158489.963	0.000
	q=2	1308.150	161020.856	-159712.705	-151778.365	-143219.857	0.000
	q=3	748.588	130143.245	-129394.656	-115870.350	-95375.592	0.000
	q=4	490.961	64795.065	-64304.104	-62647.180	-44772.378	0.033
Actinopteri vs. Sauropsida	***q=0***	813.000	25608.000	-24795.000	11696.978	16956.122	0.000
	q=1	535.937	8378.917	-7842.981	1217.080	4748.061	0.000
	q=2	386.256	2751.843	-2365.586	-908.494	1463.778	0.000
	q=3	301.071	1603.943	-1302.872	-752.716	919.852	0.000
	q=4	254.909	1163.509	-908.600	-598.185	695.945	0.001
Actinopteri vs. Aves	***q=0***	1352.000	29256.000	-27904.000	8632.004	14855.748	0.000
	q=1	885.466	11768.289	-10882.823	429.344	5235.286	0.000
	q=2	564.319	3879.223	-3314.904	-1115.052	1677.992	0.000
	q=3	409.815	2234.693	-1824.878	-812.948	974.659	0.000
	q=4	335.772	1667.021	-1331.249	-606.599	698.800	0.000
Actinopteri vs. Mammalia	***q=0***	49225.000	7894.000	41331.000	-7964.056	449.812	0.000
	q=1	13492.157	6458.032	7034.125	-4504.746	1994.393	0.000
	q=2	2707.904	4948.140	-2240.236	-3122.069	2779.447	0.146
	q=3	1365.627	3719.745	-2354.118	-2017.118	1933.473	0.014
	q=4	979.384	2925.385	-1946.002	-1365.138	1317.131	0.008
Amphibia vs. Aves	***q=0***	2844.000	5.000	2839.000	-3932.977	720.345	0.062
	q=1	2231.700	1.716	2229.985	-2487.372	-1614.920	0.373
	q=2	1626.697	1.440	1625.258	-1862.050	-1202.359	0.524
	q=3	1251.809	1.351	1250.458	-1474.299	-880.875	0.584
	q=4	1057.089	1.311	1055.778	-1236.917	-696.719	0.476
Amphibia vs. Mammalia	***q=0***	535.000	46.000	489.000	-798.307	-79.289	0.764
	q=1	475.812	6.831	468.981	-626.695	-186.248	0.000
	q=2	428.628	4.353	424.274	-538.160	-89.367	0.000
	q=3	394.573	3.667	390.906	-445.339	-10.524	0.000
	q=4	370.878	3.356	367.522	-369.551	35.851	0.000
Sauropsida vs. Mammalia	***q=0***	25253.000	307.000	24946.000	-17831.626	-12921.130	0.000
	q=1	8211.856	135.027	8076.830	-6485.472	-4011.500	0.000
	q=2	2706.106	128.403	2577.703	-1688.054	16.338	0.000
	q=3	1582.554	123.572	1458.982	-870.798	324.644	0.000
	q=4	1150.198	119.150	1031.048	-638.619	295.501	0.000
Percentage of Significant Differences (%)	***q=0***						63.90%
	q=1						86.10%
	q=2						77.80%
	q=3						75%
	q=4						72.20%

Table S5L. SDP (Specificity diversity permutation) tests for all species without considering specificity, from pairwise comparison between 10 host animal classes

Treatments	Order	Former	Latter	Delta	Lower (95%)	Upper (95%)	*p*-Value
Chromadorea vs. Malacostraca	***q=0***	7470.000	3744.000	3726.000	6750.210	9129.196	1.000
	q=1	3598.813	2860.393	738.420	3500.020	5836.296	1.000
	q=2	1410.219	1898.445	-488.225	604.107	2688.216	0.982
	q=3	800.625	1303.248	-502.623	-341.095	1429.403	0.554
	q=4	576.021	1022.298	-446.277	-507.723	1027.052	0.379
Chromadorea vs. Insecta	***q=0***	7470.000	36312.000	-28842.000	-30047.900	-22111.590	0.085
	q=1	3586.271	17330.120	-13743.849	-18182.399	-10919.935	0.681
	q=2	1415.871	4794.482	-3378.611	-6538.279	-558.280	0.536
	q=3	808.195	2194.748	-1386.554	-2964.341	1396.721	0.339
	q=4	581.665	1486.401	-904.736	-1970.139	1182.636	0.361
Chromadorea vs. Chondrichthyes	***q=0***	7470.000	2711.000	4759.000	5929.731	8472.021	0.999
	q=1	3609.325	2214.602	1394.723	2922.907	5586.690	1.000
	q=2	1426.300	1458.452	-32.151	343.420	2645.910	0.998
	q=3	816.795	837.263	-20.468	-455.908	1411.140	0.980
	q=4	589.699	544.972	44.728	-564.971	1005.762	0.939
Chromadorea vs. Actinopteri	***q=0***	7470.000	50097.000	-42627.000	-41564.108	-35119.864	0.002
	q=1	3596.589	13763.011	-10166.422	-17930.493	-11226.528	0.995
	q=2	1417.288	2740.974	-1323.686	-4205.528	1391.168	0.565
	q=3	810.607	1378.127	-567.519	-1926.114	1509.532	0.547
	q=4	585.069	987.343	-402.274	-1275.305	1076.234	0.519
Chromadorea vs. Amphibia	***q=0***	7470.000	2844.000	4626.000	7041.171	9175.147	1.000
	q=1	3619.786	2224.001	1395.785	3647.360	5832.787	1.000
	q=2	1423.196	1619.059	-195.863	711.951	2812.556	0.997
	q=3	811.452	1245.376	-433.923	-250.859	1550.662	0.704
	q=4	585.484	1051.583	-466.099	-469.062	1112.773	0.436
Chromadorea vs. Sauropsida	***q=0***	7470.000	25720.000	-18250.000	-7929.922	-2904.616	0.000
	q=1	3592.255	8429.075	-4836.820	-3991.796	-389.010	0.003
	q=2	1412.443	2761.750	-1349.307	-1751.893	1120.946	0.088
	q=3	807.186	1610.966	-803.780	-1004.604	845.244	0.089
	q=4	582.525	1170.978	-588.453	-716.596	623.283	0.083
Chromadorea vs. Aves	***q=0***	7470.000	29298.000	-21828.000	-16157.798	-11036.388	0.000
	q=1	3606.557	11777.363	-8170.806	-9572.652	-3873.466	0.166
	q=2	1424.350	3881.904	-2457.554	-3974.870	946.306	0.233
	q=3	815.457	2235.963	-1420.506	-2015.965	1154.184	0.130
	q=4	588.735	1667.868	-1079.133	-1385.307	876.568	0.093
Chromadorea vs. Mammalia	***q=0***	7470.000	207896.000	-200426.000	-163666.501	-145707.295	0.000
	q=1	3597.458	130008.866	-126411.408	-113087.677	-93474.597	0.000
	q=2	1419.636	56187.828	-54768.192	-57031.484	-33307.600	0.057
	q=3	812.035	27254.301	-26442.266	-26230.357	-4012.606	0.026
	q=4	585.916	17341.796	-16755.879	-15450.468	3246.500	0.011
Arachnida vs. Insecta	***q=0***	3645.000	36312.000	-32667.000	-37971.920	-33291.826	0.982
	q=1	2577.992	17389.237	-14811.244	-22554.589	-17895.229	1.000
	q=2	1219.169	4887.133	-3667.964	-8576.031	-3815.884	0.974
	q=3	615.596	2255.190	-1639.594	-4018.716	20.315	0.641
	q=4	417.618	1528.393	-1110.775	-2668.500	802.622	0.470
Arachnida vs. Chondrichthyes	***q=0***	3645.000	2711.000	934.000	-425.306	2533.804	0.581
	q=1	2585.954	2215.102	370.852	-576.119	2254.728	0.789

	q=2	1225.516	1459.297	-233.780	-826.203	1864.541	0.806
	q=3	618.538	837.638	-219.100	-1044.832	1474.941	0.742
	q=4	419.410	545.031	-125.621	-980.038	1144.860	0.820
Arachnida vs. Actinopteri	***q=0***	3645.000	50097.000	-46452.000	-50542.057	-46898.759	0.984
	q=1	2582.384	13838.126	-11255.742	-21109.115	-15722.504	1.000
	q=2	1222.030	2761.785	-1539.755	-5233.421	-643.338	0.893
	q=3	616.674	1386.924	-770.250	-2465.229	1111.326	0.519
	q=4	418.227	992.982	-574.756	-1741.248	1210.727	0.495
Arachnida vs. Amphibia	***q=0***	3645.000	2844.000	801.000	1128.965	3318.083	0.996
	q=1	2586.012	2231.652	354.360	826.196	2764.292	0.998
	q=2	1225.539	1626.635	-401.096	217.480	2108.512	0.943
	q=3	618.547	1251.758	-633.211	-565.372	1653.047	0.453
	q=4	419.416	1057.047	-637.632	-926.520	1391.724	0.324
Arachnida vs. Sauropsida	***q=0***	3645.000	25720.000	-22075.000	-21999.726	-18980.064	0.015
	q=1	2565.072	8431.559	-5866.487	-9961.067	-7233.092	1.000
	q=2	1212.822	2772.689	-1559.867	-2841.839	-464.932	0.576
	q=3	612.833	1615.493	-1002.659	-1333.999	491.217	0.109
	q=4	415.937	1170.397	-754.460	-912.369	508.370	0.063
Arachnida vs. Aves	***q=0***	3645.000	29298.000	-25653.000	-27908.881	-24798.891	0.815
	q=1	2585.954	11790.673	-9204.719	-15465.112	-11120.753	1.000
	q=2	1225.334	3885.544	-2660.210	-5577.700	-1827.131	0.852
	q=3	618.440	2237.628	-1619.188	-2628.498	217.398	0.284
	q=4	419.350	1668.975	-1249.625	-1792.079	555.766	0.142
Arachnida vs. Mammalia	***q=0***	3645.000	207896.000	-204251.000	-201351.393	-192077.965	0.000
	q=1	2580.215	130035.245	-127455.029	-136291.539	-122681.781	0.733
	q=2	1221.432	56193.857	-54972.424	-69019.169	-49753.729	0.817
	q=3	616.625	27269.238	-26652.613	-34040.168	-18326.106	0.452
	q=4	418.237	17357.045	-16938.808	-20090.405	-7838.139	0.166
Malacostraca vs. Chondrichthyes	***q=0***	3744.000	2711.000	1033.000	-1784.968	1426.368	0.218
	q=1	2856.543	2216.083	640.460	-1693.275	1410.344	0.426
	q=2	1873.279	1468.393	404.886	-1637.479	1448.498	0.635
	q=3	1283.602	846.465	437.137	-1547.751	1452.470	0.581
	q=4	1009.719	551.658	458.060	-1382.144	1339.961	0.524
Malacostraca vs. Actinopteri	***q=0***	3744.000	50097.000	-46353.000	-51758.035	-48573.825	1.000
	q=1	2863.857	13802.179	-10938.322	-21396.027	-15894.961	1.000
	q=2	1881.059	2750.344	-869.285	-5391.971	-1164.733	0.987
	q=3	1288.288	1381.900	-93.612	-2519.908	711.945	0.934
	q=4	1012.130	989.745	22.385	-1770.789	1003.111	0.980
Malacostraca vs. Amphibia	***q=0***	3744.000	2844.000	900.000	-133.163	2337.873	0.617
	q=1	2865.090	2229.031	636.059	-291.068	2084.470	0.656
	q=2	1882.122	1624.293	257.828	-628.821	1923.306	0.806
	q=3	1289.789	1250.010	39.779	-979.619	1817.730	0.960
	q=4	1014.084	1055.672	-41.588	-1132.702	1676.385	0.963
Malacostraca vs. Sauropsida	***q=0***	3744.000	25720.000	-21976.000	-24307.493	-21486.341	0.887
	q=1	2866.834	8404.591	-5537.756	-11083.857	-8149.122	1.000
	q=2	1889.204	2752.671	-863.467	-3320.989	-846.276	0.971
	q=3	1294.756	1601.823	-307.067	-1603.327	404.088	0.781
	q=4	1016.714	1161.811	-145.097	-1113.321	539.151	0.796
Malacostraca vs. Aves	***q=0***	3744.000	29298.000	-25554.000	-29669.537	-26928.405	1.000
	q=1	2869.340	11773.016	-8903.676	-16393.262	-12054.980	1.000

	q=2	1884.929	3880.403	-1995.474	-6052.952	-2418.750	0.994
	q=3	1291.473	2235.275	-943.802	-2893.460	-183.562	0.806
	q=4	1015.417	1667.411	-651.994	-1957.101	347.752	0.625
Malacostraca vs. Mammalia	***q=0***	3744.000	207896.000	-204152.000	-205094.275	-197506.995	0.048
	q=1	2858.328	130040.650	-127182.322	-137647.781	-125204.716	0.906
	q=2	1877.941	56166.702	-54288.761	-69070.612	-51219.847	0.895
	q=3	1286.666	27238.376	-25951.710	-34506.524	-20371.890	0.646
	q=4	1011.311	17340.230	-16328.919	-20674.514	-10053.064	0.345
Insecta vs. Actinopteri	***q=0***	36312.000	50097.000	-13785.000	-16563.996	-4013.650	0.145
	q=1	17311.053	13713.515	3597.538	-10090.149	1459.944	0.605
	q=2	4814.783	2736.389	2078.395	-4437.721	3685.364	0.329
	q=3	2218.852	1376.437	842.415	-2149.919	2010.302	0.423
	q=4	1505.390	986.261	519.129	-1384.535	1305.556	0.447
Insecta vs. Amphibia	***q=0***	36312.000	2844.000	33468.000	35283.167	38653.605	0.999
	q=1	17401.032	2231.020	15170.011	18280.737	22300.666	0.999
	q=2	4872.402	1625.407	3246.994	4152.745	8476.068	0.993
	q=3	2244.547	1250.482	994.065	648.347	4019.013	0.935
	q=4	1521.264	1055.908	465.356	-219.831	2626.917	0.861
Insecta vs. Sauropsida	***q=0***	36312.000	25720.000	10592.000	25193.051	34291.361	1.000
	q=1	17312.994	8396.720	8916.273	9991.164	18135.834	0.992
	q=2	4810.146	2750.174	2059.972	-755.056	5662.005	0.596
	q=3	2210.105	1603.000	607.105	-1418.807	2517.784	0.594
	q=4	1497.154	1165.356	331.798	-1036.616	1643.229	0.658
Insecta vs. Aves	***q=0***	36312.000	29298.000	7014.000	13666.483	25149.839	1.000
	q=1	17399.508	11770.233	5629.275	4705.400	15511.728	0.938
	q=2	4892.152	3879.021	1013.131	-2005.343	6010.639	0.756
	q=3	2257.358	2234.568	22.789	-1968.352	2824.588	0.982
	q=4	1529.922	1666.924	-137.002	-1410.415	1903.413	0.902
Insecta vs. Mammalia	***q=0***	36312.000	207896.000	-171584.000	-61506.909	-33887.635	0.000
	q=1	17347.691	130028.035	-112680.344	-45181.478	-17521.838	0.000
	q=2	4855.257	56280.217	-51424.959	-26506.755	3815.006	0.000
	q=3	2240.150	27377.736	-25137.586	-14404.124	9216.090	0.000
	q=4	1519.797	17458.342	-15938.545	-9206.574	6937.203	0.000
Chondrichthyes vs. Amphibia	***q=0***	2711.000	2844.000	-133.000	-324.189	2585.959	0.956
	q=1	2214.602	2231.706	-17.104	-476.179	2326.889	0.987
	q=2	1458.452	1626.706	-168.254	-697.545	2067.017	0.877
	q=3	837.263	1251.816	-414.553	-958.534	1823.035	0.618
	q=4	544.972	1057.095	-512.123	-1120.391	1622.757	0.485
Chondrichthyes vs. Sauropsida	***q=0***	2711.000	25720.000	-23009.000	-23313.470	-20364.292	0.048
	q=1	2214.329	8415.287	-6200.958	-10601.764	-7704.209	0.999
	q=2	1458.139	2762.510	-1304.371	-3140.723	-787.171	0.863
	q=3	837.077	1609.168	-772.091	-1495.393	356.711	0.368
	q=4	544.861	1167.153	-622.292	-1023.657	466.556	0.206
Chondrichthyes vs. Aves	***q=0***	2711.000	29298.000	-26587.000	-28565.247	-25729.097	0.801
	q=1	2209.073	11759.981	-9550.908	-15690.281	-11420.967	1.000
	q=2	1452.141	3877.147	-2425.007	-5736.499	-2227.545	0.954
	q=3	833.051	2233.836	-1400.784	-2743.599	-129.803	0.525
	q=4	542.267	1666.457	-1124.190	-1859.883	341.884	0.277
Chondrichthyes vs. Mammalia	***q=0***	2711.000	207896.000	-205185.000	-202921.361	-194593.973	0.000

	q=1	2152.761	129740.199	-127587.438	-135723.252	-123152.511	0.721
	q=2	1380.661	55948.731	-54568.070	-67584.609	-50302.884	0.832
	q=3	784.059	27086.970	-26302.911	-33446.453	-19813.166	0.535
	q=4	511.764	17229.237	-16717.473	-19957.934	-9542.473	0.219
Actinopteri vs. Sauropsida	***q=0***	50097.000	25720.000	24377.000	38784.945	46833.885	1.000
	q=1	13807.113	8414.754	5392.359	12168.167	19438.560	1.000
	q=2	2750.287	2760.695	-10.408	-1620.192	4631.003	0.996
	q=3	1381.863	1608.031	-226.169	-1807.824	2288.424	0.862
	q=4	989.726	1166.151	-176.425	-1342.421	1568.455	0.841
Actinopteri vs. Aves	***q=0***	50097.000	29298.000	20799.000	27172.356	36205.342	1.000
	q=1	13850.008	11782.344	2067.664	7907.724	17065.823	1.000
	q=2	2762.981	3883.059	-1120.078	-2490.208	4730.790	0.627
	q=3	1387.295	2236.472	-849.177	-1922.481	2255.242	0.441
	q=4	993.213	1668.205	-674.992	-1320.282	1476.658	0.352
Actinopteri vs. Mammalia	***q=0***	50097.000	207896.000	-157799.000	-33489.006	-6026.772	0.000
	q=1	13655.202	129946.600	-116291.398	-25870.124	3019.340	0.000
	q=2	2728.774	56122.261	-53393.488	-18487.254	14687.059	0.000
	q=3	1373.719	27226.363	-25852.644	-9498.045	9123.269	0.000
	q=4	984.543	17328.151	-16343.608	-5444.233	5282.706	0.000
Amphibia vs. Aves	***q=0***	2844.000	29298.000	-26454.000	-29938.316	-27565.022	1.000
	q=1	2231.700	11790.628	-9558.927	-16167.266	-12154.944	1.000
	q=2	1626.697	3885.796	-2259.099	-5953.299	-2742.639	0.992
	q=3	1251.809	2237.759	-985.949	-2883.806	-564.995	0.892
	q=4	1057.089	1669.062	-611.973	-1943.803	47.765	0.760
Amphibia vs. Mammalia	***q=0***	2844.000	207896.000	-205052.000	-206910.503	-200010.635	0.187
	q=1	2231.333	130126.541	-127895.208	-137700.898	-125773.864	0.891
	q=2	1624.121	56323.919	-54699.798	-68195.040	-51011.670	0.854
	q=3	1246.923	27352.874	-26105.951	-34202.383	-20904.538	0.642
	q=4	1051.266	17415.809	-16364.543	-20799.750	-10972.806	0.413
Sauropsida vs. Mammalia	***q=0***	25720.000	207896.000	-182176.000	-155242.035	-135788.263	0.000
	q=1	8356.318	130123.229	-121766.911	-101672.726	-79811.944	0.000
	q=2	2740.330	56243.575	-53503.245	-43769.103	-17286.858	0.000
	q=3	1598.306	27236.248	-25637.942	-18836.780	4990.820	0.000
	q=4	1160.397	17310.263	-16149.866	-11792.118	7118.810	0.000
Percentage of Significant Differences (%)	***q=0***						33.30%
	q=1						13.90%
	q=2						8.30%
	q=3						11.10%
	q=4						11.10%

SDP (Specificity diversity permutation) tests for the pairwise comparisons of the three diet types (Table S6A-S6F)

Table S6A. SDP (specificity diversity permutation) tests for the species category of the US (unique species) in the former diet type

Treatments	Order	Former	Latter	Delta	Lower (95%)	Upper (95%)	*p*-Value
Carnivore *vs.* Herbivore	***q=0***	64500.000	NA	64500.000	-6166.198	2134.962	0.000
	q=1	21539.024	NA	21539.024	-5418.328	3050.587	0.000
	q=2	3461.290	NA	3461.290	-4820.145	4507.810	0.152
	q=3	1552.630	NA	1552.630	-2581.731	2623.241	0.242
	q=4	1083.076	NA	1083.076	-1574.728	1614.033	0.186
Carnivore *vs.* Omnivore	***q=0***	63821.000	NA	63821.000	-4543.804	3885.566	0.000
	q=1	21283.546	NA	21283.546	-4536.554	4152.565	0.000
	q=2	3144.847	NA	3144.847	-4645.188	4674.810	0.188
	q=3	1420.738	NA	1420.738	-2433.875	2504.547	0.250
	q=4	999.805	NA	999.805	-1466.462	1514.350	0.188
Herbivore *vs.* Omnivore	***q=0***	8836.000	NA	8836.000	-737.964	1069.934	0.000
	q=1	6247.670	NA	6247.670	-1286.312	1517.791	0.000
	q=2	3568.761	NA	3568.761	-1472.120	1642.943	0.000
	q=3	2160.887	NA	2160.887	-1331.096	1443.912	0.002
	q=4	1586.444	NA	1586.444	-1104.792	1180.661	0.006
Percentage of Significant Differences (%)	***q=0***						100%
	q=1						100%
	q=2						33.30%
	q=3						33.30%
	q=4						33.30%

Table 6B. SDP (specificity diversity permutation) tests for the species category of the US (unique species) in the latter diet type

Treatments	Order	Former	Latter	Delta	Lower (95%)	Upper (95%)	*p*-Value
Carnivore *vs.* Herbivore	***q=0***	NA	8122.000	-8122.000	-907.674	572.802	0.000
	q=1	NA	5828.550	-5828.550	-1364.313	1139.466	0.000
	q=2	NA	3469.194	-3469.194	-1459.059	1300.200	0.000
	q=3	NA	2154.282	-2154.282	-1292.005	1187.974	0.000
	q=4	NA	1590.612	-1590.612	-1073.317	1001.354	0.001
Carnivore *vs.* Omnivore	***q=0***	NA	7300.000	-7300.000	-375.937	308.357	0.000
	q=1	NA	5221.560	-5221.560	-523.560	476.036	0.000
	q=2	NA	3509.759	-3509.759	-549.283	515.437	0.000
	q=3	NA	2507.760	-2507.760	-529.224	505.823	0.000
	q=4	NA	1971.577	-1971.577	-499.430	482.857	0.000
Herbivore *vs.* Omnivore	***q=0***	NA	105874.000	-105874.000	-4073.777	9551.791	0.000
	q=1	NA	57027.453	-57027.453	-4154.599	8136.776	0.000
	q=2	NA	20151.141	-20151.141	-5821.634	7834.373	0.000
	q=3	NA	9922.728	-9922.728	-6585.838	7194.434	0.002
	q=4	NA	6677.199	-6677.199	-5384.085	5605.267	0.022
Percentage of Significant Differences (%)	***q=0***						100%
	q=1						100%
	q=2						100%
	q=3						100%
	q=4						100%

Table S6C. SDP (specificity diversity permutation) tests for the species category of the ES (enriched species) in the former diet type

Treatments	Order	Former	Latter	Delta	Lower (95%)	Upper (95%)	*p*-Value
Carnivore *vs.* Herbivore	***q=0***	512.000	512.000	0.000	-9.714	7.632	0.895
	q=1	432.836	162.409	270.427	-73.714	66.460	0.000
	q=2	374.511	89.569	284.941	-87.383	80.689	0.000
	q=3	332.631	62.948	269.683	-88.687	82.955	0.000
	q=4	301.836	50.630	251.207	-85.913	81.085	0.000
Carnivore *vs.* Omnivore	***q=0***	1021.000	1021.000	0.000	-21.950	19.662	0.964
	q=1	852.495	245.085	607.410	-97.115	95.879	0.000
	q=2	724.793	134.078	590.716	-116.986	117.711	0.000
	q=3	632.335	100.057	532.278	-122.329	124.300	0.000
	q=4	564.822	84.705	480.117	-120.788	123.657	0.000
Herbivore *vs.* Omnivore	***q=0***	616.000	616.000	0.000	-17.669	22.193	0.938
	q=1	514.548	185.467	329.081	-72.967	83.460	0.000
	q=2	418.459	104.959	313.500	-85.566	94.291	0.000
	q=3	333.172	74.144	259.028	-89.433	96.138	0.000
	q=4	266.474	57.992	208.483	-89.857	95.077	0.000
Percentage of Significant Differences (%)	***q=0***						0%
	q=1						100%
	q=2						100%
	q=3						100%
	q=4						100%

Table S6D. SDP (specificity diversity permutation) tests for the species category of the ES (enriched species) in the latter diet type

Treatments	Order	Former	Latter	Delta	Lower (95%)	Upper (95%)	*p*-Value
Carnivore *vs.* Herbivore	***q=0***	271.000	271.000	0.000	-13.212	11.112	0.877
	q=1	49.797	219.797	-170.001	-36.524	33.538	0.000
	q=2	17.874	174.080	-156.206	-38.546	35.854	0.000
	q=3	10.910	137.669	-126.759	-41.319	38.989	0.000
	q=4	8.583	112.063	-103.480	-42.396	40.397	0.000
Carnivore *vs.* Omnivore	***q=0***	615.000	615.000	0.000	-16.933	14.607	0.932
	q=1	162.561	512.088	-349.527	-94.208	90.534	0.000
	q=2	79.585	416.199	-336.614	-107.749	105.451	0.000
	q=3	54.654	340.858	-286.205	-100.298	99.107	0.000
	q=4	43.787	288.282	-244.494	-88.754	88.059	0.000
Herbivore *vs.* Omnivore	***q=0***	820.000	820.000	0.000	-19.485	28.341	0.960
	q=1	261.681	629.518	-367.836	-69.249	81.102	0.000
	q=2	143.017	472.440	-329.423	-80.247	90.184	0.000
	q=3	99.036	363.603	-264.567	-91.134	99.443	0.000
	q=4	78.040	293.536	-215.495	-98.225	105.409	0.000
Percentage of Significant Differences (%)	***q=0***						0%
	q=1						100%
	q=2						100%
	q=3						100%
	q=4						100%

Table S6E. SDP (specificity diversity permutation) tests for the species category of all species with significant differences

Treatments	Order	Former	Latter	Delta	Lower (95%)	Upper (95%)	*p*-Value
Carnivore *vs.* Herbivore	*q=0*	65283.000	8905.000	56378.000	-6371.652	2001.362	0.000
	q=1	21279.013	6064.387	15214.626	-5250.381	2938.079	0.000
	q=2	3793.633	3652.380	141.253	-4539.051	4072.802	0.949
	q=3	1712.345	2294.028	-581.683	-3190.845	3164.785	0.727
	q=4	1184.973	1697.743	-512.769	-2239.144	2259.353	0.655
Carnivore *vs.* Omnivore	*q=0*	65457.000	8936.000	56521.000	-4579.752	3849.320	0.000
	q=1	21189.861	5838.976	15350.885	-4025.501	3648.777	0.000
	q=2	3739.532	3961.514	-221.982	-3936.562	3885.819	0.901
	q=3	1691.223	2844.574	-1153.351	-3068.271	3108.319	0.448
	q=4	1171.901	2234.290	-1062.390	-2229.024	2275.208	0.347
Herbivore *vs.* Omnivore	*q=0*	10272.000	107310.000	-97038.000	-3969.745	9793.109	0.000
	q=1	6858.125	55668.931	-48810.806	-4316.177	8042.650	0.000
	q=2	4031.306	20024.189	-15992.883	-4878.621	6479.816	0.000
	q=3	2482.106	10205.146	-7723.041	-4308.476	4916.156	0.001
	q=4	1819.988	6964.297	-5144.310	-3388.009	3747.864	0.005
Percentage of Significant Differences (%)	*q=0*						100%
	q=1						100%
	q=2						33.30%
	q=3						33.30%
	q=4						33.30%

Table S6F. SDP (specificity diversity permutation) tests for all species (without considering species specificity)

Treatments	Order	Former	Latter	Delta	Lower (95%)	Upper (95%)	*p*-Value
Carnivore *vs.* Herbivore	*q=0*	73718.000	172486.000	-98768.000	-23890.077	2075.211	0.000
	q=1	24201.814	105054.960	-80853.147	-20274.519	5363.286	0.000
	q=2	4252.678	35426.712	-31174.034	-18722.896	13529.171	0.000
	q=3	1872.641	14041.400	-12168.759	-15482.652	14990.238	0.107
	q=4	1283.177	8524.776	-7241.600	-10712.566	10774.504	0.180
Carnivore *vs.* Omnivore	*q=0*	73718.000	118324.000	-44606.000	-15322.602	10690.876	0.000
	q=1	24201.814	61157.945	-36956.132	-14535.944	11196.670	0.000
	q=2	4252.678	22263.714	-18011.036	-16730.264	15318.296	0.023
	q=3	1872.641	11225.826	-9353.185	-15254.360	15048.154	0.235
	q=4	1283.177	7596.959	-6313.782	-10697.605	10710.964	0.260
Herbivore *vs.* Omnivore	*q=0*	172486.000	118324.000	54162.000	-4622.291	21805.431	0.000
	q=1	105054.960	61157.945	43897.015	-7202.085	18774.044	0.000
	q=2	35426.712	22263.714	13162.998	-13963.069	17744.825	0.114
	q=3	14041.400	11225.826	2815.574	-14500.208	14786.416	0.703
	q=4	8524.776	7596.959	927.817	-10280.577	10231.997	0.848
Percentage of Significant Differences (%)	*q=0*						100%
	q=1						100%
	q=2						66.70%
	q=3						0%
	q=4						0%

Table S7. The relationships between the phylogenetic timeline (PT) and AGM (animal gastrointestinal microbiome) specificity diversity (SD) (PTSD) for each diet type and for all diet types pooled together

**Diversity Order**	**Diet Type**	**PTSD: Log-Linear model (Power Law model)**			
		**ln(*a*)**	***b***	**Adjusted R^2**	***p*-value**
q = 0	Carnivore	6.618	-0.102	0.137	0.32
	Herbivore	8.441	-0.494	0.470	0.000
	Omnivore	6.773	-0.109	0.131	0.259
	All species	7.103	-0.187	0.213	0.003
q = 1	Carnivore	6.268	-0.094	0.13	0.344
	Herbivore	8.388	-0.549	0.518	0.000
	Omnivore	6.512	-0.107	0.137	0.237
	All species	6.856	-0.196	0.228	0.002
q = 2	Carnivore	6.072	-0.101	0.148	0.282
	Herbivore	8.333	-0.575	0.538	0.000
	Omnivore	6.335	-0.109	0.151	0.194
	All species	6.699	-0.205	0.246	0.001
q = 3	Carnivore	5.952	-0.108	0.163	0.236
	Herbivore	8.250	-0.580	0.543	0.000
	Omnivore	6.179	-0.101	0.145	0.211
	All species	6.572	-0.206	0.251	0.001

Table S8. Summary information on the AGM (animal gastrointestinal microbiome) datasets

**Group**	**Taxa or Diet Type**	**Mean Reads for Each Sample**	**Mean OTUs for Each Sample**	**Total Number of OTUs**	**Number of Samples**
Class	*Actinopteri*	33427	103	50097	1271
	Amphibia	8070	204	2844	21
	Arachnida	36521	115	3645	45
	Aves	28194	135	29298	503
	Chondrichthyes	34989	119	2711	32
	Chromadorea	52605	75	7470	215
	Insecta	32644	71	36312	979
	Malacostraca	126801	182	3744	30
	Mammalia	44766	243	207896	1499
	Sauropsida	29266	223	25720	308
Diet types	*Carnivore*	25397	118	73718	1474
	Herbivore	38804	178	172486	1621
	Omnivore	45297	164	118324	1505
Vertebrates *vs.* Invertebrates	*Invertebrates*	38389	76	49930	1269
	Vertebrates	36895	176	313884	3634
Species	*Apis mellifera*	13491	35	1223	231
	Bos taurus	10857	296	23171	105

Table S9. The number of AGM species in each species category, classified based on specificity permutation (SP) test with FDR (false discovery rate control) corrections at *P*-value=0.05 (This is a summary version of Table S1A for the convenience of discussion in the discussion section).

Pairwise Comparison (Former *vs*. Latter)	With Significant Differences in Specificity				Sum=(With Significant Difference in Specificity)	Without Significant Difference in Specificity	Total Species	% of Species with Significant Differences
	Unique in the former	Unique in the latter	Enriched in the former	Enriched in the latter				
Apis mellifera vs. *Bos taurus* (two representative species)								
Apis mellifera vs. Bos taurus	159	23171	0	0	23330	1064	24394	95.6%
A total of 45 pairwise comparisons of the AGMs between 10 host animal classes								
**Mean of the 45 Comparisons**	8220.8	13976.6	62.7	29.0	22289.2	51427.3	73716.5	30.2%
**Standard Error**	1933.2	5543.8	21.2	14.9	5629.9	10262.5	11864.5	
A total of 9 sequential (along the phylogenetic timeline) comparisons of the AGMs between 10 host animal classes								
**Mean**	11891.9	9427.9	71.2	70.6	21461.6	36480.0	57941.6	37.0%
**Standard Error**	5506.6	5018.2	44.3	67.7	6152.9	21720.7	23018.2	
Pairwise comparisons of the three diet types								
Carnivore *vs*. Herbivore	64500	8122	512	271	73405	168228	241633	30.4%
Carnivore *vs*. Omnivore	63821	7300	1021	615	72757	111688	184445	39.4%
Herbivore *vs*. Omnivore	8836	105874	616	820	116146	167073	283219	41.0%
Invertebrates vs. Vertebrates								
Invertebrates and Vertebrates	47632	2237	343	84	50296	311220	361516	13.9%

Table S10. The number of exclusively unique species (EUS) in each animal class

Host Animal Class	Exclusive Unique Species in Each Class
Arachnida	39
Malacostraca	54
Chondrichthyes	18
Actinopteri	26
Amphibia	100
Sauropsida	15
