# Supplementary material for: How Host Phylogeny and Diet Shape the Specificity and Specificity Diversity of Animal Gut Microbiomes
Source: Environ Microbiol Rep. 2026 Jan 29;18(1):e70253. doi: 10.1111/1758-2229.70253 (PMC12856063; doi:10.1111/1758-2229.70253)
Supplement: Supplementary file 3 — Data S3. SSD_code_AGM. [file EMI4-18-e70253-s001.zip › SSD_code_AGM/Help Document.docx]

**SSD.R Program Guide**

**Program Overview**
SSD.R implements the SSD framework first published in Ma ZS (2024) Species specificity and specificity diversity (SSD) framework: a novel method for detecting unique and enriched species associated with disease by leveraging microbiome heterogeneity. *BMC Biology*, 22, 283. <https://doi.org/10.1186/s12915-024-02024-7>

**Installation & Requirements**

- R version 3.6.3 or compatible
- The R script (SSD.R) and input data folder must be located in the same directory
- Required input files: OTU tables in tab-separated format

**Input File Specifications**

- Format: M×N matrix (M samples × N OTUs) of OTU reads (abundances)
- Separator: Tab character ("\t")
- Structure: First row contains OTU names, first column contains sample IDs
- Minimum: Two habitat files required (e.g., "Input_Data/Amphibia.txt" and "Input_Data/Malacostraca.txt")

**Execution**

1. Place SSD.R and input data folder in the same directory
2. Run from Linux shell using appropriate R script execution command

This implementation provides unified metrics for quantifying host-specificity at both taxon and community levels using the same underlying data structure.

**A specific example is shown below:**

**(1) Program Installation**

The R version used in this code is version R3.6.3. The R-script (SSD.R) and its input data (*e.g*., “Input_Data”) should be located in the same folder.

**(2) Input Files**

The input file is an OTU table, an MxN matrix with elements representing the number of OTU reads for each OTU in the community, separated by “\t” (Tab) symbol, where M is the number of community samples, and N is the number of OTUs. The first row lists the OTU names, and the first column lists the sample IDs or names.

Each file in the input data folder (e.g., "Input_Data") represents a habitat. There must be at least two files (habitats) in the input folder. For example, a folder might contain the files "Input_Data/Amphibia.txt" and "Input_Data/Malacostraca.txt".

**(3) Usage**

Copy the R script ("SSD.R") and the input data folder (e.g., "Input_Data") into the same directory. Run the program from the Linux shell with the following command:

$ Rscript SSD.R

**(4) Output files**

The output files from “SSD.R” program are stored in following three folders.

/Observed_Specificity/Observed_Specificity.txt /*Observed Specificity*/

/Species_Groups/ /*7 Species groups classified based on permutation tests*/

Unique species in Amphibia.txt

Unique species in Malacostraca.txt

Enriched species in Amphibia.txt

Enriched species in in Malacostraca.txt

Significantly different species.txt

Insignificantly different species.txt

Total species.txt

/Specificity_Diversity/

Specificity_Diversity.txt

/*Specificity diversity (SD) and permutation tests for the above 7 species groups*/
