# Supplementary material for: How Host Phylogeny and Diet Shape the Specificity and Specificity Diversity of Animal Gut Microbiomes
Source: Environ Microbiol Rep. 2026 Jan 29;18(1):e70253. doi: 10.1111/1758-2229.70253 (PMC12856063; doi:10.1111/1758-2229.70253)
Supplement: Supplementary file 3 — Data S3. SSD_code_AGM. [file EMI4-18-e70253-s001.zip › SSD_code_AGM/Help Document.pdf]

SSD.R Program Guide

Program Overview
SSD.R implements the SSD framework first published in Ma ZS (2024) Species specificity and specificity diversity (SSD) framework: a novel method for detecting unique and enriched species associated with disease by leveraging microbiome heterogeneity. *BMC Biology*, 22, 283. https://doi.org/10.1186/s12915-024-02024-7

Installation & Requirements

- R version 3.6.3 or compatible
- The R script (SSD.R) and input data folder must be located in the same directory
- Required input files: OTU tables in tab-separated format

Input File Specifications

- Format: M×N matrix (M samples × N OTUs) of OTU reads (abundances)
- Separator: Tab character ("\t")
- Structure: First row contains OTU names, first column contains sample IDs
- Minimum: Two habitat files required (e.g., "Input_Data/Amphibia.txt" and "Input_Data/Malacostraca.txt")

Execution

1. Place SSD.R and input data folder in the same directory
2. Run from Linux shell using appropriate R script execution command

This implementation provides unified metrics for quantifying host-specificity at both taxon and community levels using the same underlying data structure.

A specific example is shown below:

(1) Program Installation
The R version used in this code is version R3.6.3. The R-script (SSD.R) and its input data (*e.g.*, “Input_Data”) should be located in the same folder.

(2) Input Files
The input file is an OTU table, an MxN matrix with elements representing the number of OTU reads for each OTU in the community, separated by “\t” (Tab) symbol, where M is the number of community samples, and N is the number of OTUs. The first row lists the OTU names, and the first column lists the sample IDs or names.

Each file in the input data folder (e.g., "Input_Data") represents a habitat. There must be at least two files (habitats) in the input folder. For example, a folder might contain the files "Input_Data/Amphibia.txt" and "Input_Data/Malacostraca.txt".

(3) Usage

Copy the R script ("SSD.R") and the input data folder (e.g., "Input_Data") into the same directory. Run the program from the Linux shell with the following command:

```
$ Rscript SSD.R
```

(4) Output files

The output files from "SSD.R" program are stored in following three folders.

/Observed_Specificity/Observed_Specificity.txt /*Observed Specificity*/

/Species_Groups/ /*7 Species groups classified based on permutation tests*/

- Unique species in Amphibia.txt
- Unique species in Malacostraca.txt
- Enriched species in Amphibia.txt
- Enriched species in in Malacostraca.txt
- Significantly different species.txt
- Insignificantly different species.txt
- Total species.txt

/Specificity_Diversity/

- Specificity_Diversity.txt
  /*Specificity diversity (SD) and permutation tests for the above 7 species groups*/
